# Distinct methylomic signatures of high-altitude acclimatization and adaptation in the Tibetan Plateau

**DOI:** 10.1038/s41421-025-00795-z

**Published:** 2025-05-06

**Authors:** Feifei Cheng, Ren-Juan Shen, Zhili Zheng, Zhen Ji Chen, Peng-Juan Huang, Zhuo-Kun Feng, Xiaoman Li, Na Lin, Meiqin Zheng, Yuanbo Liang, Jia Qu, Fan Lu, Zi-Bing Jin, Jian Yang

**Affiliations:** 1 School of Life Sciences, Westlake University, Hangzhou, Zhejiang China; 2 Institute for Molecular Bioscience, The University of Queensland, Brisbane, Queensland Australia; 3 Beijing Institute of Ophthalmology, Beijing Tongren Hospital, Capital Medical University, Beijing, China; 4 School of Ophthalmology & Optometry, Wenzhou Medical University, Wenzhou, Zhejiang China; 5 Westlake Laboratory of Life Sciences and Biomedicine, Hangzhou, Zhejiang China

**Keywords:** Ageing, DNA methylation

## Abstract

High altitude presents a challenging environment for human settlement. DNA methylation is an essential epigenetic mechanism that responds to environmental stimuli, but its roles in high-altitude short-term acclimatization (STA) and long-term adaptation (LTA) are poorly understood. Here, we conducted a methylome-wide association study involving 687 native highlanders and 299 acclimatized newcomers in the Tibetan Plateau and 462 native lowlanders to identify differentially methylated sites (DMSs) associated with STA or LTA. We identified 93 and 4070 DMSs for STA and LTA, respectively, which had no overlap, showed opposite asymmetric effect size patterns, and resided near genes enriched in distinct biological pathways/processes (e.g., cell cycle for STA and immune diseases and calcium signalling pathway for LTA). Epigenetic clock analysis revealed evidence of accelerated ageing in the acclimatized newcomers compared to the native lowlanders. Our research provides novel insights into epigenetic regulation in relation to high altitude and intervention strategies for altitude-related ageing or illnesses.

## Introduction

The high-altitude environment, characterized by reduced atmospheric pressure (including oxygen partial pressure), decreased temperature, and increased ultraviolet radiation^1^, is challenging for human settlement. To cope with this harsh environment, acclimatized newcomers (ANs) react with physiological changes to restore homeostasis (e.g., enhanced ventilation and heart rate^2^) in a timescale from minutes to years, while native highlanders (NHs) have adapted genetically through thousands of years of evolution^3,4^, leading to an increased ability to survive and reproduce (e.g., infants born to NHs have higher birth weights than those born to ANs^2,5,6^). We refer to these two coping strategies as short-term acclimatization (STA) and long-term adaptation (LTA), respectively. Of note, specific STA changes that are temporarily beneficial could be detrimental in the long term. For instance, an increased hemoglobin concentration improves oxygen carrying ability in the short term but can increase the risk of chronic mountain sickness owing to its association with increased blood viscosity^7,8^. Investigating the molecular basis underlying STA and LTA is crucial for understanding how humans tolerate and survive adverse environmental conditions at high altitude, thereby advancing our knowledge of intervention strategies for altitude- or hypoxia-related illnesses.

Previous LTA studies in humans have mainly focused on genetic adaptation, and signatures of natural selection have been identified in several gene loci, including the hypoxia inducible factors (HIF) pathway genes, endothelial PAS domain protein 1 (*EPAS1*) and egl-9 family hypoxia inducible factor 1 (*EGLN1*)^4,9–12^. DNA methylation (DNAm) is an essential epigenetic machinery for regulating gene expression in response to environmental stimuli^13–15^, but its roles in STA and LTA are underexplored. Prior studies investigating genomic sites differentially methylated in NHs compared to their lowland counterparts are rare and have limited statistical power due to the small sample sizes ($n\, < \,40$ for NHs)^16,17^. For instance, two methylome-wide association studies (MWASs) in Ethiopian^16^ and Andean^17^ populations have identified only four and one differentially methylated sites (DMSs), respectively, and none of the DMSs reside in known adaptive loci. On the other hand, Everest base camp trek studies have found evidence of DNAm alterations associated with acute exposure to high altitude in HIF pathway genes^18^ and a few selected hypoxia-related genes^19^. Nevertheless, these studies also suffer from power limitations ($n\, < \,30$), and the methylomic characteristics of both LTA and STA remain elusive.

In this study, to comprehensively characterize changes in the human methylome during high-altitude acclimatization and adaptation, we performed an MWAS with 1448 unrelated Tibetan and Han Chinese participants. DMSs associated with STA were identified by contrasting 299 ANs (i.e., highland Han Chinese) to 462 native lowlanders (NLs, i.e., lowland Han Chinese). These two groups are genetically homogeneous but lived at different altitudes, meaning that the DMSs were driven by short-term environmental stimuli. DMSs associated with LTA were identified by contrasting 687 NHs (i.e., highland Tibetan Chinese) to 299 ANs (i.e., highland Han Chinese), reflecting DNAm changes due to long-term high-altitude exposure, genetic differentiation, and population-specific attributes such as dietary factor and lifestyle. Furthermore, we performed a methylation quantitative trait locus (meQTL) study and integrated the meQTL data to examine the role of genetic factors in epigenetic alterations. Additionally, the epigenetic clock^20,21^ was utilized to assess the effect of high-altitude environment on ageing. Altogether, our study reveals distinct methylomic signatures of acclimatization and adaptation and accelerated ageing of ANs in the Tibetan Plateau. Our findings provide novel insights into epigenetic regulations in relation to high altitude and intervention strategies of altitude-related illnesses and ageing.

## Results

### Study design and data overview

**Fig. 1:**
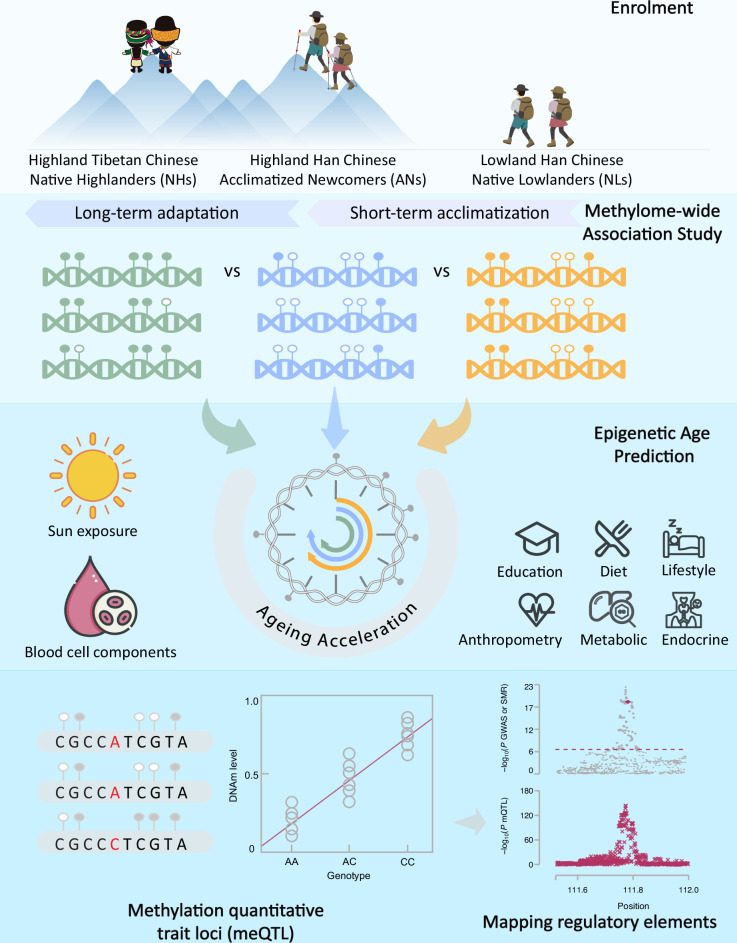
Schematic of the study design. First, we enrolled participants, namely, native highlanders (NHs, Highland Tibetan Chinese), acclimatized newcomers (ANs, Highland Han Chinese), and native lowlanders (NLs, Lowland Han Chinese). Second, we conducted MWAS to investigate DNAm alterations associated with high-altitude STA and LTA. DMSs of long-term adaptation were identified by comparing NHs and ANs. DMSs of short-term acclimatization were identified by comparing ANs and NLs. Third, epigenetic clock analysis was utilized to assess ageing acceleration in the three groups. Eight categories of behavioral and biomedical phenotypes were tested for associations with ageing acceleration in the subjects living in the Tibetan Plateau. Fourth, with the availability of genotype and DNAm data, we performed meQTL analysis and investigated the role of genetic differentiation in driving the LTA DMSs.

A schematic of the study design is illustrated in Fig. 1. This study takes the advantage of the Tibetan-Han Chinese high-altitude (THCH) cohort established by our previous study^4^, which consists of three groups: 918 NHs (highland Tibetan Chinese), 348 ANs (highland Han Chinese), and 488 NLs (lowland Han Chinese). Briefly, the highland and lowland samples were collected from participants who resided at ~4100 and ~9 m, respectively, above sea level. The ANs had resided on the Tibetan Plateau (TP) for a median duration of 3 years, with individual durations ranging from less than a half year to 63 years (Supplementary Fig. S1). The median age of all the participants was 38 years (ranging from 11 to 90), and females represented 74.6% of all participants (see below for the sensitivity analysis conducted exclusively with female participants). Details of demographic characteristics are recorded in Supplementary Table S1. After standard quality control (QC), ~5.1 million common genetic variants and 445,001 DNAm probes on 1448 unrelated participants (687 NHs, 299 ANs, and 462 NLs) were retained (Materials and methods).

### Identifying DMSs associated with high-altitude acclimatization or adaptation

**Fig. 2:**
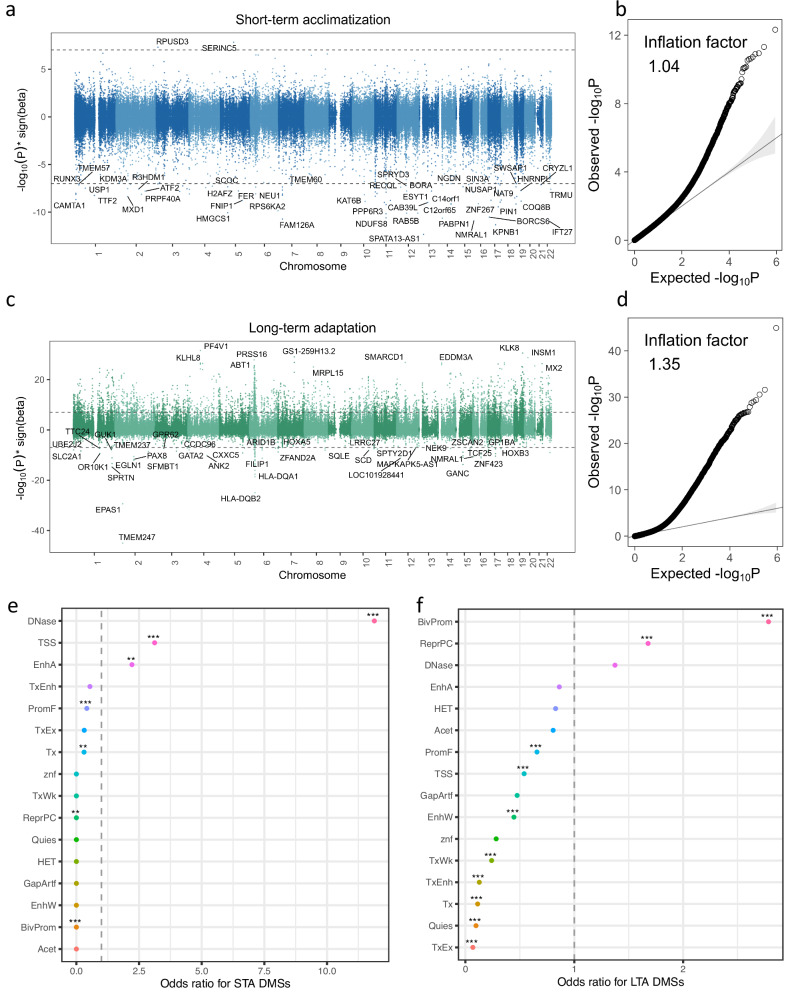
MWAS analyses for STA and LTA. **a**, **c** Manhattan plots of MWAS results for STA (**a**) and LTA (**c**). The *x*-axis denotes the genomic position, and the *y*-axis denotes the MWAS association $-{\mathrm{log}}_{10}$(bacon-adjusted ${P}$ value) times the sign of the effect size (the difference in the mean DNAm level of the target group from the control group). The dashed lines indicate the methylome-wide significance thresholds (bacon-adjusted ${P}$ value = $1.12\times {10}^{-7}$). DMSs were annotated to the nearest genes. **b**, **d** The quantile-quantile plot shows the observed $-{\mathrm{log}}_{10}$(bacon-adjusted ${P}$ value) in the *y*-axis against the expected distribution under the null in the *x*-axis for STA (**b**) and LTA (**d**). **e** Functional element enrichment for the STA DMSs. **f** Functional element enrichment for the LTA DMSs. The *y*-axis denotes the functional elements, and the *x*-axis represents the corresponding odds ratios. ** indicates ${P}$ value of permutation test < 0.01, and *** indicates ${P}$ value of permutation test < 0.001. The 16 genomic elements included assembly gaps and alignment artefacts (GapArtf), quiescent states (Quies), heterochromatin states (HET), polycomb repressed states (ReprPC), acetylations (Acet), weak candidate enhancers (EnhWk), active candidate enhancers (EnhA), transcribed candidate enhancers (TxEnh), weak transcription (TxWk), strong transcription (Tx), exons and transcription (TxEx), zinc finger gene states (znf), DNase I hypersensitivity (DNase), bivalent promoter states (BivProm), flanking promoter states (PromF), and transcription start site states (TSS).

We performed two MWAS analyses to identify DMSs associated with STA by comparing 299 ANs as the target group and 462 NLs as the control group and those associated with LTA by comparing 687 NHs as the target group and 299 ANs as the control group. For each DNAm probe, we used the linear model *DNAm-target/control group + covariates* to test for difference in the mean DNAm level of the target group from the control group. Hereinafter, we refer to this difference as effect size of acclimatization or adaptation on DNAm. We corrected the test statistics for uncontrolled confounding effects using *bacon*^22,23^. This strategy was chosen to control test statistic inflation without substantially compromising of statistical power (Supplementary Note S1, Table S2, Figs. S2–S4). Using this method, we identified 93 and 4070 non-overlapping DMSs for STA and LTA, respectively, at a ${P}$ value threshold of $1.12\times {10}^{-7}$ after Bonferroni correction for multiple testing (Fig. 2a–d; Supplementary Tables S3, S4). The number of quasi-independent DMSs was 76 for STA and 2402 for LTA using CoMeBack^24^ with an *r*^*2*^ threshold of 0.2. The LTA DMSs were significantly enriched in promoters (odds ratio (OR) = 2.78), repressed regions (OR = 1.68), and DNase hypersensitive sites (DHSs, OR = 1.37), and the STA DMSs were significantly enriched in DHSs (OR = 11.89), transcriptional start sites (OR = 3.12), and enhancers (OR = 2.22), compared to DNAm sites sampled repeatedly at random with matched distances to CpG islands (Fig. 2e, f). Intriguingly, the signs of the effect sizes showed asymmetric patterns for both STA and LTA, but in opposite directions. More specifically, most STA DMSs were hypomethylated, whereas most LTA DMSs were hypermethylated. These results suggest that there are completely distinct methylomic signatures for STA and LTA, implying that distinct biological mechanisms are involved in the two processes.

To explore the relevance of the DMSs to phenotypes, we mapped them to phenotypes based on data from the Epigenome-wide Association Studies (EWAS) Catalog^25^ (Supplementary Note S2, Tables S5, S6). The LTA DMSs were enriched in phenotypes related to survival in infancy (Supplementary Table S7), such as gestational age at birth (OR = 2.67, one-sided Fisher’s exact test $P=5.9\times {10}^{-23}$) and birth weight (OR = 2.58, $P=6.0\times {10}^{-6}$), in line with the previous finding that survival- and reproduction-related factors play a primary role in LTA^2,5,6,26,27^. Besides, according to gene annotation, some of the LTA DMSs are located in or near genes relevant to high-altitude adaptation, including the oxygen sensory and regulatory genes *EPAS1*, and *EGLN1*, the antioxidant enzyme-coding gene *SOD3*^17^, and the hemoglobin-related gene *TMPRSS6*^28^. We also observed LTA DMSs in or near growth factor-related genes, such as *EGFR*, and *BDNF*, which play important roles in developmental processes. In contrast, the STA DMSs were located near the genes *NDUFS8*, and *NMRAL1*, which are involved in the mitochondrial respiratory chain complex. To examine the robustness of the MWAS results, we performed a series of sensitivity analyses, including analyzing data from female participants only, adding predicted smoking status as a covariate^29^, or comparing MWAS results across different experimental batches. Both the STA and LTA MWAS results remained largely unchanged (Supplementary Notes S3, S4, Figs. S5-S7). We further conducted a sensitivity analysis by contrasting the 687 NHs (as the target group) to the 462 NLs (as the control group); the identified DMSs exhibited considerable overlap with either the LTA or the STA DMSs, and the overall pattern more closely resembled the LTA MWAS result (Supplementary Note S3, Table S8, Fig. S8).

### Enrichment of the DMSs in biological pathways and gene ontologies

**Fig. 3:**
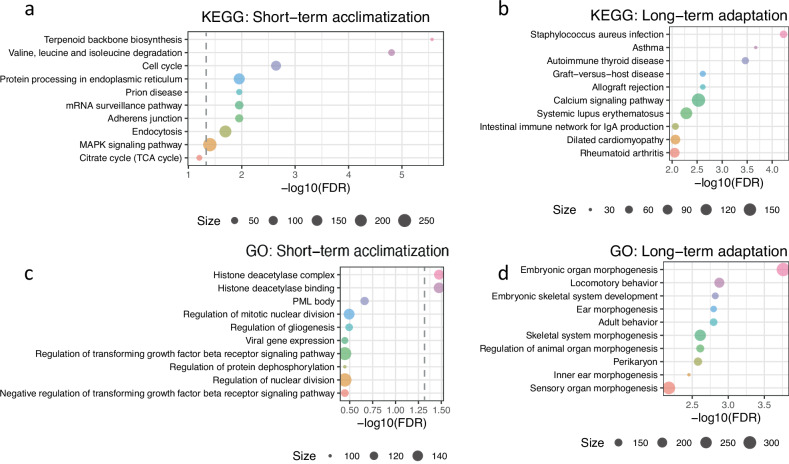
Gene-set enrichment for STA and LTA. **a**–**d** Bubble plots of the top 10 KEGG and GO terms ranked by the gene set enrichment analyses using the methylGSA R package. The *y*-axis denotes the biological pathways/processes, and the *x*-axis represents the enrichment significance $-{\mathrm{log}}_{10}$(false discovery rate (FDR)-adjusted *P* value). The size of the bubble indicates the number of genes in the set. The dashed line indicates an FDR threshold of 0.05. KEGG enrichment for STA (**a**). KEGG enrichment for LTA (**b**). GO enrichment for STA (**c**). GO enrichment for LTA (**d**).

To prioritize candidate biological pathways and processes involved in STA and LTA, we conducted gene set enrichment analysis (GSEA) on genes annotated to the DMSs. We noted that genes annotated to the STA DMSs were enriched in cell cycle-related pathways, including ‘cell cycle’ in Kyoto Encyclopedia of Genes and Genomes (KEGG) and ‘regulation of nuclear division’ and ‘regulation of mitotic nuclear division’ in Gene Ontology (GO) (Fig. 3a, c), suggesting that cell cycle-related processes play a major role in STA. This result was consistent with previous observations that hypoxia inhibits cell proliferation in most cell types to reduce oxygen consumption^30,31^ but conversely stimulates specialized cell types, such as vascular endothelial cells, to maintain oxygen homeostasis^32^. We also found evidence for the involvement of biological processes such as steroid biosynthesis, DNA repair, and cellular response to stress in STA, presumably related to hypoxia- and UV-induced oxidative stress^33–35^.

Regarding genes annotated to the LTA DMSs, the top enriched categories were immune diseases and calcium signaling pathways (Fig. 3b, d). The former category included KEGG pathways of infectious and autoimmune diseases. Since infected and inflamed tissues are often hypoxic, the cellular adaptive response to inflammation and infection might be shared with high-altitude adaptation^36–38^. The latter category included hypertrophic cardiomyopathy and cardiac muscle contraction among KEGG pathways and locomotory behavior, development, and morphogenesis among GO processes. This finding is in line with the transcriptomic study of yaks at high altitudes, in which the expression levels of calcium channels decrease whereas innate immunity becomes more active^39^.

Considering the asymmetric pattern of the signs of the effect sizes shown in both the STA and LTA MWASs (Fig. 2a, c), we tested the enrichment of genes annotated to the hypomethylated and hypermethylated DMSs separately. For STA, hypomethylated DMSs were enriched in steroid biosynthesis, protein processing, and the cell cycle, whereas hypermethylated DMSs were marginally enriched in cancer-related pathways, such as breast cancer and microRNAs in cancer. For LTA, the hypermethylated DMSs were enriched in the calcium signaling pathway and neuroactive ligand−receptor interaction, and the hypomethylated DMSs were enriched in immune-related diseases and haematopoietic cell lineage (Supplementary Note S5, Tables S9–S24). Altogether, our results suggest that genes annotated to STA and LTA DMSs are enriched in distinct biological processes, which are functionally relevant to the physiological characteristics of the two coping strategies.

### Identification and replication of meQTLs

**Fig. 4:**
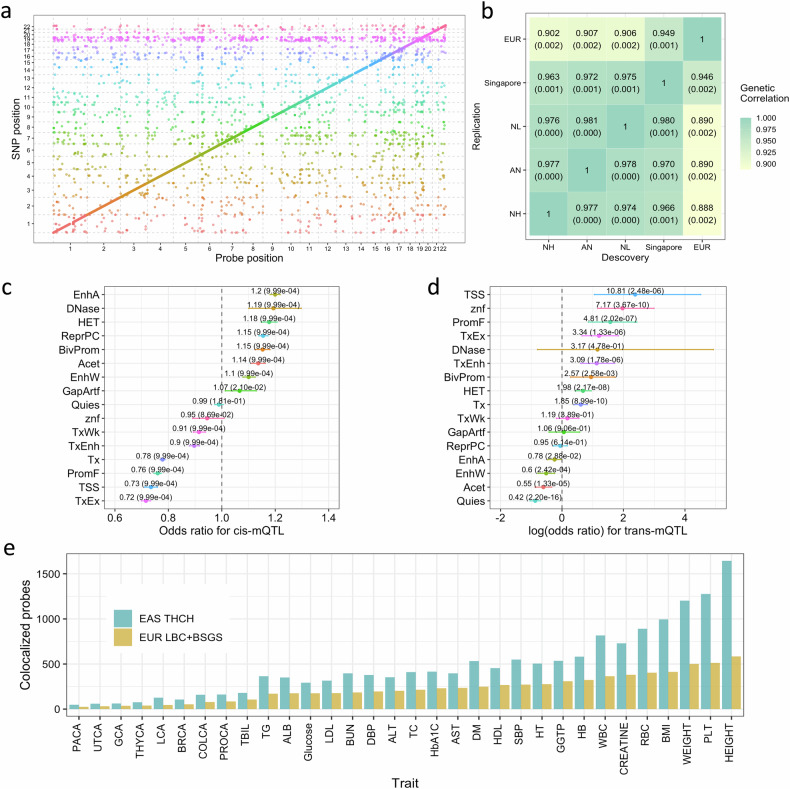
Characteristics of the THCH meQTLs and their performance in prioritizing DNAm probes associated with complex traits. **a** Position of *cis*- and *trans*-meQTLs across the genome. Each point represents an meQTL with the position of the CpG site on the *x*-axis and the SNP position on the *y*-axis, colored based on SNP chromosomes. The dashed lines indicate the chromosome boundary. The diagonal line shows the abundance of *cis*-meQTLs throughout the genome. **b** Heatmap of meQTL effect size correlations across datasets/populations. Heatmap color and texts in the block both show the estimates of ${r}_{b}$ with the corresponding standard error in parentheses. The top significant SNP-probe pairs (FDR adjusted *P* values *<* 0.05) in the discovery group and the matched pairs in the replication group were selected for estimating ${r}_{b}$. The analysis included three datasets/populations: the THCH data from the present study (Chinese, EAS), the Singapore iOmics data (Southeast Asians), and a meta-analysis of the Lothian Birth Cohort (LBC) and the Brisbane Systems Genetics Study (BSGS) data (EUR ancestry). **c** Enrichment of lead *cis*-meQTLs in genomic elements. Odds ratios are depicted with error bars indicating the 95% CI of the estimate. The labels are the odds ratio estimates with the enrichment test *P* values in parentheses. **d** Enrichment of lead *trans*-meQTLs in genomic elements. Log(odds ratios) are depicted with an error bar indicating the 95% CI of the estimate. The labels are the odds ratio estimates with the enrichment test *P* values in parentheses. The 16 genomic elements included assembly gaps and alignment artefacts (GapArtf), quiescent states (Quies), heterochromatin states (HET), polycomb repressed states (ReprPC), acetylations (Acet), weak candidate enhancers (EnhWk), active candidate enhancers (EnhA), transcribed candidate enhancers (TxEnh), weak transcription (TxWk), strong transcription (Tx), exons and transcription (TxEx), zinc finger gene states (znf), DNase I hypersensitivity (DNase), bivalent promoter states (BivProm), flanking promoter states (PromF), and transcription start site states (TSS). **e** Bar plot of the number of DNAm probes associated with 32 complex traits from the EAS GWAS summary data. The *x*-axis shows the acronyms for the traits, and the *y*-axis shows the number of DNAm probes identified with COLOC PP4 statistics > 0.8 using the THCH or LBC *+* BSGS meQTL data.

Having observed LTA DMSs in known adaptive genetic loci, we next investigated whether the DMSs identified at those loci were driven by genetic control of DNAm. To do so, we first linked genetic variants to DNAm sites by performing an meQTL mapping analysis of 445,001 DNAm probes on 1448 unrelated individuals in the THCH cohort. Genetic variants associated with a DNAm probe were defined as *cis*-meQTLs if located within 1 Mb of the probe and *trans*-meQTLs if located more than 5 Mb from the probe. We identified 13,464,132 *cis*-meQTLs with $P < 1\times {10}^{-8}$ for 88,780 probes, and 330,624 *trans*-meQTLs with $P < 1\times {10}^{-14}$ for 2394 probes (Fig. 4a; Supplementary Note S6, Table S25, Figs. S9-S11). The *cis*-meQTLs were significantly enriched in active enhancer and polycomb repressed regions and depleted in transcribed regions, and the *trans*-meQTLs were significantly enriched in transcription start sites and depleted in enhancer regions (Fig. 4c, d). We observed a 6.24-fold enrichment of *trans*-meQTLs (95% CI: 3.86−10.71) in the telomere and subtelomere regions (1 Mb at both ends of each chromosome), consistent with previous studies^40–43^.

To validate the quality of our data, we replicated the THCH meQTLs in a published European (EUR) meQTL dataset^44^. The replication rates were 0.87 and 0.82 for the *cis*- and *trans*-meQTLs, respectively, after Bonferroni correction for multiple testing in the replication analysis. Recognizing that the replication rate depends on replication sample size, we quantified the correlation of the meQTL effect sizes in the two datasets using the ${r}_{b}$ method^45^, which accounts for sampling variance in the estimated meQTL effects in both datasets. The correlation was 0.915 (standard error, SE = 0.001) for the top *cis*-meQTLs and 0.914 (SE = 0.006) for the top *trans*-meQTLs (Fig. 4b; Supplementary Fig. S12). Together, these findings indicate a strong resemblance of meQTLs between the EUR and East Asian (EAS) populations.

Finally, we demonstrated the effectiveness of leveraging the meQTLs to identify functional elements underlying genome-wide association study (GWAS) signals for complex traits and diseases in samples of EAS ancestry. GWAS summary statistics were from meta-analyses of Biobank Japan^46^ and Korean Biobank^47^ data for 32 traits (Supplementary Table S26). Integrating our meQTL summary statistics identified approximately twice as many colocalized meQTL and GWAS signals compared to a similar analysis using European meQTL data with a comparable sample size (Fig. 4e; Supplementary Note S7, Fig. S13). These results suggest that although the effect sizes of the top meQTLs are highly correlated between EAS (as represented by our data) and EUR, using an ancestry-matched meQTL dataset can still be beneficial for enhancing the detection of meQTL signals that colocalize with GWAS signals, likely because of the substantial differences in minor allele frequencies (MAFs) and linkage disequilibrium (LD) between EAS and EUR (Supplementary Figs. S14, S15).

### The role of genetic differentiation in driving the LTA DMSs

We next investigated the role of genetic differentiation in high-altitude epigenetic alterations from three different aspects. First, we performed an enrichment analysis to test whether the *cis*-meQTLs for the LTA DMSs are enriched in genetic variants associated with high-altitude adaptation, i.e., variants that are more differentiated in frequency between the NHs and the lowland EASs than expected by genetic drift, as identified in our previous study^4^. The lead meQTL variants of the LTA DMSs were 5.67-fold more likely to be associated with high-altitude adaptation at $P < 1\times {10}^{-5}$ compared to 1000 sets of randomly sampled, MAF-matched variants (enrichment test $P\, < \,0.001$), indicating that genetic variants associated with the LTA DMSs are more likely to be associated with high altitude adaptation. If the difference in mean DNAm level of a site between the ANs and NHs (denoted by ${\Delta }_{y}$) is partly attributed to the allele frequency difference of its meQTL variant (denoted by ${\Delta }_{p}$), then ${\Delta }_{y}$ is expected to correlate with $2{\Delta }_{p}b$, where $b$ represents the meQTL effect. In our data, we indeed observed a significant association between ${\Delta }_{y}$ and $2{\Delta }_{p}b$ across the LTA DMSs ($P=2.8\times {10}^{-8}$), where ${\Delta }_{p}$ and $b$ were estimated using the lead meQTL variant.

**Fig. 5:**
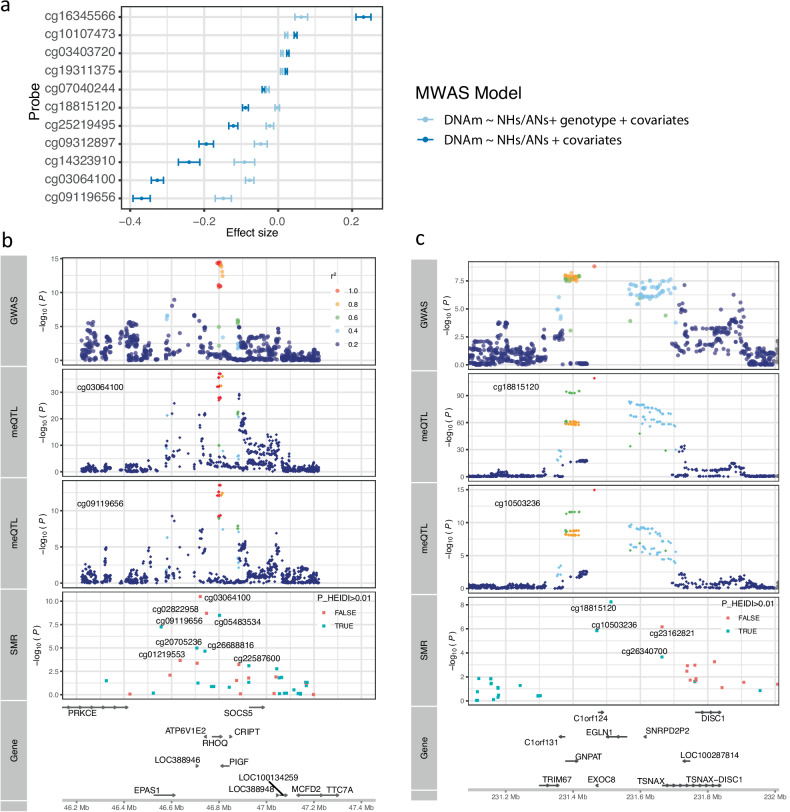
The role of genetic differentiation in driving the LTA DMSs. **a** MWAS analysis upon adding a covariate of the lead variant in the known adaptative loci. The *y*-axis denotes LTA DMSs that are annotated to the known adaptive loci, such as *EGLN1*, *EPAS1*, and *HLA-DQB1*, and the *x*-axis represents the effect size for MWAS analysis with error bars representing standard errors. Colors indicate two different models before/after adding the lead variant as a covariate. **b**, **c** Identification of genetic loci in which meQTL and high-altitude genetic adaptation signals share the same causal variants using SMR analysis. The *x*-axis represents the genomic positions, and the *y*-axes display -log_10_($P$-value) for GWAS, meQTL and SMR analyses in the indicated tracks. *EPAS1* region in the chromosome 2 (**b**). *EGLN1* region in the chromosome 1 (**c**).

Second, we examined whether the LTA DMSs in the known adaptive loci are due to genetic differentiation of the variants affecting DNAm. When the lead variant in a known adaptive locus was fitted as a covariate in the MWAS analysis for the LTA DMSs nearby, the strengths of the LTA signals around *EGLN1*, *EPAS1* and *HLA-DQB1* decreased substantially (Fig. 5a; Supplementary Table S27). For example, conditioning on the previously identified lead adaptive variant rs78561501^4^, the effect size of the probe near *EGLN1* (cg07040244) decreased from 0.039 ($P=1.0\times {10}^{-20}$) to 0.028 ($P=1.3\times {10}^{-11}$).

Third, we performed a colocalization analysis to identify genetic loci where the meQTL and putative high-altitude genetic adaptation signals share the same causal variant(s). There were 6 probes that achieved a methylome-wide significance level in the SMR analysis and met a PP4 threshold of 0.8 in the COLOC analysis (Supplementary Table S28). For instance, the strongest genetic adaptation signal (*EPAS1*) colocalized with meQTL signals for cg03064100 and cg05483534 (${\rm{P}}{\rm{P}}4\, > \,0.95$), of which cg03064100 was also an LTA DMS (${P}_{{\rm{MWAS}}}=1.4\times {10}^{-45}$) in an enhancer region (Fig. 5b). The second strongest high-altitude genetic adaptation signal (*EGLN1*) was colocalized with meQTL signals for cg18815120 and cg10503236 (${\rm{PP}}4=0.99$), of which cg18815120 was also an LTA DMS (${P}_{{\rm{MWAS}}}=5.8\times {10}^{-15}$) (Fig. 5c).

The results above suggest that the epigenetic adaptation signatures in the two loci were driven, at least partly, by genetic adaptation. Considering that the lead adaptive variants in the *EGLN1* and *EPAS1* loci have been reported to be associated with hemoglobin concentration (HGB)^4,9,12^, we tested whether DNAm level in these two loci was associated with HGB. Both sites showed a significant association with HGB after controlling for sex, age, and residential site (cg18815120: $\beta =18.34$, $P=6.2\times {10}^{-3}$; cg03064100: $\beta =14.65$, $P=7.0\times {10}^{-7}$), suggesting a potential mechanism whereby the adaptive variant may influence fitness-related phenotypes through epigenetic regulation.

### Accelerated ageing of the acclimated newcomers

**Fig. 6:**
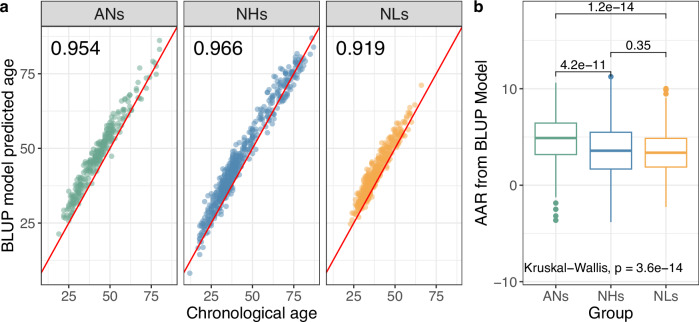
Epigenetic age prediction. **a** Scatter plots of chronological age (*y*-axis) against the BLUP model-predicted age (*x*-axis). Each dot represents an individual, and each panel represents a group in the THCH cohort. The red line represents a slope of 1 and an intercept of 0. The number in the top-left of the panel is the coefficient of determination (*R*^2^). **b** Box plot of the AARs of the three groups. The *P* values above the box represent the statistical significance of the pairwise differences in mean AAR between the two groups indicated. The $P$-value below the box represents the significance of the difference in mean AAR among all the three groups.

The methylomic profile of an individual changes over time and has been widely used as an indicator of ageing^20,48,49^. The unique design of this study enabled us to investigate the effect of high-altitude exposure on ageing during STA and LTA. We employed the Best Linear Unbiased Prediction (BLUP) model developed by Zhang et al.^50^, the most accurate model for epigenetic age prediction to date, to predict epigenetic ages of our participants based on their DNAm data. The predicted age and chronological age were highly correlated, with a squared correlation (*R*^*2*^) of 0.97 between the predicted and chronological ages in the NHs, 0.95 in the ANs, and 0.92 in the NLs (Fig. 6a), indicating the applicability of the previously established epigenetic age predictor in the THCH cohort. We refer to the difference between the predicted and chronological ages as age acceleration residual (AAR). We found a significant difference in AAR among the three groups ($P=3.6\times {10}^{-14}$; Fig. 6b): the mean AAR of the ANs was 1.3 years larger than that of the NHs ($P=4.2\times {10}^{-11}$) or NLs ($P=1.2\times {10}^{-14}$), but there was no significant difference between the NH and NL groups ($P=0.35$). Consistent results were observed in sensitivity analyses that were conducted to account for potential biases due to differences in gender ratio, mean chronological age among cohorts, and batch effects (Supplementary Note S8). Other commonly used epigenetic age predictors, such as those from Horvath et al.^48^, Hannum et al.^51^, and Levine et al.^49^, were less accurate than BLUP (*R*^*2*^ from 0.77 to 0.94 in the ANs) but consistently showed a larger mean AAR in the ANs than the NLs (Supplementary Note S9, Fig. S16). These results suggest strong evidence of ageing acceleration in the ANs compared to the NLs. There was no significant association between AAR and acclimatization time in the ANs ($P=0.45$), implying that the changes in DNAm due to acclimatization occurred over a relatively short period of time.

To identify risk factors involved in ageing acceleration, we tested the associations of AAR with 90 phenotypes classified into eight categories, namely, sun exposure, lifestyle, education, diet, anthropometry, blood cell components, metabolic traits, and endocrine factors in the ANs and NHs, respectively (Supplementary Tables S29, S30). Association analysis was performed for each risk category using ANOVA with age and age squared fitted as covariates. We found that sun exposure and blood cell components were significantly associated with AAR in the NHs, with $P=3.1\times {10}^{-3}$ and 2.4$\times {10}^{-3}$, respectively. However, these associations were not statistically significant in the ANs, possibly due to smaller sample size or heterogeneity in the effects of risk factors on DNAm between ANs and NHs (Supplementary Table S30). Moreover, we investigated whether variation in AAR among individuals is partly due to genetic factors by performing a GWAS for AAR but did not observe any signal with genome-wide significance (Supplementary Note S10, Figure S17), possibly due to the lack of statistical power to detect the polygenic effects for AAR. Additionally, MWAS analyses for AAR and environmental exposures suggest that the number of DNAm sites and correspondingly the number of genes involved in the response to environmental changes and ageing are likely to be large (Supplementary Figs. S18, S19). Taken together, these results provide important insights into potential interventions and anti-ageing strategies for high-altitude exposure.

## Discussion

This study advances our understanding of epigenetic acclimatization and adaptation to high-altitude exposure. With a unique experimental design (i.e., a large-scale population-based cohort including NHs, ANs, and NLs), we were able to delineate DNAm alterations attributable to short-term high-altitude exposure and those resulting from long-term high-altitude exposure, genetic differentiation, and population-specific characteristics. We identified 93 and 4070 DMSs for STA and LTA, respectively. The significant disparity in the number of DMSs between STA and LTA is unlikely affected by known confounding factors, such as age, sex, and experimental batches, as demonstrated by simulations (Supplementary Note S1) and sensitivity analyses (Supplementary Note S3). Instead, the large number of DMSs observed in LTA can be attributed to several factors: (1) Genetic differentiation. We observed a significant association between the mean DNAm difference between ANs and NHs and the allele frequency difference of the lead meQTL variant for the LTA DMSs, suggesting a role of genetic differentiation in driving the DMSs, although disentangling natural selection from genetic drift remains challenging. Furthermore, we found that epigenetic alterations in known adaptive loci, such as *EPAS1* and *EGLN1*, were driven at least in part by genetic adaptation. These genetic signals are influential in shaping DNAm, potentially governing the regulation of gene expression levels that are implicated in adaptative phenotypes. (2) Long-term high-altitude environmental stimuli. Prior work has indicated that chronic, sustained hypoxia can induce changes in DNAm patterns^14^. These high-altitude environmental-induced epigenetic changes might facilitate specific genetic mutations, leading to an interplay between genetic and epigenetic adaptations to high-altitude conditions^52^. (3) Population-specific attributes, such as diet and lifestyle. Tibetans consume a high-energy diet rich in coarse grains, meat, and yak butter, with less intake of refined grains, vegetables, and fruits than Han. This contrast in dietary and lifestyle patterns between the two populations likely shapes some of the observed methylomic signals^53,54^.

There was no overlap observed between the STA and LTA DMSs, indicating distinct epigenomic changes and, consequently, distinct biological mechanisms underlying high-altitude acclimatization and adaptation. It is plausible that, while some pathways are shared between STA and LTA, the genes involved may differ. This observation aligns with the distinct physiological responses and transcriptome profiles to high altitudes that have been documented in studies comparing STA to LTA^2,55,56^. For example, delayed growth and higher prevalence of underweight and stunting have been reported in high-altitude populations^57,58^. Tibetan males have a deeper chest, and Tibetan females have a wider chest than Han^59^, evidence of morphological adaptation to obtain better oxygen delivery. In terms of transcriptomic analysis, a recent study identified 1487 differentially expressed genes in placental samples when comparing 47 Tibetans to 19 immigrant Han^60^. Additionally, the signs of the effect sizes of STA and LTA DMSs showed asymmetric patterns in opposite directions. The asymmetric pattern of DMSs is not unexpected, as global hypomethylation is common in tumors and certain temporal states (e.g., after short-term exercise and during early pregnancy)^61–64^, whereas hypermethylation is often observed in response to long-term exposures to adverse environmental stimuli (e.g., particulate air pollution and toxin exposure)^65,66^. The effect size pattern of STA is consistent with a previous Everest base camp trek study, which suggests a significantly lower methylation level of the repetitive element LINE-1 at high altitude, a measurement of global methylation^19^. Based on the GSEA results, we prioritized candidate biological processes and discussed their functional relevance with high-altitude acclimatization or adaptation (Supplementary Note S9). Considering that DNAm often suppresses the expression of the target gene^67,68^, we hypothesize that cell division in blood and steroid biosynthesis are enhanced in STA, while physical development and locomotory behavior are suppressed in LTA, possibly through the regulation of the calcium signaling pathway. It is noteworthy that previous transcriptome studies examining short-term hypobaric hypoxia exposure have reported altered gene expression^69^, including *EGLN1*. While these studies have primarily focused on in vitro experiments (e.g., using cell lines), our study provides insights into in vivo epigenetic alterations at population level under hypoxic conditions. It is important to note that identifying the target genes associated with these DMSs may require the availability of corresponding transcriptomic data. An integrated analysis of both methylomic and transcriptomic data would offer more comprehensive insights into the molecular mechanisms underlying the identified DMSs.

Previous research has shown accelerated epigenetic ageing among NHs, specifically in Andean Quechua individuals who were born and raised at high altitude compared to those at low altitude^17^. While we did not include NHs born and raised at low altitude in our study, we demonstrated accelerated epigenetic ageing in ANs compared to NLs among Han individuals. These findings from both studies suggest that epigenetic ageing accelerates with an increase in the altitude of habitation within a population. A body of literature has reported a range of adaptive challenges for lowland individuals living at high altitude, such as an increased incidence of preeclampsia, reduced birth weight, lower maximal exercise performance, and higher prevalence of chronic mountain sickness^2,70–72^. These manifestations of maladaptation may reflect or contribute to ageing acceleration, as ageing is regarded as a process of declining adaptive homeostasis^73,74^. Whether the accelerated ageing of ANs is reversible when the adverse stimuli disappear would be an interesting question for future research. Furthermore, by leveraging the phenotypic variation among the highland individuals, we found that sun exposure and blood cell components were significantly associated with AAR, in line with oxidative stress (induced by UV exposure) and inflammation (reflected by blood cell components) as two major factors for ageing^75–77^. These findings are crucial for gaining insights into the biology of ageing and for informing clinical decision-making, especially in the development of preventative medicine strategies for ANs.

We performed an meQTL study and identified 13,464,132 *cis*-meQTLs for 88,780 probes and 330,624 *trans*-meQTLs for 2394 probes. Despite the strong correlation between the EAS and EUR meQTL effects, the EAS meQTLs exhibited substantially more power in detecting DNAm sites mediating the EAS GWAS effects than EUR meQTLs with a comparable sample size, likely because of the large differences in both MAF and LD between EAS and EUR. These data provide a unique resource for investigating the genetic control of DNAm in high-altitude adaptation and, more generally, the epigenetic mechanisms through which the genetic variants identified from GWAS exert effects on traits and disease risks in EAS populations.

Our results need to be interpreted with caution. First, response to hypoxia can vary with severity^78–80^; for instance, moderate hypoxia might have beneficial consequences such as enhanced spatial learning and memory, while severe/chronic hypoxia can impair spatial working memory^78–80^. Therefore, responses that are adaptive to severe/chronic hypoxia may differ from responses to moderate hypoxia. Second, epigenetic alterations in the high-altitude environment can be genotype dependent. Highland-adapted populations such as those in Tibet and the Andes show differences in adaptation strategies, including in adaptive genetic mechanisms^2,27^, which would give rise to differences in epigenetic adaptation. Third, adaptive DNAm patterns can be tissue-specific, and our observed methylomic signatures are limited in whole blood. Additional research is needed to confirm our findings in other tissues. Fourth, this study was restricted in the DNAm sites available on the Illumina 450 K DNAm array. Data from large-scale studies, using DNAm arrays with higher coverage or DNAm sequencing technologies that interrogate the entire genome in an unbiased manner, are expected in the future^81,82^. Altogether, epigenomic responses at a range of altitudes, in different highland populations and/or various tissues with advanced techniques warrant further investigation. The study could be further improved by incorporating additional information to investigate epigenetic changes attributed to individuals being born or raised in high-altitude environment^17,83^. Moreover, the duration of high-altitude residency among Han migrants may influence the statistical power to detect DMSs when high-altitude exposure is a causal factor for these DMSs, and the effect is time-dependent. Nevertheless, our analysis mitigated this potential impact by grouping NHs, ANs, or NLs. Thus, the within group variation in high-altitude living time for ANs does not affect our findings.

In conclusion, by leveraging the unique experimental design and large sample sizes, we reveal distinct patterns of methylomic changes in high-altitude acclimatization and adaptation. The patterns indicate distinct coping mechanisms underpinning STA and LTA, as well as cohort-specific characteristics, such as genetic differentiation, lifestyle, and diet. Additionally, our findings on ageing acceleration may help develop preventive and therapeutic interventions for high-altitude-induced ageing or maladaptation.

## Materials and methods

### Participants and sample collection

The recruitment of participants has been described previously^4^. Both NHs and ANs lived at ~ 4100 m above sea level, with 1060 participants from Seda and 90 participants from Litang in Sichuan, China, while the NLs resided at ~9 m above sea level in Wenzhou, China. Written informed consent was obtained for each participant, and the study was approved by the Ethics Committee of Wenzhou Medical University (approval numbers KYK-2013-19 and 2022-123-K-94-02) and Ethics Committee of Westlake University (approval number 20200722YJ001). The THCH cohort consisted of three groups: 918 NHs (highland Tibetan Chinese), 348 ANs (highland Han Chinese) and 488 NLs (lowland Han Chinese) for whom both DNAm and genotype data were available. The ancestry was verified by both self-reports and principal component analysis (PCA) of SNP genotypes (Supplementary Fig. S20). Peripheral blood samples were obtained for SNP genotyping and DNAm quantification. For highland subjects, peripheral blood samples were also used for clinical blood tests, including complete blood count and biochemical analyses. Questionnaire interviews were conducted by well-trained investigators with the help of native translators.

### Quantification of DNAm and quality control

Three experimental batches were processed in the same laboratory under the same temperature (22 °C) by the same technical personnel. The procedure was performed according to the manufacture’s protocol. Genomic DNA was extracted from peripheral blood (Simgen Blood DNA Mini Kit; Simgen), and its concentrations were measured with a NanoDrop 1000 spectrophotometer (Thermo Scientific). DNAm was quantified and profiled using Illumina HumanMethylation450 BeadChips (HM450) and scanned by iScan Reader (Illumina Inc.), resulting in 485,512 CpG sites across the genome. The quality control (QC) and quantile normalization procedures were performed using the *Meffil* package (version 1.0.0)^84^ in R (version 4.1.2). We removed the outliers based on genotype concordance, gender concordance, and methylation quality with parameters of beadnum.samples.threshold = 0.1, detectionp.samples.threshold = 0.1, detectionp.cpgs.threshold = 0.1, beadnum.cpgs.threshold = 0.1, sex.outlier.sd = 5, snp.concordance.threshold = 0.95, and sample.genotype.concordance.threshold = 0.8. Overall, 1448 unrelated subjects and 445,001 autosomal probes were retained after QC. We performed functional normalization to adjust for the technique effects using the first 20 PCs of control probes^85^. The normalized intensity was then converted to a beta value using the equation $\beta \,={M}/(M+U+100)$, where *M* is the methylated intensity and *U* is the unmethylated intensity^86^. Cell composition was estimated by the Houseman algorithm^87^ with the blood gse35069 cell type methylation profile as a reference using *Meffil*^84^.

### MWAS

For STA, we coded ANs as the target group and NLs as the control group; for LTA, we coded NHs as the target group and ANs as the control group. Linear regression was performed at each DNAm site to test for the difference in the mean DNAm level between the target group and the control group, with adjustment for the covariates of sex, age, experimental batch, slide, Sentrix row, Sentrix column, and estimated cell composition (${DNAm}_{i}\,\sim\,target/control\,group+sex+age+experimental\,batch+array\,position+estimated\,cell\,components$). We then corrected the test statistics for the remaining inflation and/or bias using the *bacon* R package^22^. DMSs were identified at the Bonferroni ${P}$ value threshold of $1.12\times {10}^{-7}$ (0.05/445,001). Manhattan and quantile‒quantile (Q‒Q) plots were visualized using the *ggplot2* R package. The inflation factor (λ) was calculated to quantify confounding in the methylome-wide association tests^88^. Sensitivity analyses for potential confounders (e.g., gender, batch, smoking status) are presented in Supplementary Note S3. The tests for DMS enrichment in the EWAS Catalog database^25^ were performed using Fisher’s exact test for the traits of gestational age, birth weight, hypertensive disorders of pregnancy, preeclampsia, alcohol consumption, BMI, smoking (and schizophrenia as a negative control). The enrichment test in functional elements using full-stack annotations predicted from large-scale datasets^89^. The 16 genomic elements included assembly gaps and alignment artefacts (GapArtf), quiescent states (Quies), heterochromatin states (HET), polycomb repressed states (ReprPC), acetylations (Acet), weak candidate enhancers (EnhWk), active candidate enhancers (EnhA), transcribed candidate enhancers (TxEnh), weak transcription (TxWk), strong transcription (Tx), exons and transcription (TxEx), zinc finger gene states (znf), DNase I hypersensitivity (DNase), bivalent promoter states (BivProm), flanking promoter states (PromF), and transcription start site states (TSS). We compared DMSs with the null distribution through 1000 samplings from all tested DNAm probes with matched distances to CpG islands (i.e., CpG island, shore, shelf, and open sea).

### GSEA of the MWAS results

KEGG and GO GSEA based on annotated genes of DMSs were carried out using the *methylGSA*^90^ and *missMethyl*^91^ R packages, accounting for the number of CpGs assigned to each gene. The *methylGSA* is a functional class scoring method, requiring only the input of $P$ values of all tested probes. We input the *bacon*-adjusted ${P}$ values and restricted GO/KEGG categories that contained at least 10 and at most 500 genes. For *missMethyl*, we used overrepresentation analysis, setting all tested probes as background and selecting significant probes in three scenarios, respectively: (1) probes with a bacon-adjusted $P$ value < 0.001; (2) probes with a bacon-adjusted $P$ value < 0.001 and positive effect sizes (hypermethylated signals); and (3) probes with a bacon-adjusted ${P}$ value < 0.001 and negative effect sizes (hypomethylated signals). A stringent bacon-adjusted ${P}$ value threshold of $1.12\times {10}^{-7}$ was also set to check the consistency of the enrichment results. The threshold of enrichment significance was set at a false discovery rate (FDR) of 0.05.

### Genotype imputation and QC

The genotyping process was performed as previously described^4^. The cleaned genotype data were imputed to the ChinaMAP reference panel using the online ChinaMAP imputation server (GRCh38)^92^. After imputation, we removed monomorphic SNPs, SNPs with HWE $P$ value < 1 × 10^−6^, SNPs with IMPUTE-INFO < 0.3, or SNPs with MAF < 0.05 in each group and retained ~5.1 million SNPs for the follow-up analysis. We estimated that pairwise genetic relationship using all autosome genetic variants (MAF ≥ 0.01) and removed one of a pair of individuals with estimated relatedness larger than 0.05 with GCTA-GRM software^93^. Ancestry information was verified by PCA based on the imputed common genotypes using GCTA-PCA^93^. We also performed genotype phasing using v2.0.5 of EAGLE2^94^ + PBWT^95^ and imputation against the 1000 Genomes Phase 3 reference panel for comparison (Supplementary Note S11).

### meQTL mapping

We performed meQTL mapping to identify proximal (*cis*-) and distal (*trans*-) significant associations between genetic variants and DNAm. DNAm probes were first preadjusted by sex, age, experimental batch, slide, Sentrix row, Sentrix column, and estimated cell composition using linear regression. The residuals were then normalized to a mean of 0 and variance of 1 using rank-based inverse normal transformation (RINT) in each subgroup. In the analysis of the full cohort, we merged the standardized phenotypes in each group and performed meQTL analysis. QTLtools (version 1.3.1) was used to perform the association tests and adjust for multiple testing in a permutation scheme^96^.

Specific to *cis*-meQTLs, the mapping window was defined as 1 Mb upstream and downstream around the tested DNAm site (significance was set at a nominal $P$ value threshold of $1\times {10}^{-8}$)^43^. For meQTL effect size correlation and functional enrichment, we used a relatively lenient and tailored $P$ value threshold. Specifically, we generated a null distribution of nominal $P$ values of the top meQTL via 1000 permutations for each probe. Then, the top nominal $P$ value was adjusted using a fitted beta distribution under the null to account for multiple testing of SNPs in the *cis* region of the probe. The adjusted $P$ values of all DNAm probes were further adjusted by FDR using the *qvalue* R package^97^ to account for multiple testing of probes across the genome. The threshold was set at an FDR adjusted $P$ value < 0.05, corresponding to a nominal $P$ value threshold of $2.2\times {10}^{-5}$, on average.

Regarding *trans*-meQTLs, the mapping window consisted of the whole genome, excluding 5 Mb upstream and downstream around the tested DNAm site (significance was set at a nominal $P$ value threshold of $1\times {10}^{-14}$)^43^. Similarly, for the follow-up analysis of meQTL effect size correlation and functional enrichment, we used a relatively lenient and tailored $P$ value threshold. We performed approximate permutation by sampling 1000 phenotypes^96^. We permuted 1000 randomly selected phenotypes and retained the smallest $P$ values when testing for associations with all variants. The beta distribution was then fitted to the null distribution of the 1000 smallest $P$ values. Similar to the approach taken for *cis*-meQTL analysis, the beta distribution was used to extrapolate empirical $P$ values from the nominal $P$ values, and the significance was set at FDR adjusted $P$ value < 0.05, corresponding to a nominal $P$ value threshold of $3.0\times {10}^{-10}$.

### Effect size correlation of the meQTLs

To quantify the consistency of mQTL effects between different populations/datasets, correlation coefficient of the top meQTLs was estimated. The analysis included three datasets/populations: the THCH data from the present study (Chinese, EAS), the Singapore iOmics data (Southeast Asians)^40^, and a meta-analysis of the Lothian Birth Cohort (LBC) and the Brisbane Systems Genetics Study (BSGS) data (EUR ancestry)^44^. When comparing the two datasets, we selected the top significant SNP-probe pairs in dataset A (as discovery) and matched the pairs in dataset B regardless of the significance (as replication). We standardized the effect size ($b$) and standard error (${SE}$) of top meQTLs using the equation $\hat{b}=z/\sqrt{2p(1-p)(n+{z}^{2})}$ and ${SE}=1/\sqrt{2p(1-p)(n+{z}^{2})}$, where $z=b/{SE}$, $p$ is MAF of the SNP, and *n* is the sample size^98^. This step guaranteed the comparison of the genetic effects on the same scale. We then used the ${r}_{b}$ method^45^ to estimate correlation of mQTL effects between two datasets considering both $\hat{b}$ and ${SE}$. The sampling variance of the estimated correlation coefficient was generated by the Jackknife approach.

### Functional enrichment analyses for the meQTLs

To characterize the functional regions enriched with meQTLs, we mapped the top meQTLs to 16 genomic elements in full-stack annotations predicted from large-scale datasets^89^. The QTLtools *fenrich* module^96^ was used for the enrichment analysis for the top *cis*-meQTLs, accounting for distance between the meQTL and DNAm probe. Permutation was performed 1000 times for each test. Fisher’s exact test was used to assess the difference between the observed and expected overlap frequencies. For the *trans*-meQTLs, for which it is not necessary to consider distance between the meQTL and DNAm probe, we used the frequency of the top *trans*-meQTL among the 1000 samples as the expectation. Fisher’s exact test was used to examine the difference. We set the significance level at the Bonferroni $P$ value threshold of $1.6\times {10}^{-3}$ (0.05/32).

### Colocalization analysis

EAS GWAS summary statistics for 32 common traits were obtained from inverse-variance weighted meta-analyses of Biobank Japan^46^ and the Korean Biobank^47^ data (Supplementary Table S25). We integrated the GWAS summary data with our meQTL summary data (*n* = 1448, EAS ancestry) and compared the results with those from integrating the GWAS data with the LBC + BSGS meQTL summary data^44^ (*n* = 1980, EUR ancestry). The colocalization analyses were performed using the *coloc.abf()* function in the *coloc* R package^99^ with the default parameters for each trait and for each *cis*-region of significant DNAm probes. We used a PP4 threshold of 0.8 to claim colocalization.

### Summary data-based Mendelian Randomization (SMR) analysis

We also used the SMR method^98^ to integrate the meQTLs from THCH or LBC + BSGS into GWAS using the same GWAS summary data as in the colocalization analysis above. The genotype data from the THCH cohort were used as the LD reference. We added the GoDMC meQTL dataset^43^ (*n* = 27,750) into the comparison. The default threshold of $5\times {10}^{-8}$ was used to select the top meQTL as the instrument for SMR analysis. Other parameters were set as follows: --diff-freq-prop 0.5 and --diff-freq 0.2. Significant SMR results were defined as ${P}_{{SMR}}$ < $0.05/m$ with *m* being the number of tested probes.

### Epigenetic clock analysis

We adopted the epigenetic age predictor established by Zhang et al.^50^ based on a BLUP model for the epigenetic clock analysis. We replicated the epigenetic age analysis using the Zhang et al. elastic net (EN) model^50^, the Horvath et al. multi-tissue-based model^48^, the Hannum et al. single-tissue-based model^51^, and the Levine et al. PhenoAge model^49^. The prediction accuracy was measured by the coefficient of determination (*R*^*2*^). The AAR was calculated as the difference between the predicted epigenetic age and the chronological age. The differences in AAR among the three groups were tested using the Kruskal-Wallis test. The pairwise differences were tested using the Wilcoxon signed-rank test. Outliers, which were defined as AARs outside of 1.5 times the interquartile range (IQR), were removed. To explore the risk factors associated with age acceleration for the highland subjects, a total of eight phenotype groups were categorized from the questionnaire and blood tests, namely, sun exposure, lifestyle, education, diet, arthrometry, blood cell components, metabolic factors, and endocrine factors (Supplementary Table S29). Each phenotype was normalized using RINT within ANs or NHs. After adjusting for age and age squared, we used analysis of variance (ANOVA) to test the association between AAR and a phenotype category. The statistical significance was set at a Bonferroni ${P}$ value threshold of $6.3\times {10}^{-3}$ (0.05/8).

## Supplementary information

Supplementary Note and figures

Supplementary Tables

## Data Availability

All the MWAS summary statistics are available in Supplementary Tables S3, S4, and DNAm beta values and phenotype information can be accessed upon request at https://ngdc.cncb.ac.cn/omix/release/OMIX003653. EWAS Catalog database: http://www.ewascatalog.org/download/; Annotation of Infinium DNA Methylation BeadChip probes: https://zwdzwd.github.io/InfiniumAnnotation; Epigenetic age coefficients: https://github.com/qzhang314/DNAm-based-age-predictor (Zhang et al.), https://horvath.genetics.ucla.edu/html/dnamage/ (Horvath et al.), Table S3 of Hannum et al., Supplement 2 of Levine et al.; ChinaMAP imputation server (http://www.mbiobank.com); Full-stack chromatin state annotations of the human genome: https://github.com/ernstlab/full_stack_ChromHMM_annotations; BioBank Japan GWAS summary statistics: https://humandbs.biosciencedbc.jp/en/hum0197-v3; KoGES GWAS summary statistics: https://koges.leelabsg.org/; McRae et al. meQTL summary data: https://yanglab.westlake.edu.cn/software/smr/#mQTLsummarydata; GoDMC meQTL summary data: http://godmc.org.uk/; Summary statistics of high-altitude genetic adaptation study: https://cnsgenomics.com/data/yang_et_al_2017_pnas.html.

## Code availability

We used the following software tools for data analysis. QTLtools: https://qtltools.github.io/qtltools/; SMR: https://yanglab.westlake.edu.cn/software/smr/; coloc: https://chr1swallace.github.io/coloc/; susieR: https://stephenslab.github.io/susieR/.

## References

[1] Körner, C. The use of ‘altitude’ in ecological research. *Trends Ecol. Evol.* **22**, 569–574 (2007). 17988759 10.1016/j.tree.2007.09.006

[2] Moore, L. G. Measuring high-altitude adaptation. *J. Appl. Physiol.* **123**, 1371–1385 (2017). 28860167 10.1152/japplphysiol.00321.2017 PMC5792094

[3] Zhang, X. L. et al. The earliest human occupation of the high-altitude Tibetan Plateau 40 thousand to 30 thousand years ago. *Science* **362**, 1049–1051 (2018). 30498126 10.1126/science.aat8824

[4] Yang, J. et al. Genetic signatures of high-altitude adaptation in Tibetans. *Proc. Natl. Acad. Sci. USA* **114**, 4189–4194 (2017). 28373541 10.1073/pnas.1617042114 PMC5402460

[5] Bennett, A., Sain, S. R., Vargas, E. & Moore, L. G. Evidence that parent-of-origin affects birth-weight reductions at high altitude. *Am. J. Hum. Biol.* **20**, 592–597 (2008). 18449923 10.1002/ajhb.20784

[6] Moore, L. G., Young, D., McCullough, R. E., Droma, T. & Zamudio, S. Tibetan protection from intrauterine growth restriction (IUGR) and reproductive loss at high altitude. *Am. J. Hum. Biol.* **13**, 635–644 (2001). 11505472 10.1002/ajhb.1102

[7] Chronic mountain sickness, optimal hemoglobin, and heart disease. *High Alt. Med. Biol*. **7**, 138–149 (2006). 10.1089/ham.2006.7.138 16764527

[8] Consensus statement on chronic and subacute high altitude diseases. *High Alt. Med. Biol.* **6**, 147–157 (2005). 10.1089/ham.2005.6.147 16060849

[9] Yi, X. et al. Sequencing of 50 human exomes reveals adaptation to high altitude. *Science* **329**, 75–78 (2010). 20595611 10.1126/science.1190371 PMC3711608

[10] Simonson, T. S., McClain, D. A., Jorde, L. B. & Prchal, J. T. Genetic determinants of Tibetan high-altitude adaptation. *Hum. Genet.* **131**, 527–533 (2012). 22068265 10.1007/s00439-011-1109-3

[11] Julian, C. G. & Moore, L. G. Human genetic adaptation to high altitude: evidence from the Andes. *Genes* **10**, 150 (2019). 30781443 10.3390/genes10020150 PMC6410003

[12] Simonson, T. S. et al. Genetic evidence for high-altitude adaptation in Tibet. *Science* **329**, 72–75 (2010). 20466884 10.1126/science.1189406

[13] Martin, E. M. & Fry, R. C. Environmental influences on the epigenome: exposure- associated DNA methylation in human populations. *Annu. Rev. Public Health* **39**, 309–333 (2018). 29328878 10.1146/annurev-publhealth-040617-014629

[14] Nanduri, J., Semenza, G. L. & Prabhakar, N. R. Epigenetic changes by DNA methylation in chronic and intermittent hypoxia. *Am. J. Physiol. Lung Cell Mol. Physiol.* **313**, L1096–L1100 (2017). 28839104 10.1152/ajplung.00325.2017 PMC5814703

[15] Lachance, G. et al. DNMT3a epigenetic program regulates the HIF-2α oxygen-sensing pathway and the cellular response to hypoxia. *Proc. Natl. Acad. Sci. USA* **111**, 7783 (2014). 24817692 10.1073/pnas.1322909111 PMC4040594

[16] Alkorta-Aranburu, G. et al. The genetic architecture of adaptations to high altitude in Ethiopia. *PLoS Genet.* **8**, e1003110 (2012). 23236293 10.1371/journal.pgen.1003110 PMC3516565

[17] Childebayeva, A. et al. Genome-wide epigenetic signatures of adaptive developmental plasticity in the Andes. *Genome Biol. Evol.* **13**, evaa239 (2021). 33185669 10.1093/gbe/evaa239 PMC7859850

[18] Childebayeva, A. et al. Genome-wide DNA methylation changes associated with high-altitude acclimatization during an Everest Base Camp trek. *Front. Physiol.* **12**, 660906–660906 (2021). 34262470 10.3389/fphys.2021.660906 PMC8273439

[19] Childebayeva, A. et al. DNA methylation changes are associated with an incremental ascent to high altitude. *Front. Genet.* **10**, 1062 (2019). 31737045 10.3389/fgene.2019.01062 PMC6828981

[20] Horvath, S. & Raj, K. DNA methylation-based biomarkers and the epigenetic clock theory of ageing. *Nat. Rev. Genet.* **19**, 371–384 (2018). 29643443 10.1038/s41576-018-0004-3

[21] Jylhävä, J., Pedersen, N. L. & Hägg, S. Biological age predictors. *EBioMedicine* **21**, 29–36 (2017). 28396265 10.1016/j.ebiom.2017.03.046 PMC5514388

[22] van Iterson, M., van Zwet, E. W. & Heijmans, B. T. & the, B.C. Controlling bias and inflation in epigenome- and transcriptome-wide association studies using the empirical null distribution. *Genome Biol.* **18**, 19 (2017). 28129774 10.1186/s13059-016-1131-9 PMC5273857

[23] Hop, P. J. et al. Genome-wide study of DNA methylation shows alterations in metabolic, inflammatory, and cholesterol pathways in ALS. *Sci. Transl. Med.* **14**, eabj0264 (2022). 35196023 10.1126/scitranslmed.abj0264 PMC10040186

[24] Gatev, E., Gladish, N., Mostafavi, S. & Kobor, M. S. CoMeBack: DNA methylation array data analysis for co-methylated regions. *Bioinformatics* **36**, 2675–2683 (2020). 31985744 10.1093/bioinformatics/btaa049

[25] Battram, T. et al. The EWAS Catalog: a database of epigenome-wide association studies. *Wellcome Open Res.* **7**, 41 (2022). 35592546 10.12688/wellcomeopenres.17598.1 PMC9096146

[26] Chen, D., Zhou, X., Zhu, Y., Zhu, T. & Wang, J. Comparison study on uterine and umbilical artery blood flow during pregnancy at high altitude and at low altitude. *Zhonghua Fu Chan Ke Za Zhi* **37**, 69–71 (2002). 11953065

[27] Beall, C. M. Two routes to functional adaptation: Tibetan and Andean high-altitude natives. *Proc. Natl. Acad. Sci. USA* **104**, 8655–8660 (2007). 17494744 10.1073/pnas.0701985104 PMC1876443

[28] Chambers, J. C. et al. Genome-wide association study identifies variants in TMPRSS6 associated with hemoglobin levels. *Nat. Genet.* **41**, 1170–1172 (2009). 19820698 10.1038/ng.462 PMC3178047

[29] Bollepalli, S., Korhonen, T., Kaprio, J., Anders, S. & Ollikainen, M. EpiSmokEr: a robust classifier to determine smoking status from DNA methylation data. *Epigenomics* **11**, 1469–1486 (2019). 31466478 10.2217/epi-2019-0206

[30] Semenza, G. L. Hypoxia. Cross talk between oxygen sensing and the cell cycle machinery. *Am. J. Physiol. Cell Physiol.* **301**, C550–C552 (2011). 21677261 10.1152/ajpcell.00176.2011 PMC3174572

[31] Goda, N. et al. Hypoxia-inducible factor 1alpha is essential for cell cycle arrest during hypoxia. *Mol. Cell. Biol.* **23**, 359–369 (2003). 12482987 10.1128/MCB.23.1.359-369.2003 PMC140666

[32] Manalo, D. J. et al. Transcriptional regulation of vascular endothelial cell responses to hypoxia by HIF-1. *Blood* **105**, 659–669 (2005). 15374877 10.1182/blood-2004-07-2958

[33] Martinez-Arguelles, D. B. & Papadopoulos, V. Epigenetic regulation of the expression of genes involved in steroid hormone biosynthesis and action. *Steroids* **75**, 467–476 (2010). 20156469 10.1016/j.steroids.2010.02.004 PMC2860648

[34] Berneburg, M. & Krutmann, J. Photoimmunology, DNA repair and photocarcinogenesis. *J. Photochem. Photobiol.* **54**, 87–93 (2000). 10.1016/s1011-1344(00)00024-5 10836536

[35] Martindale, J. L. & Holbrook, N. J. Cellular response to oxidative stress: signaling for suicide and survival. *J. Cell. Physiol.* **192**, 1–15 (2002). 12115731 10.1002/jcp.10119

[36] Islam, S. M. T., Won, J., Khan, M., Mannie, M. D. & Singh, I. Hypoxia-inducible factor-1 drives divergent immunomodulatory functions in the pathogenesis of autoimmune diseases. *Immunology* **164**, 31–42 (2021). 33813735 10.1111/imm.13335 PMC8358715

[37] McGettrick, A. F. & O’Neill, L. A. J. The Role of HIF in Immunity and Inflammation. *Cell Metab.* **32**, 524–536 (2020). 32853548 10.1016/j.cmet.2020.08.002

[38] Scholz, C. C. & Taylor, C. T. Targeting the HIF pathway in inflammation and immunity. *Curr. Opin. Pharmacol.* **13**, 646–653 (2013). 23660374 10.1016/j.coph.2013.04.009

[39] Xin, J. -W. et al. Transcriptome profiles revealed the mechanisms underlying the adaptation of yak to high-altitude environments. *Sci. Rep.* **9**, 7558 (2019). 31101838 10.1038/s41598-019-43773-8 PMC6525198

[40] Kassam, I. et al. Genome-wide identification of cis DNA methylation quantitative trait loci in three Southeast Asian Populations. *Hum. Mol. Genet.* **30**, 603–618 (2021). 33547791 10.1093/hmg/ddab038

[41] Villicaña, S. & Bell, J. T. Genetic impacts on DNA methylation: research findings and future perspectives. *Genome Biol.* **22**, 127 (2021). 33931130 10.1186/s13059-021-02347-6 PMC8086086

[42] Huan, T. et al. Genome-wide identification of DNA methylation QTLs in whole blood highlights pathways for cardiovascular disease. *Nat. Commun.* **10**, 4267 (2019). 10.1038/s41467-019-12228-z PMC6753136 31537805

[43] Min, J. L. et al. Genomic and phenotypic insights from an atlas of genetic effects on DNA methylation. *Nat. Genet.* **53**, 1311–1321 (2021). 34493871 10.1038/s41588-021-00923-x PMC7612069

[44] McRae, A. F. et al. Identification of 55,000 Replicated DNA Methylation QTL. *Sci. Rep.* **8**, 17605 (2018). 30514905 10.1038/s41598-018-35871-w PMC6279736

[45] Qi, T., et al. Identifying gene targets for brain-related traits using transcriptomic and methylomic data from blood. *Nat. Commun.* **9**, 2282 (2018). 29891976 10.1038/s41467-018-04558-1 PMC5995828

[46] Sakaue, S. et al. A global atlas of genetic associations of 220 deep phenotypes. *medRxiv*, 10.1101/2020.10.23.20213652 (2021).

[47] Nam, K., Kim, J. & Lee, S. Genome-wide study on 72,298 Korean individuals in Korean biobank data for 76 traits identifies hundreds of novel loci. *medRxiv*, 10.1101/2022.02.23.22271389 (2022). 10.1016/j.xgen.2022.100189 PMC9903843 36777999

[48] Horvath, S. DNA methylation age of human tissues and cell types. *Genome Biol.* **14**, 3156 (2013). 10.1186/gb-2013-14-10-r115 PMC4015143 24138928

[49] Levine, M. E. et al. An epigenetic biomarker of aging for lifespan and healthspan. *Aging* **10**, 573–591 (2018). 29676998 10.18632/aging.101414 PMC5940111

[50] Zhang, Q. et al. Improved precision of epigenetic clock estimates across tissues and its implication for biological ageing. *Genome Med.* **11**, 54 (2019). 10.1186/s13073-019-0667-1 PMC6708158 31443728

[51] Hannum, G. et al. Genome-wide methylation profiles reveal quantitative views of human aging rates. *Mol. Cell* **49**, 359–367 (2013). 23177740 10.1016/j.molcel.2012.10.016 PMC3780611

[52] ANGERS, B., CASTONGUAY, E. & MASSICOTTE, R. Environmentally induced phenotypes and DNA methylation: how to deal with unpredictable conditions until the next generation and after. *Mol. Ecol.* **19**, 1283–1295 (2010). 20298470 10.1111/j.1365-294X.2010.04580.x

[53] Anderson, O. S., Sant, K. E. & Dolinoy, D. C. Nutrition and epigenetics: an interplay of dietary methyl donors, one-carbon metabolism and DNA methylation. *J. Nutr. Biochem.* **23**, 853–859 (2012). 22749138 10.1016/j.jnutbio.2012.03.003 PMC3405985

[54] Allison, J., Kaliszewska, A., Uceda, S., Reiriz, M. & Arias, N. Targeting DNA methylation in the adult brain through diet. *Nutrients* **13**, 3979 (2021). 34836233 10.3390/nu13113979 PMC8618930

[55] Mallet, R. T. et al. Molecular mechanisms of high-altitude acclimatization. *Int. J. Mol. Sci.* **24**, 1698 (2023). 36675214 10.3390/ijms24021698 PMC9866500

[56] Wu, T. & Kayser, B. High altitude adaptation in Tibetans. *High. Alt. Med. Biol.* **7**, 193–208 (2006). 16978132 10.1089/ham.2006.7.193

[57] Bianba, B. et al. Anthropometric measures of 9- to 10-year-old native Tibetan children living at 3700 and 4300 m above sea level and Han Chinese living at 3700 m. *Medicine* **94**, e1516 (2015). 26496254 10.1097/MD.0000000000001516 PMC4620755

[58] Dang, S., Yan, H. & Yamamoto, S. High altitude and early childhood growth retardation: new evidence from Tibet. *Eur. J. Clin. Nutr.* **62**, 342–348 (2008). 17342161 10.1038/sj.ejcn.1602711

[59] Weitz, C. A., Garruto, R. M., Chin, C. T. & Liu, J. C. Morphological growth and thorax dimensions among Tibetan compared to Han children, adolescents and young adults born and raised at high altitude. *Ann. Hum. Biol.* **31**, 292–310 (2004). 15204346 10.1080/0301446042000196316

[60] Wu, D. et al. How placenta promotes the successful reproduction in high-altitude populations: a transcriptome comparison between adaptation and acclimatization. *Mol. Biol. Evol.* **39**, msac120 (2022). 35642306 10.1093/molbev/msac120 PMC9206416

[61] White, W. M. et al. Normal early pregnancy: a transient state of epigenetic change favoring hypomethylation. *Epigenetics* **7**, 729–734 (2012). 22647708 10.4161/epi.20388 PMC3414393

[62] Barrès, R. et al. Acute exercise remodels promoter methylation in human skeletal muscle. *Cell Metab.* **15**, 405–411 (2012). 22405075 10.1016/j.cmet.2012.01.001

[63] Shahrzad, S., Bertrand, K., Minhas, K. & Coomber, B. Induction of DNA hypomethylation by tumor hypoxia. *Epigenetics* **2**, 119–125 (2007). 17965619 10.4161/epi.2.2.4613

[64] Eden, A., Gaudet, F., Waghmare, A. & Jaenisch, R. Chromosomal instability and tumors promoted by DNA hypomethylation. *Science* **300**, 455–455 (2003). 12702868 10.1126/science.1083557

[65] Yauk, C. et al. Germ-line mutations, DNA damage, and global hypermethylation in mice exposed to particulate air pollution in an urban/industrial location. *Proc. Natl. Acad. Sci. USA* **105**, 605–610 (2008). 10.1073/pnas.0705896105 PMC2206583 18195365

[66] Lind, L. et al. Global DNA hypermethylation is associated with high serum levels of persistent organic pollutants in an elderly population. *Environ. Int.* **59**, 456–461 (2013). 23933504 10.1016/j.envint.2013.07.008

[67] Liu, H. et al. Epigenomic and transcriptomic analyses define core cell types, genes and targetable mechanisms for kidney disease. *Nat. Genet.* **54**, 950–962 (2022). 35710981 10.1038/s41588-022-01097-w PMC11626562

[68] Taylor, D. L. et al. Integrative analysis of gene expression, DNA methylation, physiological traits, and genetic variation in human skeletal muscle. *Proc. Natl. Acad. Sci. USA* **116**, 10883–10888 (2019). 31076557 10.1073/pnas.1814263116 PMC6561151

[69] Bono, H. & Hirota, K. Meta-analysis of hypoxic transcriptomes from public databases. *Biomedicines* **8**, 10 (2020). 31936636 10.3390/biomedicines8010010 PMC7168238

[70] Miller, S. et al. Maternal and neonatal outcomes of hospital vaginal deliveries in Tibet. *Int. J. Gynaecol. Obstet.* **98**, 217–221 (2007). 17481630 10.1016/j.ijgo.2007.03.033 PMC2194809

[71] Niermeyer, S., Andrade, M. M., Vargas, E. & Moore, L. G. Neonatal oxygenation, pulmonary hypertension, and evolutionary adaptation to high altitude (2013 Grover Conference series). *Pulm. Circ.* **5**, 48–62 (2015). 25992270 10.1086/679719 PMC4405714

[72] Brutsaert, T. D. Do high-altitude natives have enhanced exercise performance at altitude? *Appl. Physiol. Nutr. Metab.* **33**, 582–592 (2008). 10.1139/H08-009 18461115

[73] Pomatto, L. C. D. & Davies, K. J. A. The role of declining adaptive homeostasis in ageing. *J. Physiol.* **595**, 7275–7309 (2017). 29028112 10.1113/JP275072 PMC5730851

[74] Pomatto, L. C. D. & Davies, K. J. A. Adaptive homeostasis and the free radical theory of ageing. *Free Radic. Biol. Med.* **124**, 420–430 (2018). 29960100 10.1016/j.freeradbiomed.2018.06.016 PMC6098721

[75] El Assar, M., Angulo, J. & Rodríguez-Mañas, L. Oxidative stress and vascular inflammation in aging. *Free Radic. Biol. Med.* **65**, 380–401 (2013). 23851032 10.1016/j.freeradbiomed.2013.07.003

[76] Wu, J., Xia, S., Kalionis, B., Wan, W. & Sun, T. The role of oxidative stress and inflammation in cardiovascular aging. *BioMed. Res. Int.* **2014**, 615312 (2014). 25143940 10.1155/2014/615312 PMC4131065

[77] Franceschi, C. et al. Inflamm-aging: an evolutionary perspective on immunosenescence. *Ann. N. Y. Acad. Sci.* **908**, 244–254 (2000). 10911963 10.1111/j.1749-6632.2000.tb06651.x

[78] Navarrete-Opazo, A. & Mitchell, G. S. Therapeutic potential of intermittent hypoxia: a matter of dose. *Am. J. Physiol. Regul. Integr. Comp. Physiol.* **307**, R1181–R1197 (2014). 25231353 10.1152/ajpregu.00208.2014 PMC4315448

[79] Lu, X. J. et al. Hippocampal spine-associated Rap-specific GTPase-activating protein induces enhancement of learning and memory in postnatally hypoxia-exposed mice. *Neuroscience* **162**, 404–414 (2009). 19442707 10.1016/j.neuroscience.2009.05.011 PMC3243647

[80] Row, B. W., Kheirandish, L., Cheng, Y., Rowell, P. P. & Gozal, D. Impaired spatial working memory and altered choline acetyltransferase (CHAT) immunoreactivity and nicotinic receptor binding in rats exposed to intermittent hypoxia during sleep. *Behav. Brain Res.* **177**, 308–314 (2007). 17218023 10.1016/j.bbr.2006.11.028 PMC1847578

[81] Pidsley, R. et al. Critical evaluation of the Illumina MethylationEPIC BeadChip microarray for whole-genome DNA methylation profiling. *Genome Biol.* **17**, 208 (2016). 27717381 10.1186/s13059-016-1066-1 PMC5055731

[82] Simpson, J. T. et al. Detecting DNA cytosine methylation using nanopore sequencing. *Nat. Methods* **14**, 407–410 (2017). 28218898 10.1038/nmeth.4184

[83] Frisancho, A. R., Martinez, C., Velasquez, T., Sanchez, J. & Montoye, H. Influence of developmental adaptation on aerobic capacity at high altitude. *J. Appl. Physiol.* **34**, 176–180 (1973). 4686351 10.1152/jappl.1973.34.2.176

[84] Min, J. L., Hemani, G., Davey Smith, G., Relton, C. & Suderman, M. Meffil: efficient normalization and analysis of very large DNA methylation datasets. *Bioinformatics* **34**, 3983–3989 (2018). 29931280 10.1093/bioinformatics/bty476 PMC6247925

[85] Fortin, J. -P. et al. Functional normalization of 450k methylation array data improves replication in large cancer studies. *Genome Biol.* **15**, 503 (2014). 25599564 10.1186/s13059-014-0503-2 PMC4283580

[86] Du, P. et al. Comparison of Beta-value and M-value methods for quantifying methylation levels by microarray analysis. *BMC Bioinforma.* **11**, 587–587 (2010). 10.1186/1471-2105-11-587 PMC3012676 21118553

[87] Houseman, E. A. et al. DNA methylation arrays as surrogate measures of cell mixture distribution. *BMC Bioinformatics* **13**, 86–86 (2012). 10.1186/1471-2105-13-86 PMC3532182 22568884

[88] Yang, J. et al. Genomic inflation factors under polygenic inheritance. *Eur. J. Hum. Genet.* **19**, 807–812 (2011). 21407268 10.1038/ejhg.2011.39 PMC3137506

[89] Vu, H. & Ernst, J. Universal annotation of the human genome through integration of over a thousand epigenomic datasets. *Genome Biol.* **23**, 9 (2022). 34991667 10.1186/s13059-021-02572-z PMC8734071

[90] Ren, X. & Kuan, P. F. methylGSA: a Bioconductor package and Shiny app for DNA methylation data length bias adjustment in gene set testing. *Bioinformatics* **35**, 1958–1959 (2019). 30346483 10.1093/bioinformatics/bty892

[91] Phipson, B., Maksimovic, J. & Oshlack, A. missMethyl: an R package for analyzing data from Illumina’s HumanMethylation450 platform. *Bioinformatics* **32**, 286–288 (2016). 26424855 10.1093/bioinformatics/btv560

[92] Li, L. et al. The ChinaMAP reference panel for the accurate genotype imputation in Chinese populations. *Cell Res.* **31**, 1308–1310 (2021). 34489580 10.1038/s41422-021-00564-z PMC8648815

[93] Yang, J., Lee, S. H., Goddard, M. E. & Visscher, P. M. GCTA: a tool for genome-wide complex trait analysis. *Am. J. Hum. Genet.* **88**, 76–82 (2011). 21167468 10.1016/j.ajhg.2010.11.011 PMC3014363

[94] Loh, P. R. et al. Reference-based phasing using the Haplotype Reference Consortium panel. *Nat. Genet.* **48**, 1443–1448 (2016). 27694958 10.1038/ng.3679 PMC5096458

[95] Durbin, R. Efficient haplotype matching and storage using the positional Burrows-Wheeler transform (PBWT). *Bioinformatics* **30**, 1266–1272 (2014). 24413527 10.1093/bioinformatics/btu014 PMC3998136

[96] Delaneau, O. et al. A complete tool set for molecular QTL discovery and analysis. *Nat. Commun.* **8**, 15452 (2017). 28516912 10.1038/ncomms15452 PMC5454369

[97] Storey, J. D. & Tibshirani, R. Statistical significance for genomewide studies. *Proc. Natl. Acad. Sci. USA* **100**, 9440 (2003). 10.1073/pnas.1530509100 PMC170937 12883005

[98] Zhu, Z. et al. Integration of summary data from GWAS and eQTL studies predicts complex trait gene targets. *Nat. Genet.* **48**, 481–487 (2016). 27019110 10.1038/ng.3538

[99] Giambartolomei, C. et al. Bayesian Test for Colocalisation between Pairs of Genetic Association Studies Using Summary Statistics. *PLOS Genet.* **10**, e1004383 (2014). 24830394 10.1371/journal.pgen.1004383 PMC4022491

