# Supplementary material for: Distinct methylomic signatures of high-altitude acclimatization and adaptation in the Tibetan Plateau
Source: Cell Discov. 2025 May 6;11:45. doi: 10.1038/s41421-025-00795-z (PMC12056056; doi:10.1038/s41421-025-00795-z)
Supplement: Supplementary file 1 — Supplementary Note and figures [file 41421_2025_795_MOESM1_ESM.docx]

Distinct methylomic signatures of high-altitude acclimatization and adaptation in the Tibetan Plateau

Cheng *et al*.

Table of Contents

[Supplementary Note 3](#_Toc184149273)

[Section 1: Simulations to evaluate MWAS methods. 3](#_Toc184149274)

[1.1 Simulation design without confounders 3](#_Toc184149275)

[1.2 Evaluation of MWAS methods 3](#_Toc184149276)

[1.3 The unknown covariates in the linear model 4](#_Toc184149277)

[1.4 Simulation design with confounders 5](#_Toc184149278)

[1.5 Evaluation of the adjustment for confounding effects 5](#_Toc184149279)

[Section 2: Overlap of the high-altitude DMSs with MWAS databases or published work 7](#_Toc184149280)

[2.1 Overlap of the STA DMSs with EWAS databases or published work 7](#_Toc184149281)

[2.2 Overlap of the LTA DMSs with EWAS databases or published work 7](#_Toc184149282)

[Section 3: Sensitivity analyses for the MWAS results 7](#_Toc184149283)

[3.1 MWAS comparing between NHs and NLs 7](#_Toc184149284)

[3.2 MWAS within female samples only 8](#_Toc184149285)

[3.3 MWAS conditioning on predicted smoking status 8](#_Toc184149286)

[3.4 The consistency of MWAS results among experimental batches 9](#_Toc184149287)

[Section 4: The differences in estimated cell type components between Tibetans and Han 9](#_Toc184149288)

[Section 5: Elucidation of gene-set enrichment analyses for DMSs 9](#_Toc184149289)

[5.1 Evidence from literature for the enriched pathways of the STA DMSs 9](#_Toc184149290)

[5.2 Evidence from literature for the enriched pathways of the LTA DMSs 10](#_Toc184149291)

[5.3 The dissection of enrichment analyses for hypermethylated and hypomethylated DMSs 12](#_Toc184149292)

[Section 6: Additional characteristics of the THCH meQTLs 13](#_Toc184149293)

[Section 7: The effectiveness of utilizing the THCH meQTLs to identify functional elements associated with complex traits in samples of EAS ancestry 15](#_Toc184149294)

[Section 8: Sensitivity analyses for the AAR difference test 16](#_Toc184149295)

[Section 9: Replication analyses using the commonly used epigenetic age predictors 17](#_Toc184149296)

[Section 10: Genome-wide association study for AAR 18](#_Toc184149297)

[Section 11: EAS-meQTL results using different imputation reference panels 18](#_Toc184149298)

[Supplementary Figures 19](#_Toc184149299)

[Supplementary Figure S1: The distribution of highland living years among acclimatized newcomers. 19](#_Toc184149300)

[Supplementary Figure S2: Simulation results for MWAS method evaluation. 20](#_Toc184149301)

[Supplementary Figure S3: Simulation results of the Bacon method. 21](#_Toc184149302)

[Supplementary Figure S4: The simulation results of adding the confounder as a covariate. 22](#_Toc184149303)

[Supplementary Figure S5: Manhattan plots of the MWAS sensitivity analyses. 23](#_Toc184149304)

[Supplementary Figure S6: Manhattan plots of MWAS results within experimental batches. 24](#_Toc184149305)

[Supplementary Figure S7: Boxplots of cell type proportion estimated from DNAm for the Tibetan and Han Chinese. 25](#_Toc184149306)

[Supplementary Figure S8: Manhattan plot of MWAS results comparing NHs and NLs. 26](#_Toc184149307)

[Supplementary Figure S9: Scatter plot of the coefficient of determination against the distance between DNAm probes and the corresponding top *cis*-meQTLs. 27](#_Toc184149308)

[Supplementary Figure S10: Distribution of pleiotropic variants and credible set(s) of *cis*-meQTLs. 28](#_Toc184149309)

[Supplementary Figure S11: Genome-wide scan of three typical meQTL examples. 31](#_Toc184149310)

[Supplementary Figure S12: Scatter plots of pairwise effect sizes of meQTLs from different groups or cohorts. 32](#_Toc184149311)

[Supplementary Figure S13: Bar plot showing the number of prioritised DNAm probes associated with 32 common traits using SMR and HEIDI. 33](#_Toc184149312)

[Supplementary Figure S14: Examples of population-specific meQTLs. 34](#_Toc184149313)

[Supplementary Figure S15: Examples of EAS-specific DNAm probes associated with traits prioritized by SMR analysis. 35](#_Toc184149314)

[Supplementary Figure S16: Predictive performance of epigenetic age predictors and age acceleration residual (AAR) tests. 37](#_Toc184149315)

[Supplementary Figure S17: Manhattan plot of GWAS results for age acceleration residual (AAR) in the THCH cohort. 39](#_Toc184149316)

[Supplementary Figure S18: MWAS analyses for age acceleration residual. 40](#_Toc184149317)

[Supplementary Figure S19: MWAS analyses for eight phenotype categories, i.e. sun exposure, lifestyle, education, diet, anthropometry, blood cell components, metabolic traits, and endocrine factors. 43](#_Toc184149318)

[Supplementary Figure S20: Scatter plot of genetic principal component analysis. 44](#_Toc184149319)

[References 45](#_Toc184149320)

# **Supplementary Note**

## **Section 1:** Simulations to evaluate MWAS methods.

### 1.1 Simulation design without confounders

To simulate the scenario in our real data where changes in DNAm are caused by environmental exposures, we performed a simulation study under the assumption that a phenotype causes DNAm changes. We evaluated the statistical power and false positives of different MWAS methods under various settings, including the percentage of probes affected by the phenotype, the effect sizes, and the number of covariates (e.g., principal components or surrogate variables). The procedure consists of four steps:

1) Extracting real DNAm information of chromosome 20, comprising 1,622 samples and 7,693 probes, from the THCH data.

2) Simulating a random phenotype following a standard normal distribution.

3) Generating new methylation data by randomly selecting a certain proportion of DNAm probes as those that can be affected by the phenotype. For the non-affected DNAm probes, they are kept intact. For each affected DNAm probe, a term of an effect size times the phenotype, and an error term are added to the raw data. The effect sizes of all affected probes follow a normal distribution $\beta\sim N(0,\sigma^{2})$.

4) Associating the simulated phenotype with each of the DNA probes using different MWAS methods. The inflation factor, statistical power, and false positives are recorded for each method and simulation.

### 1.2 Evaluation of MWAS methods

We performed MWAS analyses on the simulated data using a linear regression-based method and two linear mixed model (LMM)-based methods, MOA and MOMENT, implemented by the OSCA software^1^. We varied the proportion of affected DNAm probes from 5% to 50% with an interval of 5% and repeated each scenario 10 times. Results showed that all three methods exhibited almost no false positive associations under the current simulation setting. In this case, the inflation factor, which is expected to be larger than one in the presence of true associations, can be used to quantify the statistical power. We observed that the inflation factor increased as the proportion of affected probes increased for the linear regression-based method, while the inflation factors of MOA and MOMENT remained around or below one (Supplementary Fig. S2a), suggesting a substantial loss of power for MOA and MOMENT.

We then fixed the proportion of affected DNAm probes at 21% and varied the variance of the effect size distribution (i.e., $\sigma^{2}$). MOA and MOMENT again exhibited lower inflation factors and statistical power than linear regression regardless of the variance of the effect size distribution (Supplementary Fig. S2b-c). In addition, MOA and MOMENT encountered convergence problems and produced output errors in some cases, particularly when the variance of the distribution was large. Based on these findings, we concluded that, under the given settings, the LMM-based methods, MOA and MOMENT, were overly conservative, especially when the proportion of affected probes was high. Therefore, we opted to use the linear regression-based method for real data analysis.

### 1.3 The unknown covariates in the linear model

In MWAS analyses, principal components (PCs) and surrogate variables (SVs) are often used to adjust for cell mixtures and other unknown confounding factors. To assess the necessity of including PCs/SVs in the linear regression-based method, we examined the impact of fitting PCs/SVs on inflation factor and false positive rate by simulations. We kept the proportion of affected probes and variance of the effect size distribution fixed in the simulations. We observed a sharp increase in the inflation factor and false positive rate when adding around 5-15 PCs as covariates, followed by a decrease to around 1 (Supplementary Fig. S2d). Adding different number of SVs had less impact on the inflation factor, but the inflation factors varied in each replication (Supplementary Fig. S2e). Based on the results, it was evident that the addition of unknown covariates was underdetermined, and thus, further simulations were necessary to validate the results with confounding effects.

### 1.4 Simulation design with confounders

To address potential confounding effects, we conducted simulations to investigate scenario where changes in DNAm are caused by environmental exposures with confounding effects. These simulations utilized real DNAm data and a skewed distribution of true effect sizes. The following steps were taken:

1) Extracting real DNAm information of 1,622 samples and 100k probes from the THCH data.

2) Simulating a binary phenotype with equal proportions of cases and controls selected randomly.

3) Generating effect sizes of the simulated phenotype on a certain proportion of DNAm probes using a gamma distribution and adjusting them to match the scale the effect sizes observed from the real data analysis.

4) Simulating a confounder associated with the phenotype. The effect sizes of the confounder on all probes (or a certain proportion of probes) follow a normal distribution $\beta\sim N(0,\sigma^{2})$.

5) Simulating DNAm levels by combining the phenotype effect, confounder effect, raw methylation level, and an error term.

6) Associating the simulated phenotype with each of the DNA probes using a linear regression-based method with different strategies to adjust for confounding effects. The inflation factor and false positives are recorded before and after adjustment.

### 1.5 Evaluation of the adjustment for confounding effects

To explore the necessity of including PCs as covariates in the linear regression-based MWAS model, we assessed the performance of the model by adding varying numbers of PCs using the data simulated above with a confounder. Consistent with the simulation results without a confounder, the addition of PCs induced many false positives, which were potentially due to collider bias. Moreover, adding PCs substantially decreased power, as many PCs were significantly associated with phenotypes (e.g., R^2^=0.32 between PC4 and the LTA phenotype). Therefore, we did not include PCs as covariates in real data analysis.

We then considered using the Bayesian method called *bacon* method to correct for uncontrolled confounding and bias in test statistics^2^. The *bacon* method estimates the empirical null distribution by fitting a three-component normal mixture using a Gibbs sampling algorithm. One component reflects the empirical null, while the two other components represent positive/negative true associations. We evaluated the method by varying a series of parameters, such as the percentage of affected probes, confounding effect size, and the percentage of confounder-affected probes. In most settings, *bacon* controlled the false positives at a relatively low level (false positive rates from 0 to 0.07) while sacrificing a certain level of statistical power (Supplementary Table S2).

For instance, almost no false positives were detected when the simulated confounder affected all probes, and the effect sizes followed a normal distribution (Supplementary Fig. S3**)**. When the confounding effects were small to moderate, the performance of *bacon* in controlling increased with the increasing sizes of the confounding effects and the proportion of confounder-affected probes. However, we should note that when the simulated confounder affected only a small number of probes with large effects, *bacon* method did not work for adjustment. In such cases, including the confounders (such as age and sex) as covariates can effectively resolve the issue (Supplementary Fig. S4). Given that we have already included covariates for sex, age, batch, and estimated cell type components, it is very unlikely that our conclusions would be substantially affected by known confounding factors.

In our real data application, the inflation factors ($\lambda$) decreased from 1.69 to 1.04 for STA and from 2.65 to 1.35 for LTA after *bacon* correction. The Geweke diagnostic indicated satisfactory convergence for STA and LTA (Z-scores for STA: p.0=0.75, mu.0=-1.21, sigma.0=0.62; Z-scores for LTA: p.0=0.82, mu.0=0.30, sigma.0=0.73). Simulation results, which exhibited similar patterns and estimates as the real data, showed limited false positives. Therefore, we suggest that *bacon* adjustment provided reliable associations in our real data analysis.

## **Section 2:** Overlap of the high-altitude DMSs with MWAS databases or published work

### 2.1 Overlap of the STA DMSs with EWAS databases or published work

The short-term DNAm signals did not overlap with DMSs from the Everest Base Camp Trek study^3^. It could be due to the different technical assays being used (Illumina MethylationEPIC chip), the small sample size (15 participants), and the differences in ancestry (European population). According to the EWAS Catalog database, 90 out of 93 of DNAm signals were significant in other traits. The top overlapping traits are age, tissue type, rheumatoid arthritis, HIV infection, smoking, and alcohol consumption (Supplementary Table S19).

### 2.2 Overlap of the LTA DMSs with EWAS databases or published work

There is no overlap in DNAm signals between our THCH study and the Ethiopian study with 34 participants^4^. According to the EWAS Catalog database, 3,975 out of the 4,070 of LTA DMSs were significant in other traits, with the top overlapping traits being age, tissue type, sex, gestational age, smoking, and alcohol consumption (Supplementary Table S20). It is noteworthy that a large proportion of the DMSs overlapped with phenotypes related to survival rate, such as 1,195 probes associated with gestational age, 65 probes associated with birth weight, 84 probes associated with hypertensive disorders of pregnancy, and 25 probes associated with preeclampsia. The enrichment test showed significant enrichment for survive-relevant phenotypes: gestational age (Fisher's one-side Exact Test: odds ratio (OR) = 2.67, $P$value = $5.9\times{10}^{-23}$), and birth weight (OR = 2.58, $P$value = $6.0\times{10}^{-6}$. Further studies are required to investigate the underlying mechanisms of the overlapping DMSs.

## **Section 3:** Sensitivity analyses for the MWAS results

### 3.1 MWAS comparing between NHs and NLs

Upon comparing 687 NHs (as the target group) and 462 NLs (as the control group), *bacon* adjustment failed to achieve a satisfactory convergence under the default parameters even with longer burn-in and iteration (Geweke diagnostic Z-scores: p.0=0.27, mu.0=2.46, sigma.0=2.23). This is likely due to more complicated polymethylomic structure. Therefore, we modified the trimming parameter from 0.999 to 0.99, which led to a converged result, identifying 1,930 DMSs (Supplementary Fig. S8; the inflation factor was 2.54). Among these, 1,421 DMSs overlapped with LTA signals and 31 DMSs overlapped with STA signals. The KEGG enrichment analysis highlighted autoimmune diseases, neuroactive ligand-receptor interaction and the calcium signalling pathway. The top enriched GO term was mainly related to morphogenesis and development. Overall, the pattern was closely resembled the LTA MWAS result.

### 3.2 MWAS within female samples only

We performed MWAS analyses on female samples and found that the results of both STA and LTA were consistent with the original findings (Supplementary Fig. S5a-b). In detail, we replicated 76.3% (71 out of 93) of the probes for STA and 90.3% (3,676 out of 4,070) of the probes for LTA at the *Bacon* adjusted *P* threshold of $1.0\times{10}^{-7}$. Before the *Bacon* adjustment, we replicated 95.7% (89 out of 93) of the probes for STA and 100% (4,070 out of 4,070) of the probes for LTA at the nominal *P* threshold of $1.0\times{10}^{-7}$.

### 3.3 MWAS conditioning on predicted smoking status

To account for the potential impact of smoking on our study results, we included a covariate of predicted smoking status, which was obtained using the EpiSmokEr R package^5^. The prediction revealed that there were 1,283 participants who had never smoked, 109 former smokers, and 56 current smokers in our sample. Our questionnaire data showed that the smoking rate among participants in the TP group was consistently low, with only 1 out of 119 ANs and 3 out of 93 NHs reporting having ever smoked. After adjusting for predicted smoking status, we found that the MWAS results for both STA and LTA were consistent with our original findings (Supplementary Fig. S5c-d). In detail, we replicated 100% (93 out of 93) of the probes for STA and 84.2% (3,425 out of 4,070) of the probes for LTA at the *Bacon* adjusted *P* threshold of $1.0\times{10}^{-7}$. Before the *Bacon* adjustment, we replicated 100% (93 out of 93) of the probes for STA and 100% (4,070 out of 4,070) of the probes for LTA at the *P* threshold of $1.0\times{10}^{-7}$.

### 3.4 The consistency of MWAS results among experimental batches

To investigate potential batch effects, we performed MWAS for LTA in batches 1 and 3, and for NHs versus NLs in batches 2 and 3, respectively (Supplementary Fig. S6). For LTA, the Pearson correlation of effect sizes of all DNAm probes between batches 1 and 3 was 0.733 [95% CI: 0.732-0.735]. For NHs versus NLs, the Pearson correlation between batches 2 and 3 was 0.760 [95% CI: 0.759-0.762]. Using the $r_{b}$ method, the estimated correlation of effect size for LTA between batches 1 and 3 was 0.968 (SE 0.002) for those with $P$ values < 0.05 in one batch, and the estimate was 0.942 (SE 0.001) for NHs versus NLs between batches 2 and 3. These results suggest a high level of consistency among the experimental batches for the MWAS results.

## **Section 4:** The differences in estimated cell type components between Tibetans and Han

In the MWAS analysis, the test model incorporated estimated cell composition as covariates. This allowed for the examination of differences in cell composition between Tibetan and Han Chinese populations (Supplementary Fig. S7). Our findings revealed a significantly lower proportion of B cells, CD4^+^ cells, and Natural Killer (NK) cells for NHs, while there was a higher proportion of monocytes and neutrophils when compared to NLs. ANs also exhibited a similar trend, with lower proportions of the B cells, CD4^+^ cells, and NK cells in NHs, along with higher proportions of monocytes and Neutrophils compared to NLs, albeit with lower statistical significance. It is worth noting that previous investigations into cell components of high-altitude populations are scarce. A relevant study suggested that NHs have lower B cells and CD4^+^ cells, but with increased NK cells using flow cytometry^6^. Another study exhibited a significantly decrease in CD4^+^ cells and increase in NK cells after short-term high-altitude exposure using flow cytometry^7,8^. However, NK cytotoxic activity was not affected by the exposure^7^.

## **Section 5:** Elucidation of gene-set enrichment analyses for DMSs

### 5.1 Evidence from literature for the enriched pathways of the STA DMSs

In the gene-set enrichment analysis (GSEA) of the STA DMSs, the cell cycle pathway category was highlighted (Supplementary Tables S17-24). It is well-recognized that there is an intricate relationship between hypoxia and cell cycle at both transcriptional and post-transcriptional levels^9,10^. Previous observations have shown that hypoxia inhibits cell proliferation in most cell types to reduce oxygen consumption^11,12^, while stimulating specialized cell types, such as vascular endothelial cells, to maintain oxygen homeostasis^13^. Relevantly, DMSs of STA were enriched in cancer-related pathways, including breast cancer, microRNAs in cancer, gastric cancer, and hepatocellular carcinoma, shedding light on the relationship between hypoxia and tumor growth^14,15^.

Furthermore, significant enrichment observed in biological processes related to DNA repair, cellular response to stress, and steroid biosynthesis suggests that these pathways may play an important role in responding to short-term exposure at high altitude. Hypoxia and UV radiation at high altitude are known to induce oxidative stress^16-18^, which can lead to DNA damage and cellular stress. Accordingly, the processes of cellular response to stress and DNA repair are regulated to maintain homeostasis through various signaling pathways and transcriptional regulators. In terms of steroid biosynthesis, different types of steroid hormones might exert functions in short-term acclimatization. 1) Glucocorticoid can be secreted in response to stress through hypothalamic-pituitary-adrenal axis^19-21^. Previous studies have shown that the cortisol concentrations increase ~32% from baseline during acute exposure to high altitude^22,23^. 2) Testosterone can regulate erythrocyte production, which is important to maintain oxygen homeostasis. Evidence has indicated that testosterone levels would rapidly increase after arrival at high altitude as well^22^. 3) Additional evidence has shown that menopause might be a risk factor for the development of chronic mountain sickness (CMS)^24,25^ and presented the association between progesterone and CMS biomarkers^26^. Overall, these enriched pathways exhibited functional relevance and importance in short-term high-altitude acclimatization.

### 5.2 Evidence from literature for the enriched pathways of the LTA DMSs

In the GSEA of the LTA DMSs, one of the highlighted pathways is the calcium signalling pathway, which is involved in various processes such as fertilization, proliferation, development, learning and memory, contraction, and secretion^27^ (Supplementary Tables S9-16). The literature has suggested that hypoxia can cause an increase in intracellular concentration of Ca^2+^, leading to pulmonary vasoconstriction^28,29^. Candidate genes identified from Tibetan chickens under selection were also found to be overrepresented in the calcium signalling pathway^2^. Related significant pathways, such as hypertrophic cardiomyopathy and cardiac muscle contraction, and biological processes of development and morphogenesis in multiple systems, support widespread adaptive changes in physical characteristics over the long term. Accordingly, delayed growth and higher prevalence of underweight and stunting have been reported in high-altitude populations^30,31^. Moreover, studies have shown that Tibetan males have a deeper chest, and Tibetan females have a wider chest than the Han population^32^, evidence of morphological adaptation to obtain better oxygen delivery.

Moreover, the contribution of the immune system to LTA cannot be overlooked, as many infectious and autoimmune diseases were identified in the enrichment analyses. Given that infected and inflamed tissues often experience hypoxia, the cellular adaptive response to inflammation and immunity might be shared with pathways related to high-altitude adaptation, such as the HIF pathways^33-35^. The literature has also reviewed the complex immune response to hypoxia or high altitude from in vitro cell models to human and animal studies^36,37^. A consensus is that high-altitude exposure can suppress immune function and increase the risk of infectious^36-39^. A higher prevalence of rheumatoid arthritis among Tibetan highlanders has been reported^40^.

We noticed many enriched pathways implicating central nervous system, such as neuroactive ligand-receptor interaction, circadian entrainment, and morphine addiction. The brain, as one of the most metabolically active organs, is extremely sensitive to hypoxia^41^. The literature has shown that different types of neurotransmitter interactions can be affected by acute and/or chronic hypoxia, such as dopamine^42-44^, glutamate^45-47^, gamma-aminobutyric acid (GABA)^48^, and adenosine^49^. Our KEGG enrichment analysis for LTA DMSs showed that pathways of cholinergic synapse (p=0.08), glutamatergic synapse (p=0.07), and dopaminergic synapse (p=0.07) were highlighted. Cholinergic synapses are essential for transmitting signals from motor neurons to skeletal muscle fibres, leading to muscle contraction. They can regulate autonomic nervous system, modulate other neurotransmitter systems, and implicate cognitive and behavioural processes. Glutamate is the most abundant excitatory neurotransmitter, facilitating communication within neural circuits, enabling complex functions such as movement, sensation, and cognition. Dopaminergic synapses are involved in higher cognitive functions, such as attention, working memory, and decision-making. In addition, reward and motivation, and motor control are also the primary functions of dopaminergic synapses. Altogether, long-term exposure to high altitude has a substantial and profound influence on physical development, neuronal activity, and immune function.

### 5.3 The dissection of enrichment analyses for hypermethylated and hypomethylated DMSs

In the MWAS analyses, we observed apparent asymmetric patterns for both STA and LTA. We hypothesized that the hypomethylated probes and the hypermethylated probes might participate in different biological processes. Thus, we performed GSEA for the hypermethylated probes and hypermethylated probes, respectively.

For STA, the hypermethylated DMSs were marginally enriched in cancer-related pathways, such as breast cancer, microRNAs in cancer, and gastric cancer, while the hypomethylated DMSs were enriched in pathways of steroid biosynthesis, protein processing in endoplasmic reticulum, and cell cycle (Supplementary Tables S17-24). For LTA, the hypermethylated DMSs were enriched in the calcium signalling pathway and neuroactive ligand‒receptor interaction, and the hypomethylated DMSs were enriched in immune-related diseases and haematopoietic cell lineage. Consistently, the hypermethylated probes were enriched in GO biological processes of ion transport, morphogenesis, and development, while the hypomethylated signals were enriched in MHC protein complex assembly, and antigen processing and presentation (Supplementary Tables S9-16).

Considering that DNAm often supresses the expression of the target gene^50,51^, we posit hypotheses about the corresponding biological mechanisms. For STA, we hypothesize that cancer-related pathways would be suppressed, while stress reaction and cell cycle processes, such as the proliferation of vascular endothelial cells, would be activated. For LTA, we hypothesize that calcium signalling pathways and neuroactive ligand-receptor interaction would be suppressed, while immune-related pathways would play an active role.

## **Section 6:** Additional characteristics of the THCH meQTLs

The associations of 445,001 methylation probes with *cis*- and *trans*-genetic variants were tested within each group of the THCH cohort. We detected 36,582 *cis*-meQTL probes in ANs, 54,651 in NLs, and 61,326 in NHs at a stringent $P$ value threshold of $1\times{10}^{-8}$ (Supplementary Table S25). The correlation coefficients of the effect sizes of the top *cis*-meQTLs between pairwise groups, estimated by the $r_{b}$ method^52^, were all greater than 0.97 (Fig. 4b). This suggests high consistency of proximal genetic regulation of DNAm among groups in the THCH cohort.

To further increase the detection power, we merged the normalised phenotypes and performed *cis*-meQTL analysis for the full cohort. The full cohort yielded 88,780 meQTL probes at a $P$ value threshold of $1\times{10}^{-8}$, a 2.6-fold increase compared with the previous EAS cohort^53^, accounting for 20.0% of all tested DNAm probes. The phenotypic variance explained by the top variant ($R^{2}$) ranged from 0.022 to 0.809, and 4.9% of meQTL probes (4,392/88,780) had an $R^{2}$ larger than 0.5. The $R^{2}$ of top *cis*-meQTLs was inversely proportional to the distance between the variant and the probe (Supplementary Fig. S9). In addition, 15.7% of top meQTLs affected more than one probes (pleiotropy), and 26.5% of DNAm probes were affected by more than one causal variant identified by fine-mapping analysis (Supplementary Fig. S10). Regarding *trans*-meQTL, there were 2,394 significant probes being detected in the full cohort, containing 330,624 *trans*-meQTLs at $P$ value < $1\times{10}^{-14}$ (ref^54^; Supplementary Table S25). Of these, 37.6% of probes were also regulated by *cis*-variants simultaneously. For example, probe cg13328496 was associated with both local variants (rs1149764) in chromosome 10 and distal variants (rs10953800, rs12945137) in chromosomes 7 and 17 (Supplementary Fig. S11). The distribution of the top *trans*-meQTL was not uniform; they were prone to the telomere/sub-telomere region and chromosome 19, in concordance with the European discovery^55^ (Fig. 4a).

To valid the EAS meQTL results, we calculated the replication rate in the EUR cohort, which was 0.87 (50,620/57,965) for the *cis*-meQTLs and 0.82 (1,549/1,879) for the *trans*-meQTLs at a Bonferroni $P$value threshold. The meQTL effect size correlation between EAS and EUR, estimated by the $r_{b}$ method^52^, was 0.915 (SE 0.001) for the top *cis*-meQTLs and 0.914 (SE 0.006) for top *trans*-meQTLs (Fig. 4b; Supplementary Fig. S12). Despite the high consistency in the genetic regulation of DMSs between EAS and EUR, 23.6% EAS *cis*-meQTLs showed population-specific effect sizes through the heterogeneity test. We provided two typical examples of population-specific signals: a likely EAS-specific signal for probe cg20132862 on chromosome 11 and a likely EUR-specific signal for probe cg04954056 on chromosome 7 (Supplementary Fig. S14).

We then performed functional enrichment analyses for the identified meQTLs in the THCH cohort. Considering the distance between meQTLs and DNAm probes, we used a permutation-based method to compare the top *cis*-meQTLs with the top *cis*-variants of overall DNAm probes^56^. We found that *cis*-meQTLs were significantly enriched in active enhancer (OR=1.20, 95% CI: 1.18-1.22), DNase (OR=1.19, 95% CI: 1.10-1.30), heterochromatin (OR=1.18, 95% CI: 1.15-1.21), and polycomb repressed (OR=1.15, 95% CI: 1.14-1.17) regions, and depleted in the transcription (OR=0.78, 95% CI: 0.77-0.79), flanking promoter (OR=0.76, 95% CI: 0.75-0.77), and transcription start site (TSS; OR=0.73, 95% CI: 0.71-0.76) regions. In contrast, the top *trans*-meQTLs, which were compared with the top variants of randomly selected probes, were enriched in the TSS (OR=10.81, 95% CI: 2.87-91.30), zinc finger gene states (OR=7.16, 95% CI: 3.19-19.95), and flanking promoter (OR=4.81, 95% CI: 2.35-11.45) regions, whereas they were depleted in enhancer (OR=0.78, 95% CI: 0.63-0.98), acetylation (OR=0.55, 95% CI: 0.42-0.72) and quiescent (OR=0.42, 95% CI: 0.35-0.49) regions (Fig. 4c-d). The mentioned characteristics of the THCH meQTLs was in concordance with those observed in previous meQTL studies in samples of predominantly European ancestry, validating the quality of our data.

## **Section 7:** The effectiveness of utilizing the THCH meQTLs to identify functional elements associated with complex traits in samples of EAS ancestry

We showcased the performance of leveraging the meQTLs to identify functional elements underlying genome-wide association study (GWAS) signals for complex traits and diseases in samples of EAS ancestry. We first used SMR, a method that can detect association between a molecular phenotype, such as transcriptional abundance or DNAm, and a complex trait by integrative analysis of GWAS and xQTL (molecular quantitative trait locus) summary data, to compare the number of prioritised probes using meQTL datasets from the THCH cohort from the present study (*N*=1,448, EAS ancestry), the LBC+BSGS cohort^55^ (*N*=1,980, EUR ancestry), and GoDMC^54^ (*N*=27,750, EUR ancestry). Using the in-sample LD reference (genotypes from the THCH genotypes), there were ~32% SNPs with allele frequency differences > 0.2 when using EUR meQTL datasets. The largest meQTL dataset to date, GoDMC, prioritized approximately twice as many DNAm probes compared to the other two cohorts. The THCH cohort nominated slightly more DNAm probes than when using meQTL summary statistics from the LBC+BSGS cohort. Therefore, sample size is a determinant for the detection power of SMR analysis. However, the importance of matched ancestry was demonstrated through the heterogeneity in dependent instruments (HEIDI) test. Filtering out the probes that do not pass the HEIDI test at the threshold of 0.01, the prioritized signals decreased by 72.4% for the LBC+BSGS cohort, while the number was 61.5% for the THCH cohort (Supplementary Fig. S13).

We then conducted colocalization analyses to compare the number of meQTL signals colocalized with GWAS signals. The method features the flexibility of assumptions and is free from using an LD reference. As the meQTL summary data from GoDMC had only *P* < 1$\times{10}^{-5}$ results available, we excluded the GoDMC data from this the comparison. On average, using the meQTL dataset from the THCH identified twice the number of colocalized probes as the LBC+BSGS cohort (an increase of 66.9% to 181.5% across 32 traits) with PP4 statistics > 0.8 (Fig. 4e).

The improvement of using matched ancestry for GWAS and meQTLs is partially owing to the consistency in MAF and LD. One example is cg19379178 for the trait of HDL cholesterol, which was only prioritized using EAS-meQTL but was omitted using EUR datasets in the SMR and HEIDI test, although the visualisation of the overall signal patterns is similar for both datasets (LBC+BSGS cohort: *P*_SMR_ = $4.6\times{10}^{-4}$, *P*_HEIDI_ =$4.3\times{10}^{-5}$; THCH cohort: *P*_SMR_ = $3.3\times{10}^{-11}$, *P*_HEIDI_ =0.21; Supplementary Fig. S15a-b). Colocalization analysis showed consistent results with a COLOC PP4 statistic of 0.62 using the EUR dataset and 0.96 using the EAS dataset. Moreover, some detected DNAm signals appeared to be due to EAS-specific meQTLs. Using cg10893667 as an example for the trait of red blood cell count, the COLOC PP4 statistic was $1.0\times{10}^{-4}$ using the EUR dataset but 0.77 using the EAS dataset, indicating EAS-specific colocalization. SMR analysis also supported the EAS-specific signal (LBC+BSGS cohort: *P*_SMR_ = $6.1\times{10}^{-4}$, *P*_HEIDI_ =0.11; THCH cohort: *P*_SMR_ = $2.4\times{10}^{-8}$, *P*_HEIDI_ =0.09; Supplementary Fig. S15c-d). The above analysis emphasizes the effectiveness of utilising the THCH meQTLs to identify functional elements for EAS ancestry.

## **Section 8:** Sensitivity analyses for the AAR difference test

To control for potential confounding effect of gender, we tested the AAR differences among the three groups within the female samples (sample sizes: N=544 in NHs; N=287 in ANs; N=246 in NLs). There was a significant difference in AAR among the three groups ($P$value = $4.8\times{10}^{-12}$). In pairwise comparisons, ANs showed a significantly larger AAR compared to NHs and NLs ($P$value = $1.8\times{10}^{-9}$ and $1.4\times{10}^{-11}$, respectively). No significant difference in AAR was observed between the two adaptive groups, NLs and NHs ($P$value= 0.19).

To control for potential batch effects, we tested the AAR differences among the three groups within experimental batches. In batch 1, ANs and NHs showed significant AAR differences ($P$value = $1.8\times{10}^{-9}$). In batch 2, ANs had a significantly larger mean AAR compared to NHs and NLs ($P$value = $0.05$ and $0.03$, respectively), but no significant difference was observed between NLs and NHs ($P$value= 0.04). In batch 3, ANs showed a significantly larger mean of AAR compared to NHs and NLs ($4.2\times{10}^{-4}$ and $4.8\times{10}^{-3}$, respectively).

We further pre-adjusted AAR for gender, batch, and chronological age simultaneously and then tested mean differences in the residuals among the three groups, obtaining consistent results. In the pairwise comparisons, ANs had a significantly larger mean of AAR compared with NHs and NLs ($P$value = $2.6\times{10}^{-6}$ and $3.2\times{10}^{-7}$, respectively). No significant difference was found between the two adaptive groups, NLs and NHs ($P$value= 0.85). The above sensitivity analyses indicate that the observed accelerated ageing in the ANs was unlikely to be biased by factors such as gender, batch, and chronological age distribution.

## **Section 9:** Replication analyses using the commonly used epigenetic age predictors

Except for the epigenetic age predictor from the Zhang et al.’s BLUP model, we performed replication analyses using the Zhang et al.’s EN model, Horvath et al.’s multi-tissue-based model^57^, Hannum et al.’s single-tissue-based model^58^, and Levine et al.’s PhenoAge model^59^. In the application to the THCH cohort, we found that the prediction accuracy was highest for the BLUP model, followed by the EN model, single-tissue-based model, multi-tissue-based model, and PhenoAge model (Supplementary Fig. S16a).

In the statistical tests of AARs among the three groups, all models indicated that ANs had a larger mean of AAR than NLs (except for the multi-tissue-based model which did not yield a significant difference). Besides, all models showed that NHs had a significantly larger mean of AAR than NLs except for the BLUP model (Supplementary Fig. S16b). The discrepancy is largely due to the distinction of the epigenetic clock design. A review paper has systematically summarized the pros and cons of different epigenetic clocks^60^. For example, the multi-tissue-based model can predict age in multiple tissues but has a week association with age-associated disease conditions. In contrast, the PhenoAge model was developed to estimate biological age based on a combination of clinical biomarkers. Therefore, the results above, in general, consistently suggest that the acclimatised newcomers have experienced accelerated ageing. In addition, the PhenoAge model appeared to be a better predictor of mortality risk, indicating that native highlanders may have received adverse effects from lifelong high-altitude exposure.

## **Section 10:** Genome-wide association study for AAR

To investigate the genetic regulation of AAR, we performed a GWAS for the trait of normalized AAR, which is a scaled residual after regressing out gender, chronological age, group (NHs/ANs), and batch. Using GCTA-fastGWA^61^, we detected no SNP passing the conventional threshold of $5.0\times{10}^{-8}$, possibly due to the lack of statistical power to detect the polygenic effects for AAR (Supplementary Fig. S17). However, there were 10 independent loci being detect at the suggestive threshold of $5.0\times{10}^{-5}$. Among these, the nearest gene *TRIM71* of rs67034073 in chromosome 3 is involved in the G1-S phase transition of the cell cycle (*https://www.uniprot.org/uniprotkb/Q2Q1W2/entry*). The nearest gene *P4HA2* of rs537454255 in chromosome 5 is included in the GO annotation of oxidoreductase activity and electron transfer activity (*https://www.uniprot.org/uniprotkb/O15460/entry*). The nearest gene *FBXW11* of rs6890278 in chromosome 5 relates to oxidative stress-induced a ubiquitin-mediated decrease in *RCAN1 (https://www.uniprot.org/uniprotkb/Q9UKB1/entry)*. This analysis provides insights into the genetic basis of accelerating ageing, potentially relating to biological processes such as the cell cycle and oxidative stress.

## **Section 11:** EAS-meQTL results using different imputation reference panels

We compared *cis*-meQTL results using genotype imputed from two different reference panels, ChinaMAP (Build hg38) and 1000 Genome phase 3 (1000G, Build hg19). For the full cohort, 88,989 significant *cis*-meQTL probes were identified using 1000G, and 88,780 using ChinaMAP, with 84,300 overlapping probes between the two panels. The effect size correlation for the top *cis*-meQTL was 0.953 [95% CI: 0.952-0.954]. For those overlapping probes, the identified meQTLs were slightly closer to the tested probes and had a significant higher R^2^ for ChinaMAP. The median distance between the top meQTL and the tested probe was 5.0 Kb for ChinaMAP and 8.1 Kb for 1000G. The mean R^2^ was 0.146 for ChinaMAP and 0.141 for 1000G (paired t-test *P*-value < $2.2\times{10}^{-16}$), suggesting that the imputed genotypes from ChinaMAP might be slightly more accurate.

# **Supplementary Figures**

**Supplementary Figure S1:** The distribution of highland living years among acclimatized newcomers. The x-axis represents the duration of highland living in years, while the y-axis represents the frequency of occurrence. An enlarged histogram is displayed in the top right corner of the Figure, focusing specifically on individuals with less than 10 years of highland living experience.


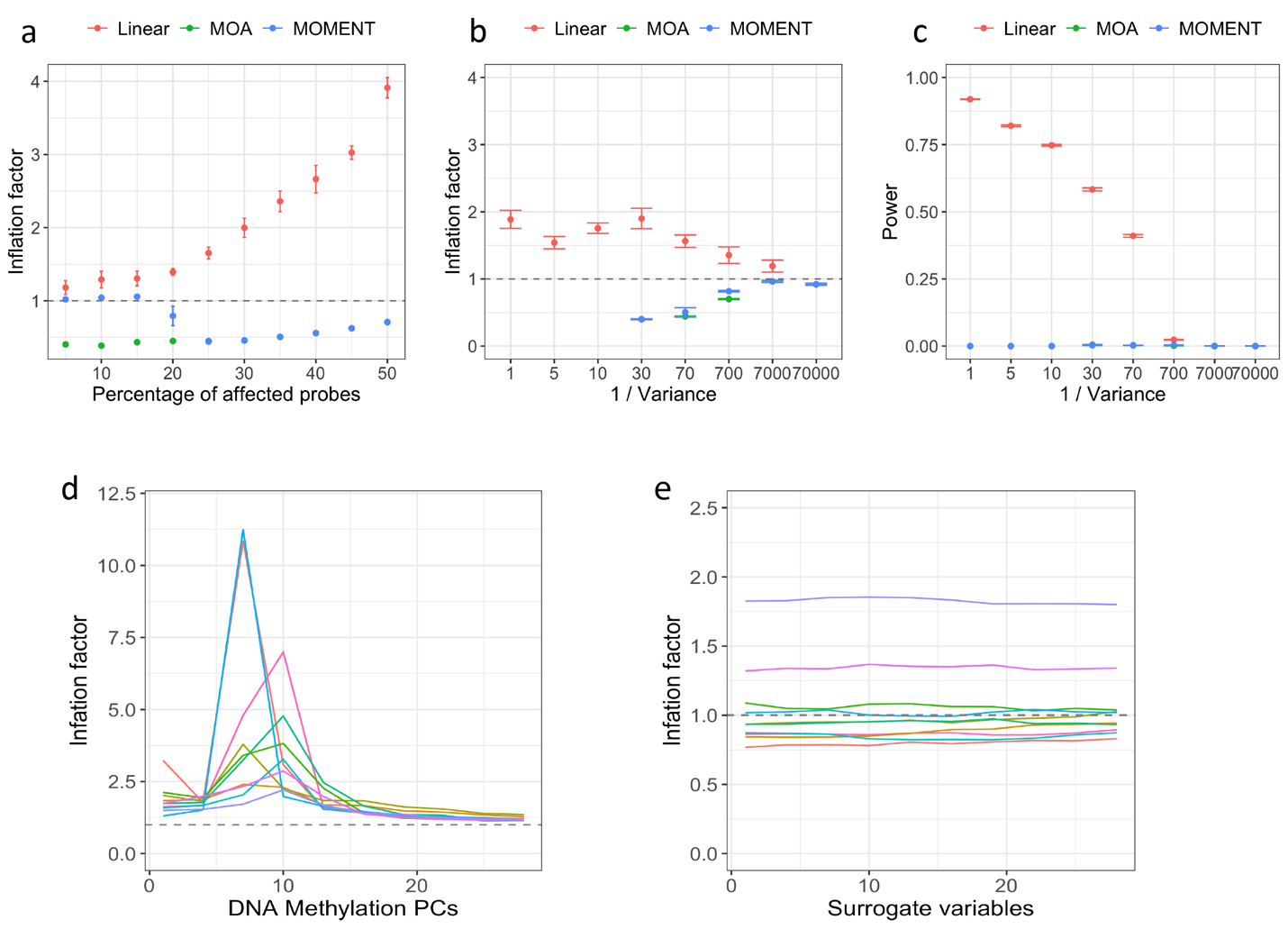


**Supplementary Figure S2:** Simulation results for MWAS method evaluation. **a** Inflation factors (y-axis) in the simulation scenarios with different proportions of affected DNAm probes (x-axis). The effect size distribution was a normal distribution with the mean of 0 and the variance of 1/70; **b** Inflation factors (y-axis) in the simulation scenarios with different effect size distributions (x-axis). The percentage of affected probes was fixed at 21%. **c** Statistical power (y-axis) in the simulation scenarios with different effect size distributions (x-axis). The linear regression model, mixed-linear models of mix-linear model (MLM)-based omic association (MOA) and multi-component MLM-based association excluding the target (MOMENT) are labelled with different colours. The performance is illustrated as the mean and error bar of the standard error (SE) of 10 simulation replicates. **d** Inflation factor (y-axis) of the linear regression model with different numbers of principal components (PCs) as covariates (x-axis). **e** Inflation factor (y-axis) of the linear regression model with different numbers of surrogate variables (SVs) as covariates (x-axis). Each colour represents one repeat in the scenario with 21% of affected probes and variance of 1/30 in the effect size distribution.

**Supplementary Figure S3:** Simulation results of the Bacon method. The x-axis represents the percentage of DNAm affected by the phenotype, the y-axis represents the strength of the confounder, and the z-axis and color represent the number of false positives after Bacon adjustment. Each panel represents the percentage of probes affected by the confounders.

**Supplementary Figure S4:** The simulation results of adding the confounder as a covariate. The x-axis represents the percentage of DNAm affected by the phenotype, the y-axis represents the strength of the confounder, and the z-axis and color represent the number of false positives before Bacon adjustment but adding the confounder as a covariate. Each panel represents the percentage of probes affected by the confounder. There is almost no false positive after adding the confounder as a covariate.


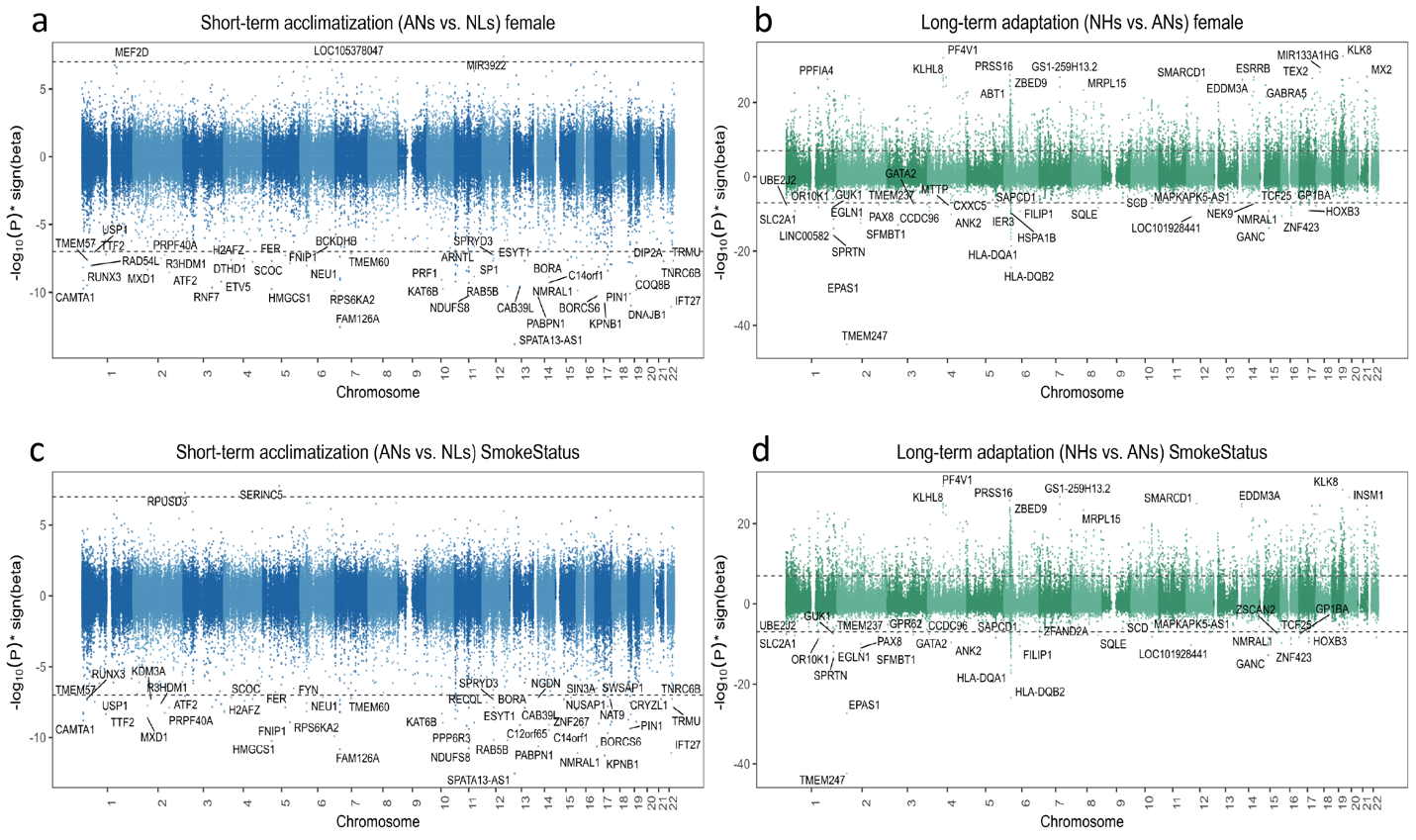


**Supplementary Figure S5:** Manhattan plots of the MWAS sensitivity analyses. The x-axis denotes the genomic position, and the y-axis denotes the MWAS association $-{log}_{10}(bacon \mathrm{adjusted} P \mathrm{value})$ times the sign of the effect size. The dashed lines indicate the methylome-wide significance thresholds ($bacon-\mathrm{adjusted}P$value = $1.12\times{10}^{-7}$). DMSs were annotated to the nearest genes. **a** MWAS results of STA for the females only; **b** MWAS results of LTA for the females only; **c** MWAS results of STA with predicted smoking status added as a covariate; **d** MWAS results of LTA with predicted smoking status added as a covariate.


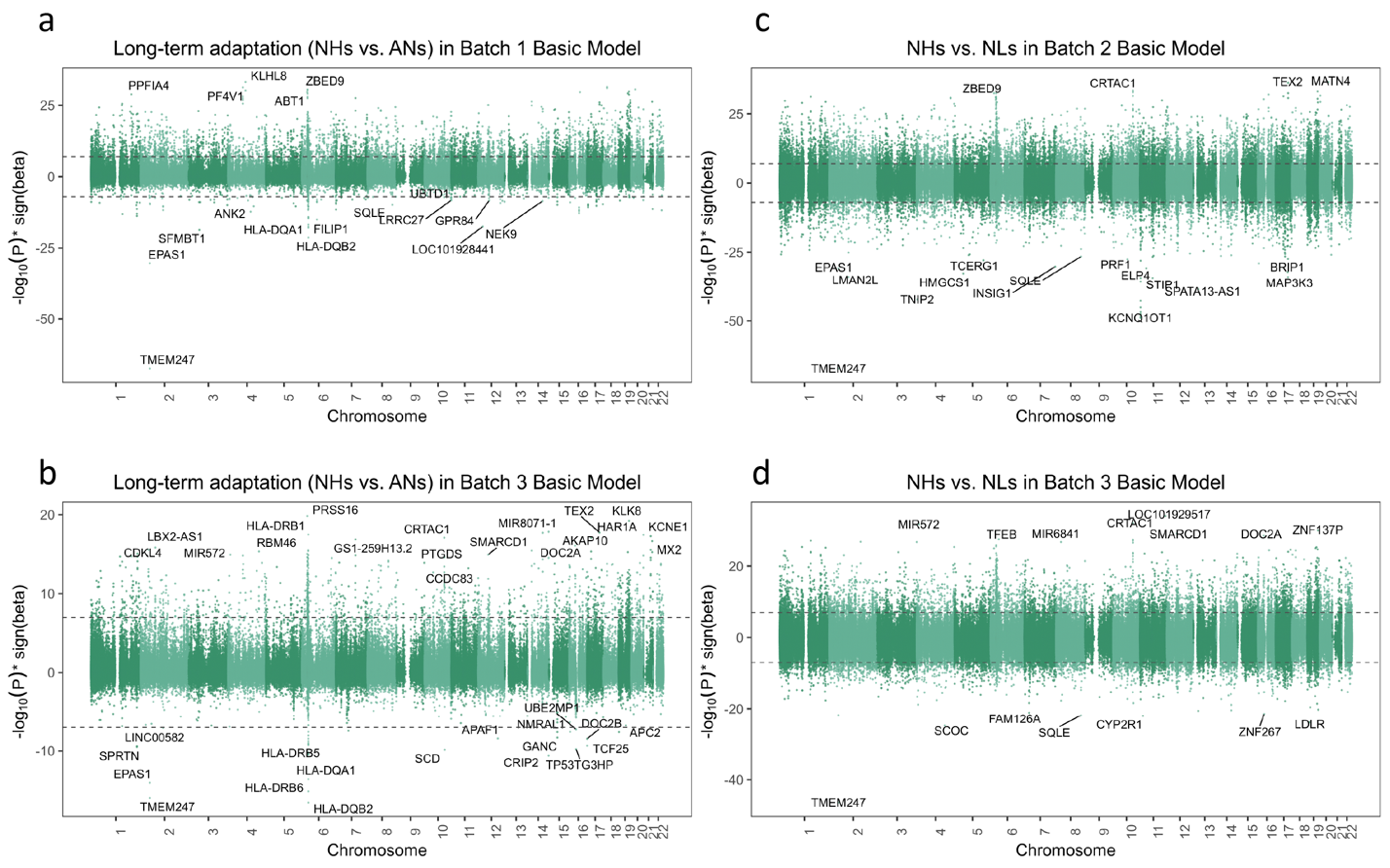


**Supplementary Figure S6:** Manhattan plots of MWAS results within experimental batches. The x-axis denotes the genomic position, and the y-axis denotes the MWAS association $-{log}_{10}(P \mathrm{value})$ times the sign of the effect size. The dashed lines indicate the methylome-wide significance thresholds ($P$value = $1\times{10}^{-7}$). DMSs were annotated to the nearest genes. Note that the $P$values in the Figures were not adjusted by the *bacon* method. **a** MWAS results of LTA using samples in Batch 1; **b** MWAS results of LTA using samples in Batch 3; **c** MWAS results comparing NHs and NLs using samples in Batch 2; **d** MWAS results comparing NHs and NLs using samples in Batch 3.

**Supplementary Figure S7:** Boxplots of cell type proportion estimated from DNAm for the Tibetan and Han Chinese. The y-axis represents the estimated cell type proportion, while the x-axis shows the three groups. The panels in the Figure correspond to different cell type categories, namely, B cells, CD4^+^ T cell, CD8^+^ T cell, Eosinophils, Monocytes, Neutrophils, and Natural Killer cells.


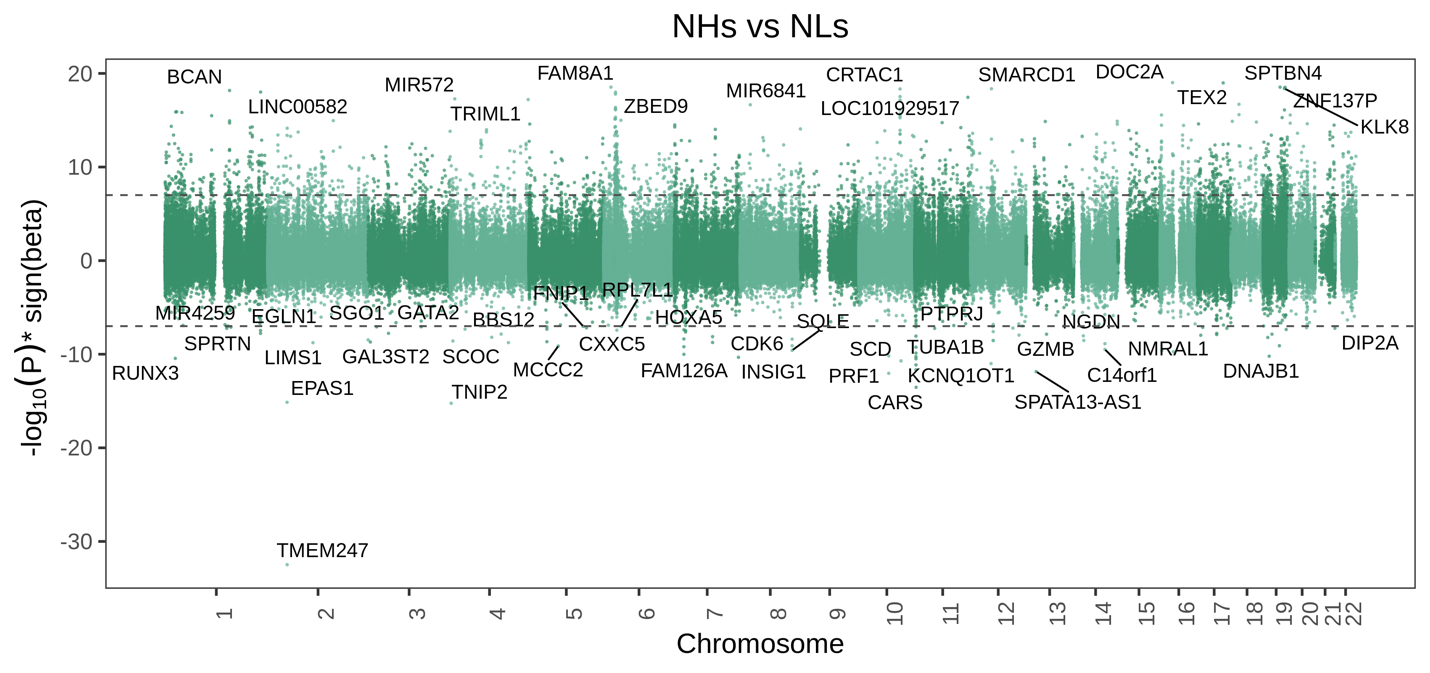


**Supplementary Figure S8:** Manhattan plot of MWAS results comparing NHs and NLs. The x-axis denotes the genomic position, and the y-axis denotes the MWAS association $-{log}_{10}(bacon-adjusted P \mathrm{value})$ times the sign of the effect size. The dashed lines indicate the methylome-wide significance thresholds (*bacon*-adjusted $P$value = $1\times{10}^{-7}$). DMSs were annotated to the nearest genes.


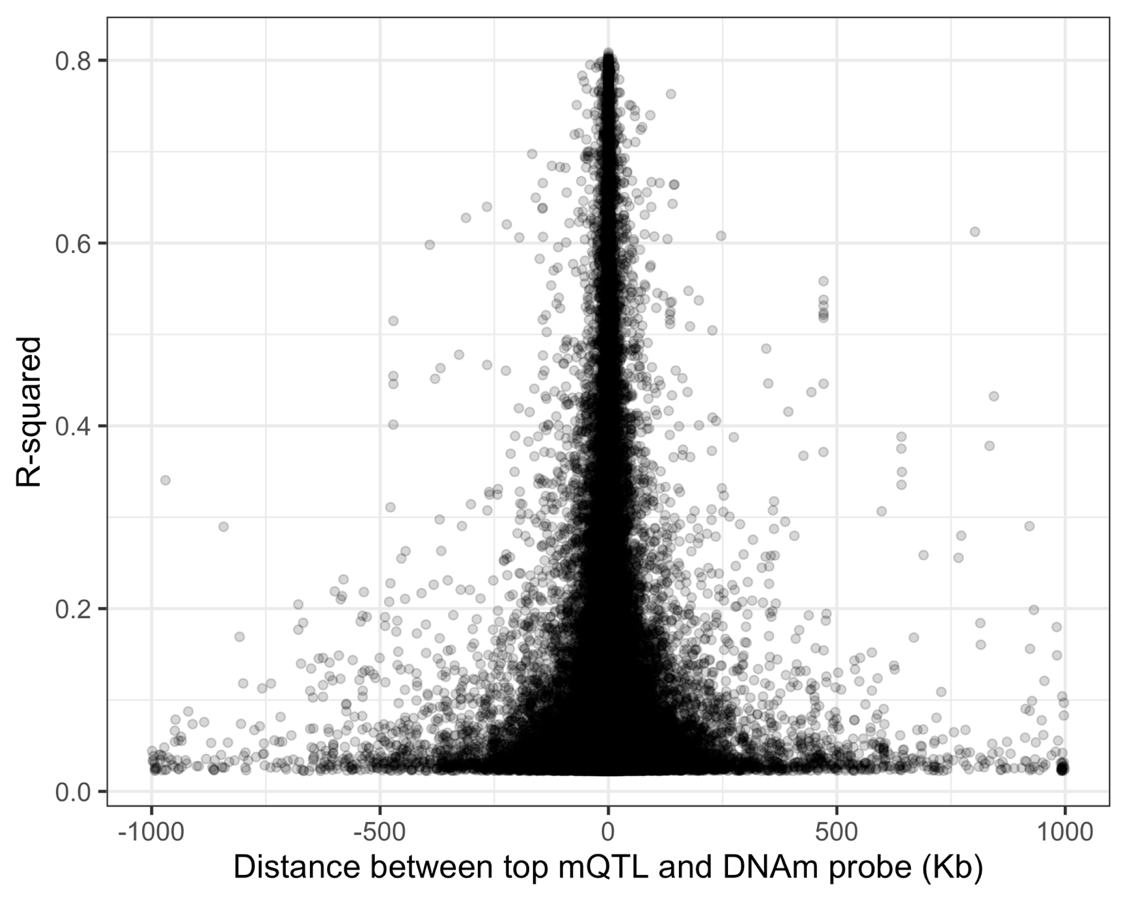


**Supplementary Figure S9:** Scatter plot of the coefficient of determination against the distance between DNAm probes and the corresponding top *cis*-meQTLs. The y-axis denotes the coefficient of determination (R-squared), which is the proportion of variance in DNAm explained by the corresponding top *cis*-meQTLs. The x-axis shows the distance in kilobases (Kb) between DNAm probes and the corresponding top *cis*-meQTLs. The R-squared were inversely proportional to the distances between the DNAm probes and the corresponding top *cis*-meQTLs.


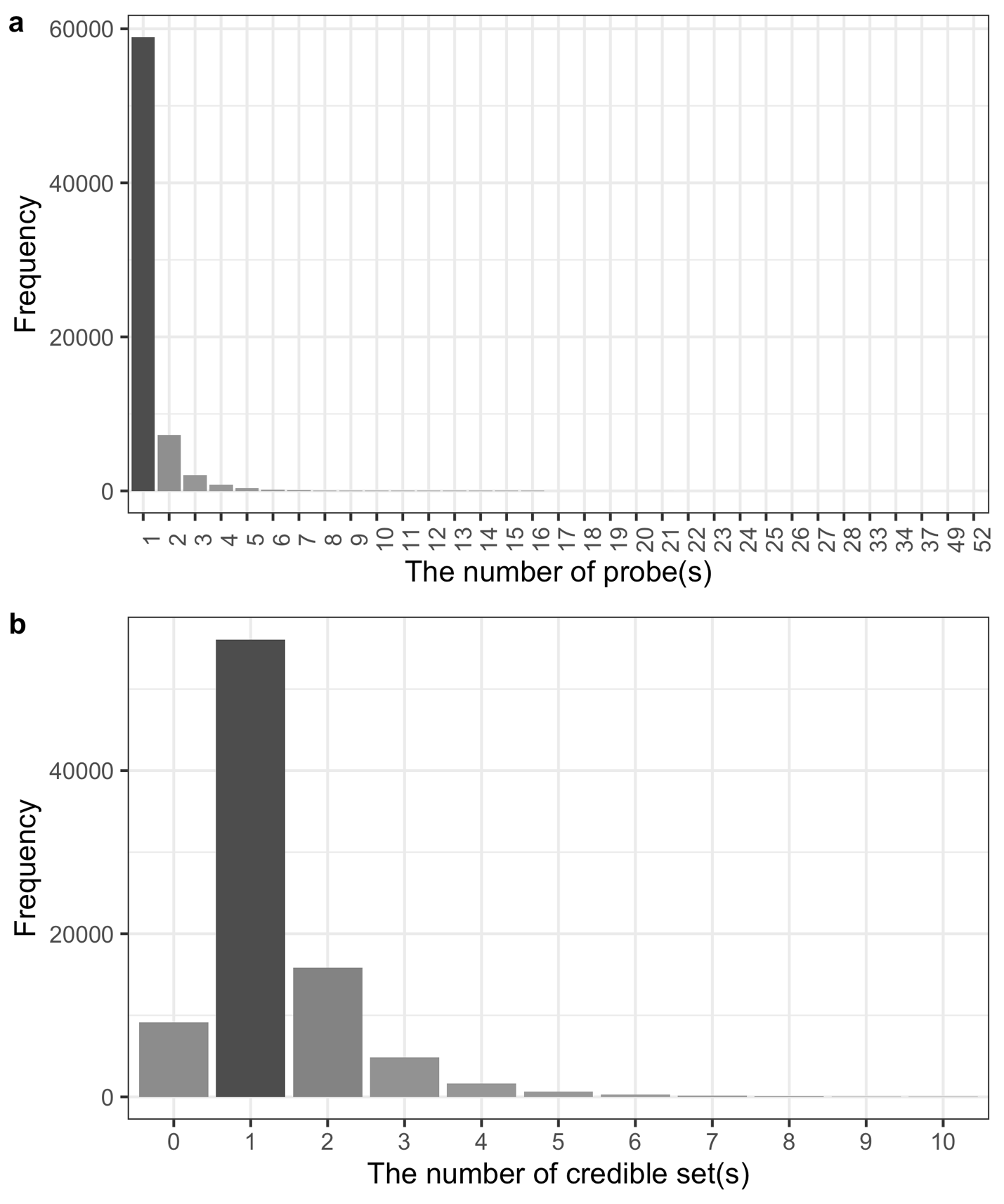


**Supplementary Figure S10:** Distribution of pleiotropic variants and credible set(s) of *cis*-meQTLs. **a** Bar plot showing the number of DNAm probe(s) regulated by each top *cis*-meQTL. 15.7% of the top meQTLs affected more than one probe (i.e., exhibited pleiotropy). **b** Bar plot showing the number of credible set(s) for all identified *cis*-meQTL regions. 26.5% of DNAm probes were affected by more than one causal variant, as determined by fine-mapping analysis.


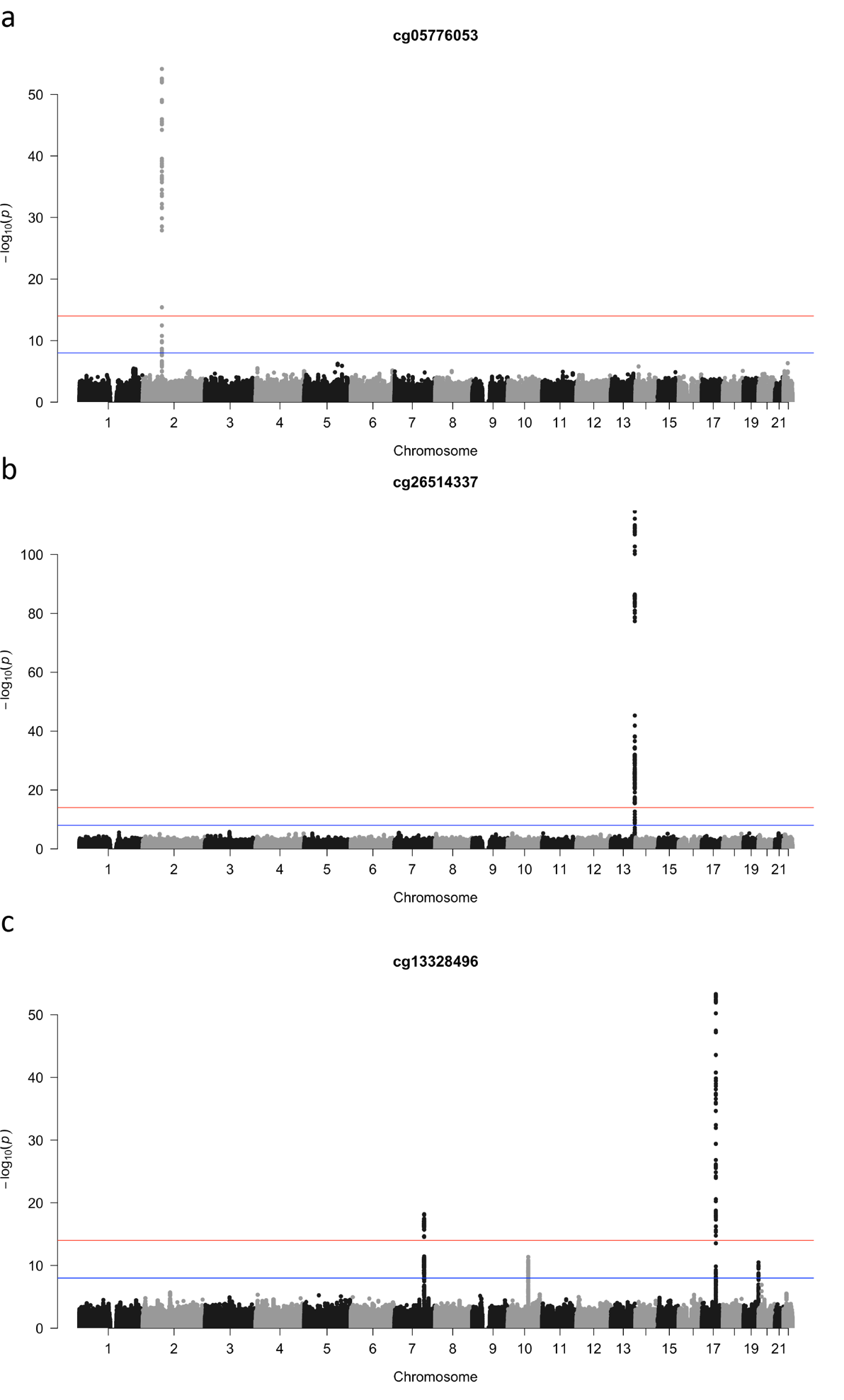


**Supplementary Figure S11:** Genome-wide scan of three typical meQTL examples. **a** Manhattan plot of the genetic regulation of probe cg05776053 located on chromosome 2, where only *cis*-genetic loci were identified. **b** Manhattan plot of the genetic regulation of probe cg26514337 located on chromosome 18, where only *trans*-genetic loci were identified. **c** Manhattan plot of the genetic regulation of probe cg13328496 located on chromosome 10, which was simultaneously regulated by *cis*- and *trans*-meQTLs. The blue line indicates *P* = $1\times{10}^{-8}$, and the red line indicates *P* =$1\times{10}^{-14}.$


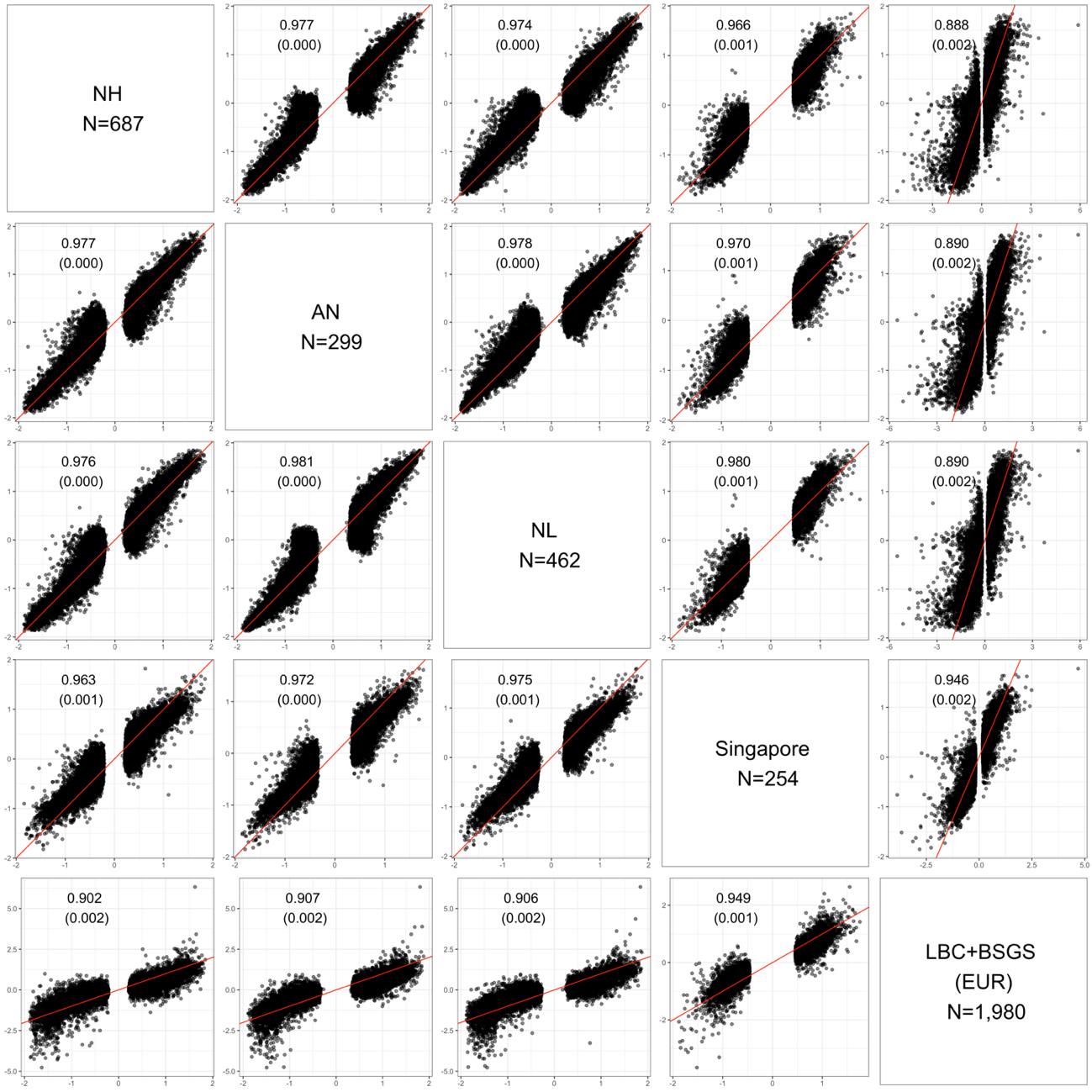


**Supplementary Figure S12:** Scatter plots of pairwise effect sizes of meQTLs from different groups or cohorts. The x-axis shows the SNP effect sizes of the significant top *cis*-meQTLs in the discovery cohort, while the y-axis shows the corresponding SNP effect sizes in the replication cohort. The numbers displayed at the top left are the genetic correlations estimated by the *r_b_* method, with the standard error shown in parentheses. This analysis included three datasets/populations: the THCH data from the present study (Chinese, EAS, including the groups of NHs, ANs, and NLs), the Singapore iOmics data (Southeast Asians), and a meta-analysis of the Lothian Birth Cohort (LBC) and the Brisbane Systems Genetics Study (BSGS) data (EUR ancestry).


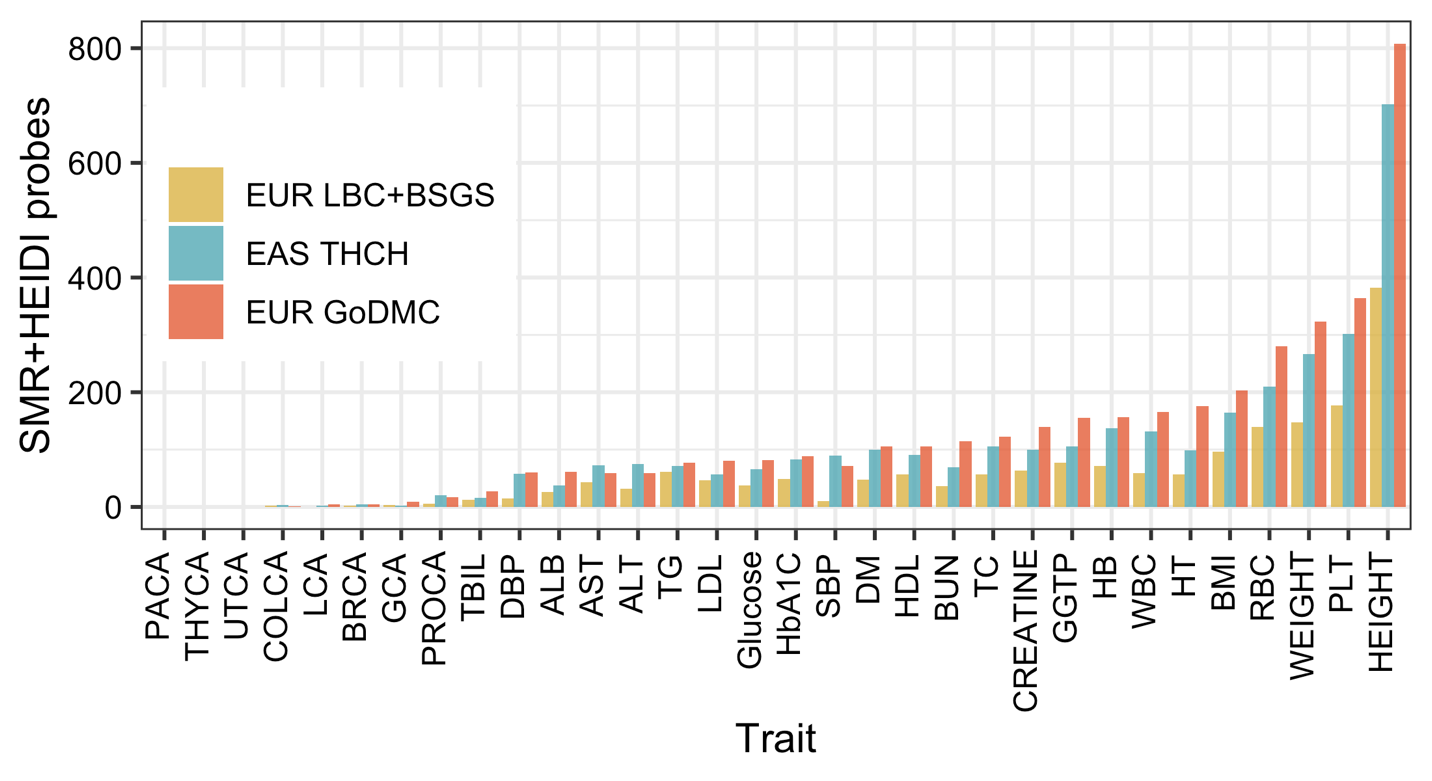


**Supplementary Figure S13:** Bar plot showing the number of prioritised DNAm probes associated with 32 common traits using SMR and HEIDI. The x-axis shows the acronyms for the 32 common traits from the meta-analysis of Biobank Japan and Korean Biobank. The y-axis shows the number of prioritised DNAm probes at the Bonferroni threshold of *P*_SMR_ < (0.05/the number of tested probes) and *P*_HEIDI_ > 0.01. The colours indicate different meQTL datasets used in the analysis.


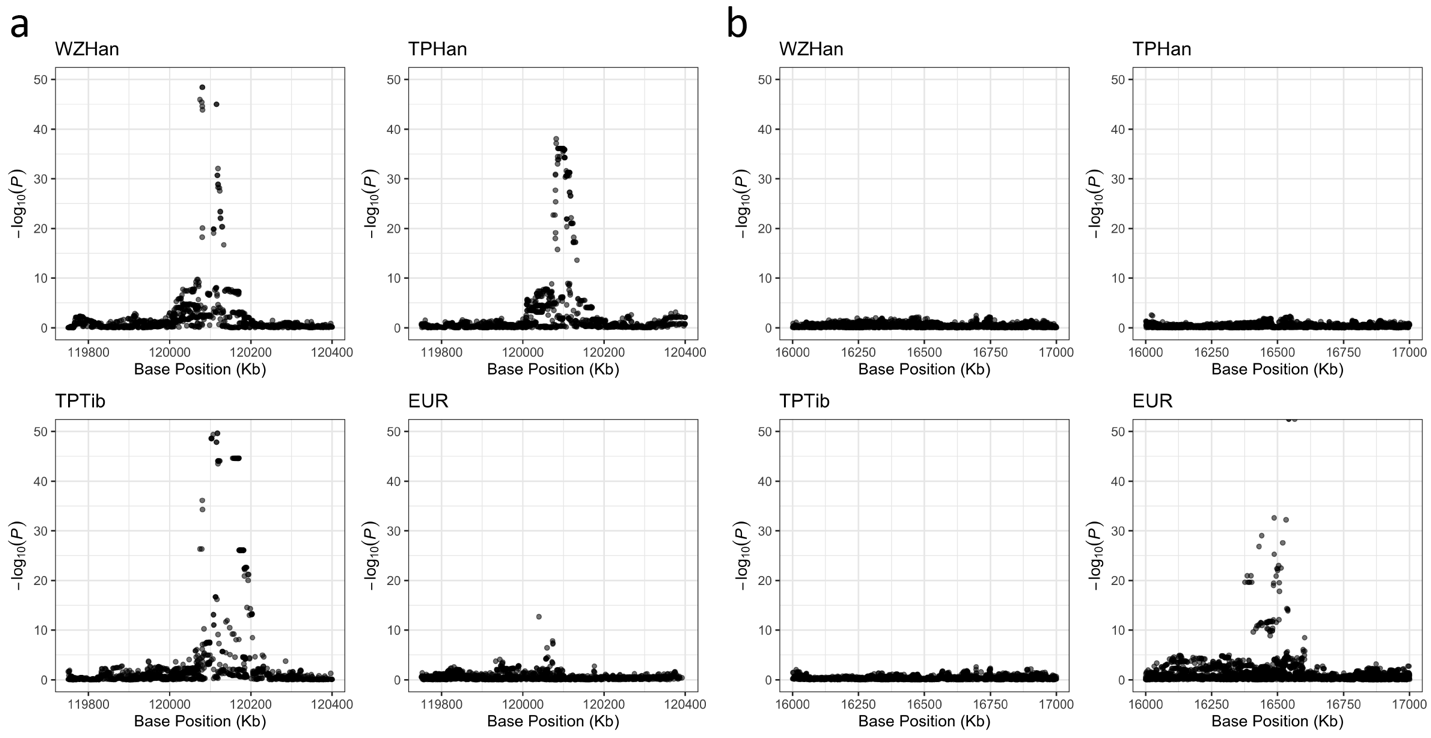


**Supplementary Figure S14:** Examples of population-specific meQTLs. The x-axis represents the base position in kilobases (Kb), and the y-axis denotes the -log_10_(*P* value) of the meQTL results. **a** LocusZoom plots show the EAS-specific meQTL cg20132862. In EAS, the top variant, rs12419706, had a $P$-value of 3.$1\times{10}^{-169}$ compared to rs12421218 in EUR, which had a $P$-value of $2.0\times{10}^{-13}$; **b** LocusZoom plots show the EUR-specific meQTL cg04954056. In EUR, the top variant, rs7796646, had a $P$-value of was very close to 0 (Z score = -40.59) compared to rs7798302 in EAS, which had a $P$-value of $5.1\times{10}^{-3}$. The cohorts shown in the Figures include the THCH cohort (Chinese, EAS, including the groups of NHs, ANs, and NLs), and the LBC + BSGS cohort (EUR ancestry).


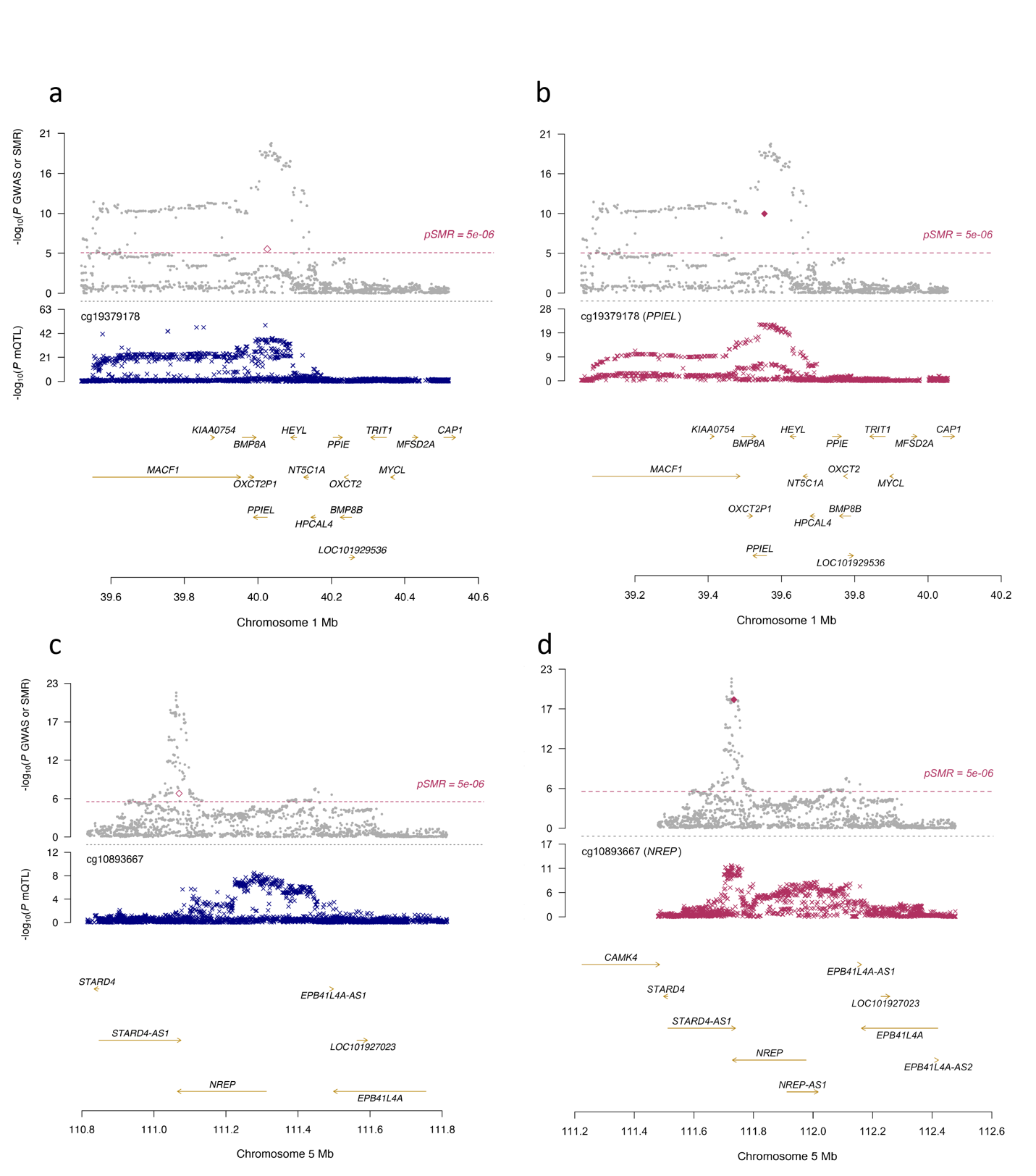


**Supplementary Figure S15:** Examples of EAS-specific DNAm probes associated with traits prioritized by SMR analysis. **a**-**b** demonstrate an EAS-specific signal due to the LD consistency. **c**-**d** demonstrate an EAS-specific signal due to EAS-specific meQTLs. **a** Probe cg19379178 for HDL cholesterol using the EUR LBC+BSGS meQTL dataset. **b** Probe cg19379178 for HDL cholesterol using the EAS THCH meQTL dataset. **c** Probe cg10893667 for red blood cell count using the EUR LBC+BSGS meQTL dataset. **d** Probe cg10893667 for red blood cell count using the EAS THCH meQTL dataset. The top track shows −log_10_(*P* values) of SNPs from the GWAS, and the second track shows −log_10_(*P* values) of SNPs from the meQTLs. The LD reference and GWAS results were from the EAS cohort. Note that, except from the difference in meQTL datasets, EAS used dataset edition (Build hg38) and EUR used dataset edition (Build hg19) for computational convenience.


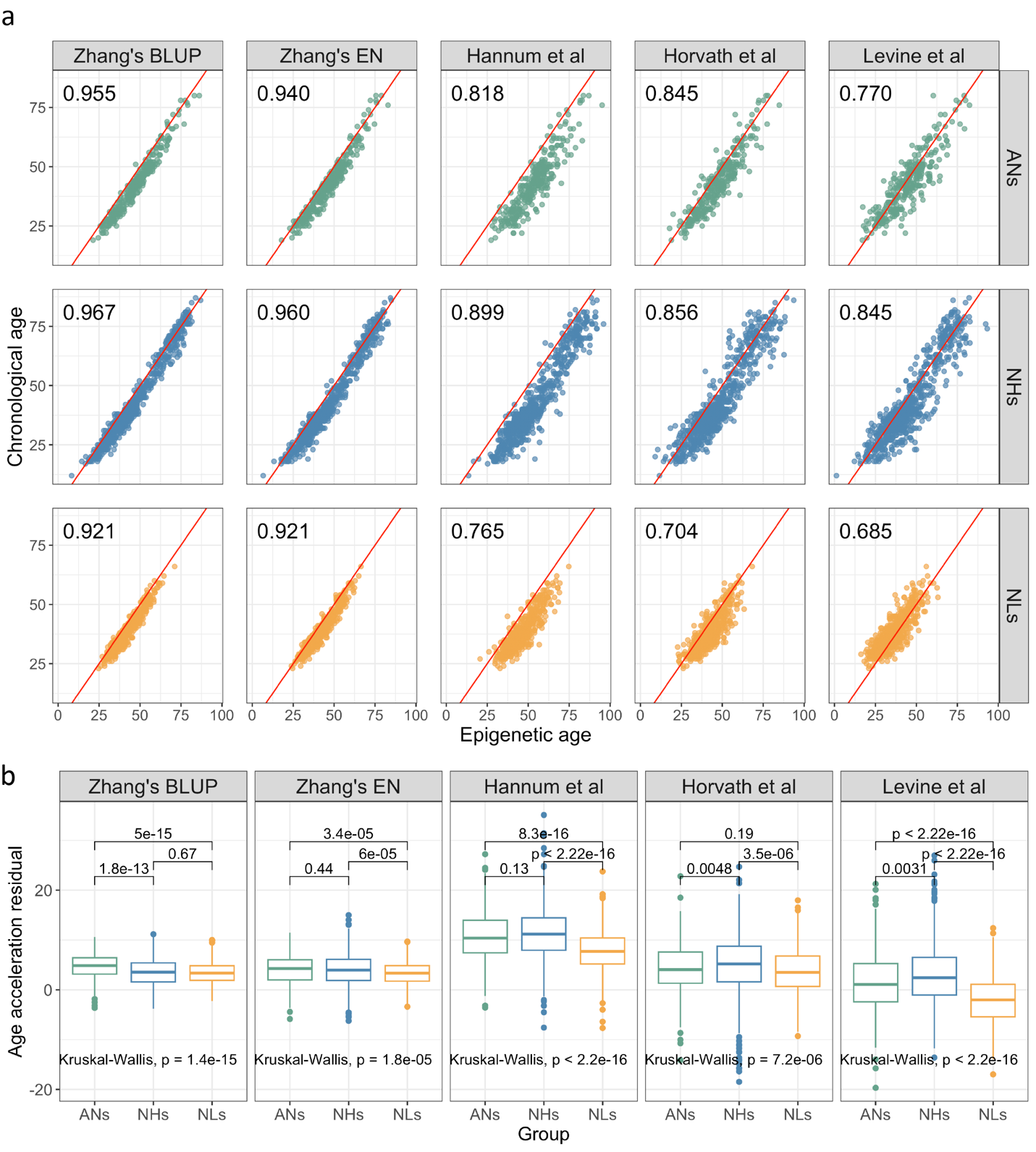


**Supplementary Figure S16:** Predictive performance of epigenetic age predictors and age acceleration residual (AAR) tests. **a** Scatter plots of chronological age (y-axis) against epigenetic age (x-axis) generated from the five predictors (in the column panel) for the three groups (in the row panel). Each dot represents an individual. The red line represents a slope of 1 with an intercept of 0. The number at the top-left of the panel is the coefficient of determination *R*^2^. **b** Box plot of AAR of the three groups from the five predictors. The $P$-values above the box represent the statistical significance of the pairwise differences in mean AAR between the two groups indicated. The $P$-value below the box represents the significance of the difference in mean AAR among all the three groups. Note that outliers exceeding 1.5 times the interquartile range (IQR) were removed.


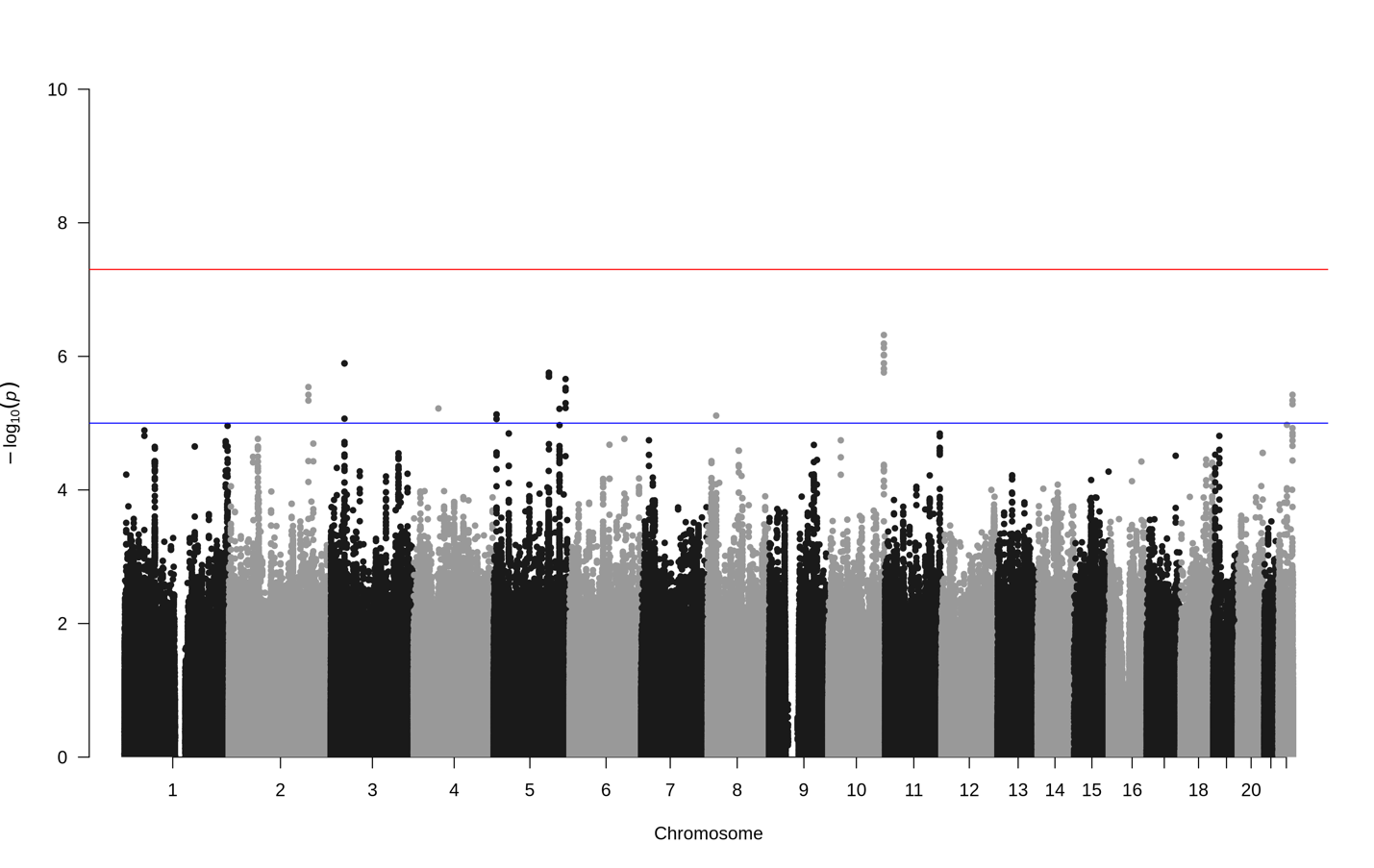


**Supplementary Figure S17:** Manhattan plot of GWAS results for age acceleration residual (AAR) in the THCH cohort. The x-axis denotes the genomic position, and the y-axis denotes the GWAS association $-{log}_{10}(P \mathrm{value})$. The red line indicates the genome-wide significant threshold ($P$value = $5\times{10}^{-8}$). The blue line indicates the suggestive threshold ($P$value = $1\times{10}^{-5}$).


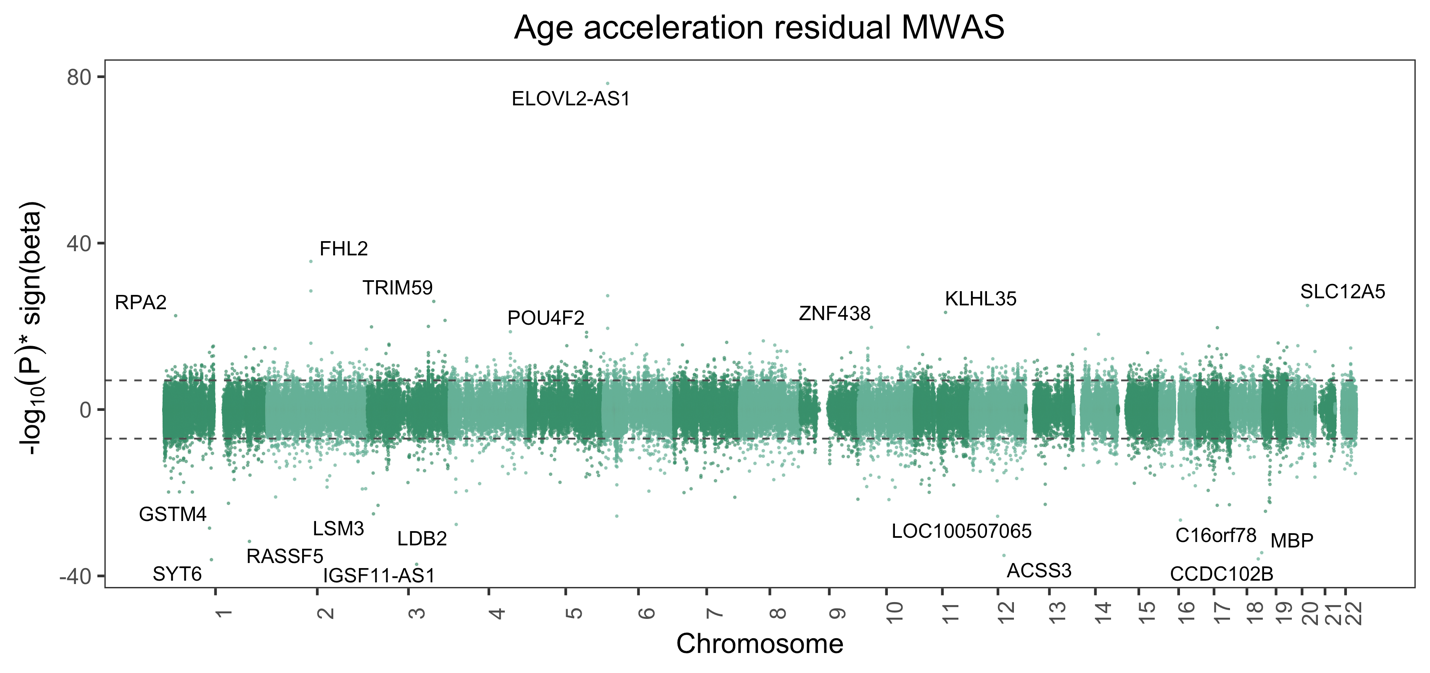


**Supplementary Figure S18:** MWAS analyses for age acceleration residual. The x-axis denotes the genomic position, and the y-axis denotes the MWAS association $-{log}_{10}$($P$value) times the sign of the effect size. The dashed lines indicate the methylome-wide significance thresholds (bacon-adjusted $P$value = $1.12\times{10}^{-7}$). DMSs were annotated to the nearest genes.


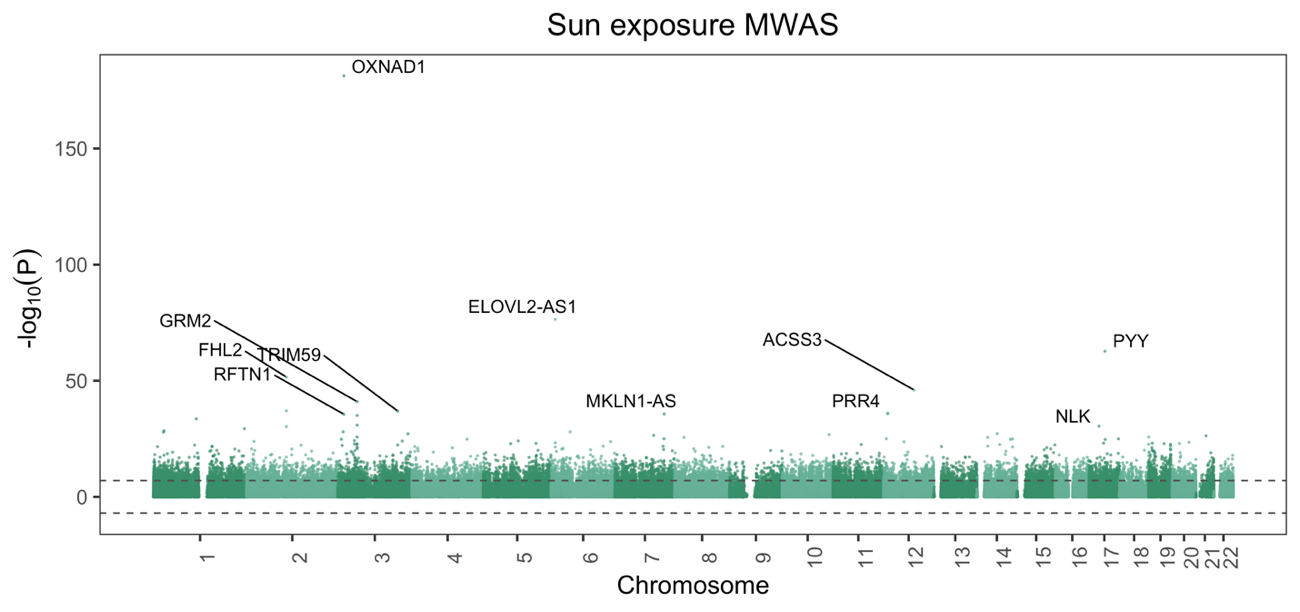


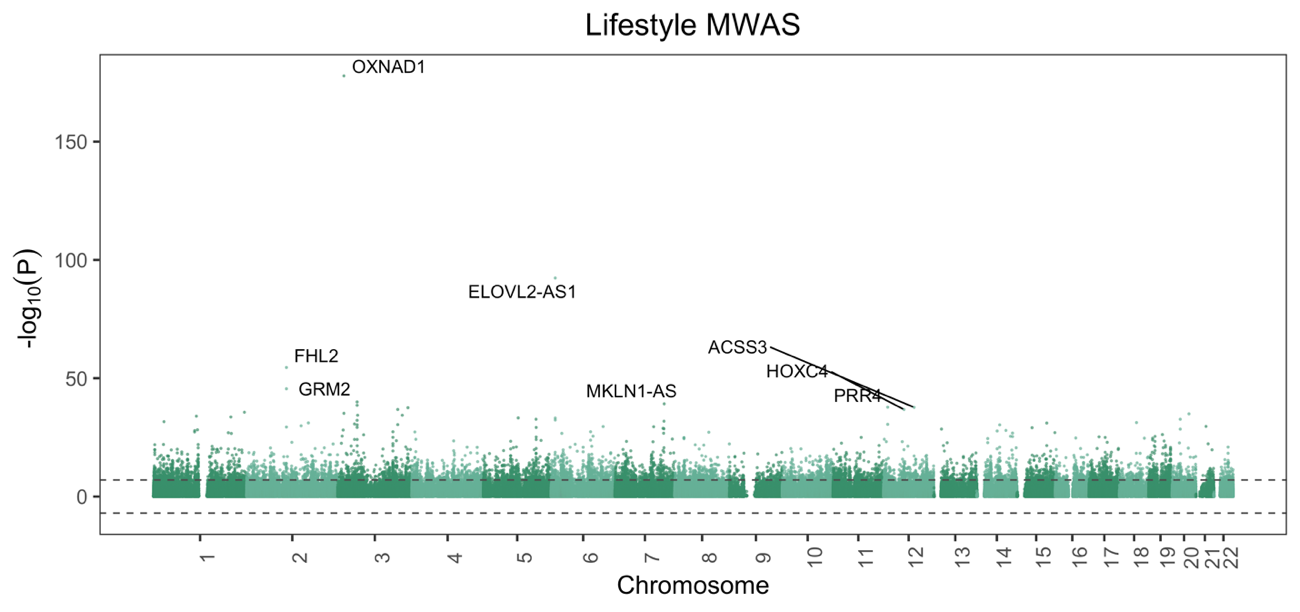


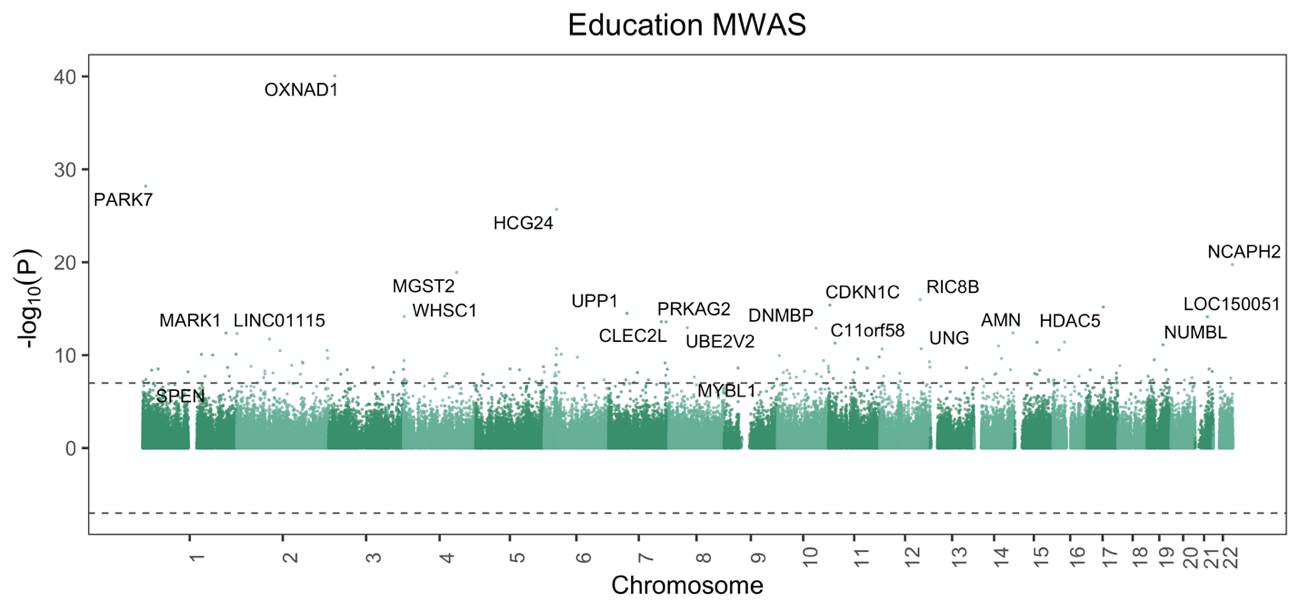


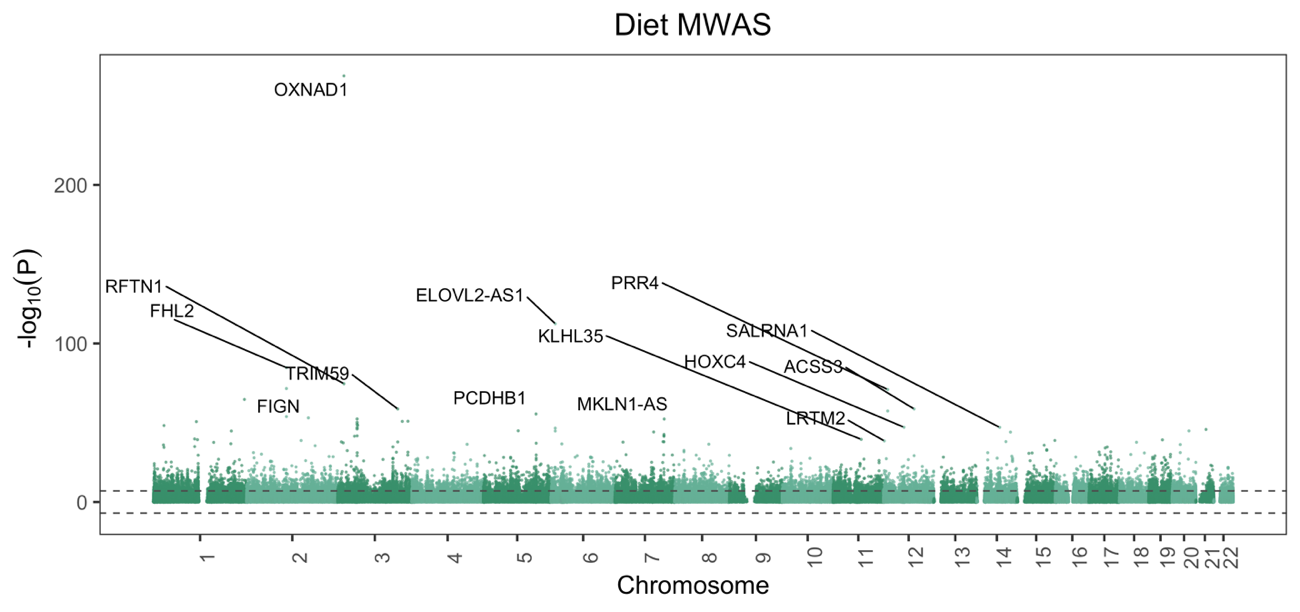


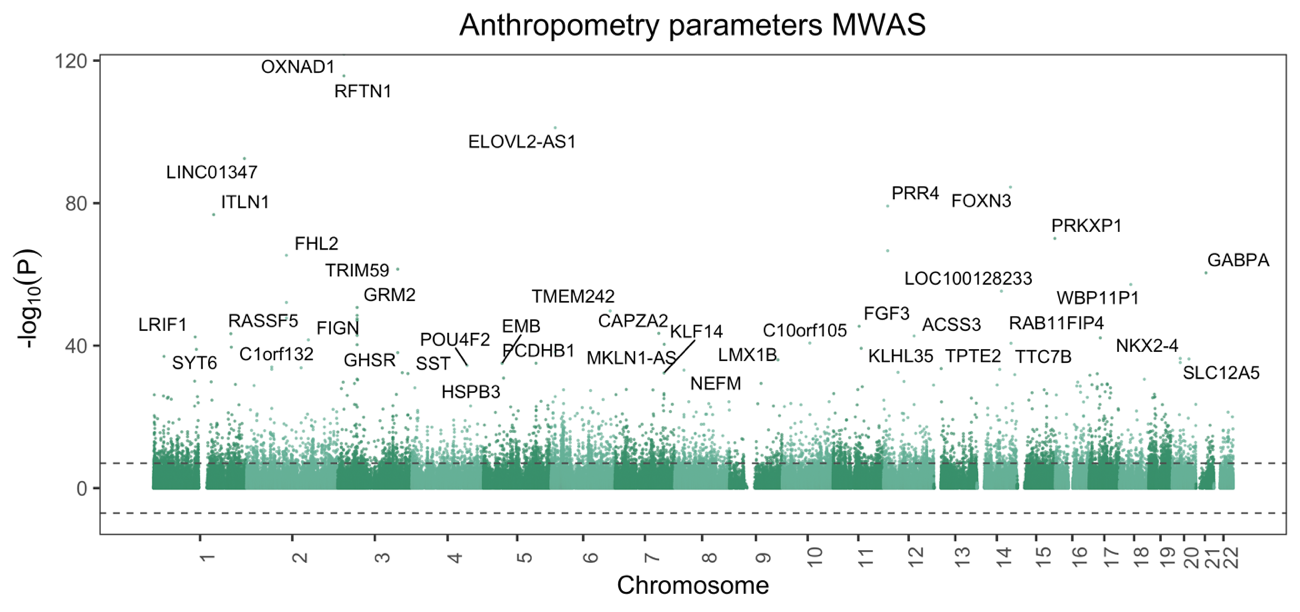


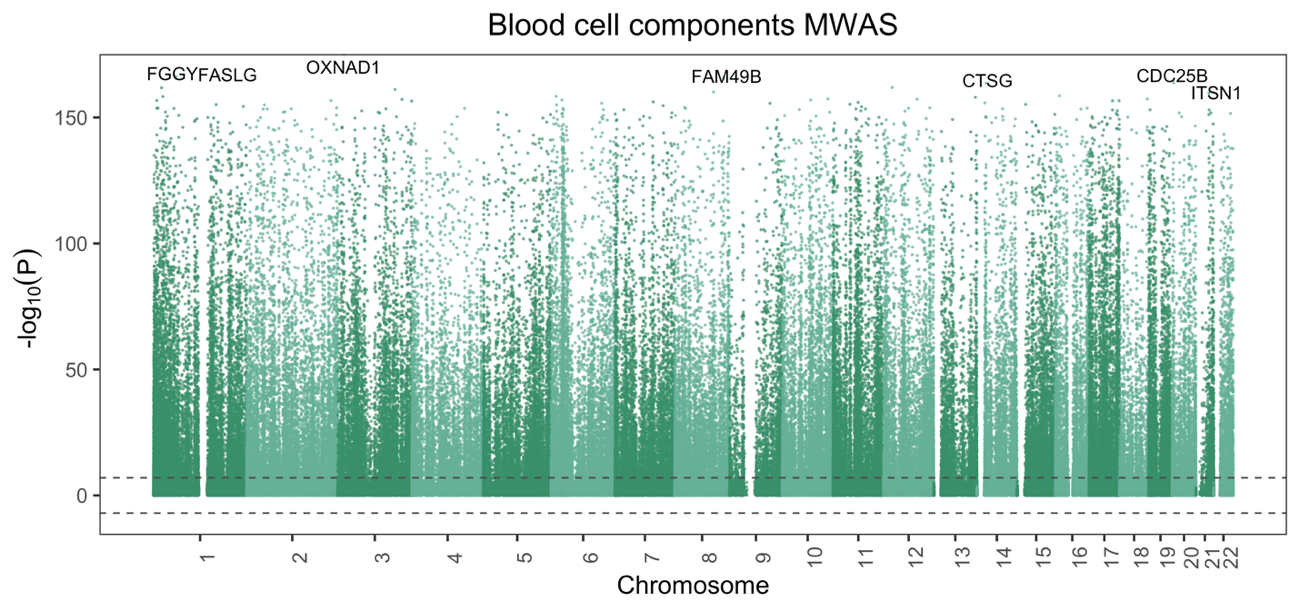


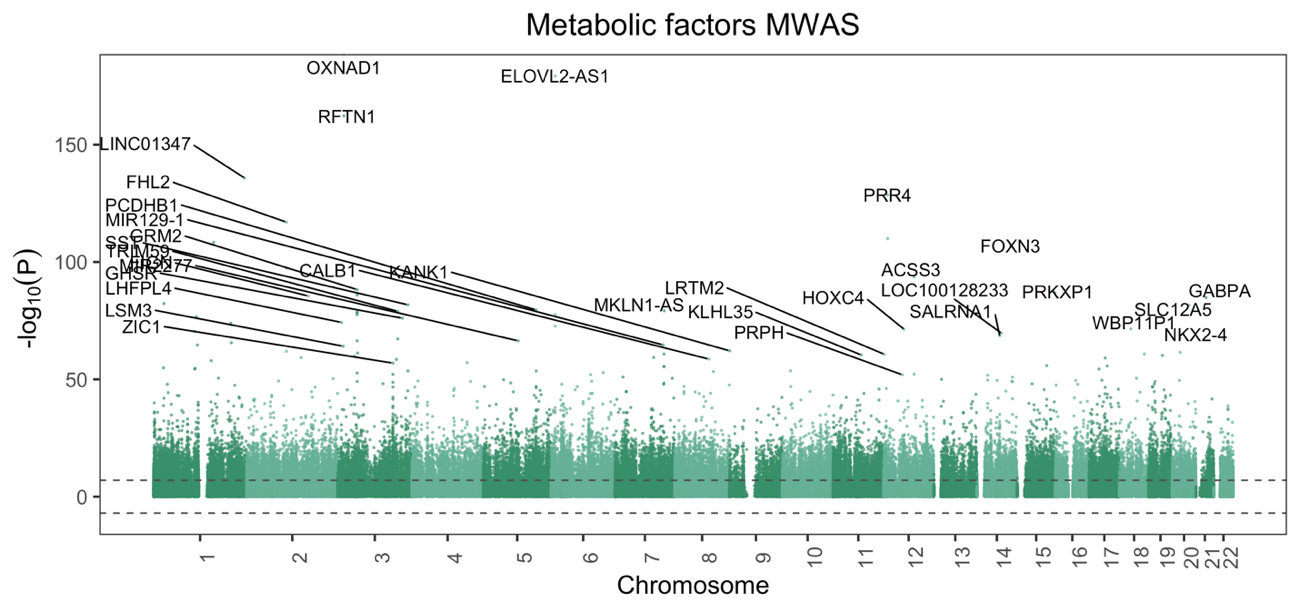


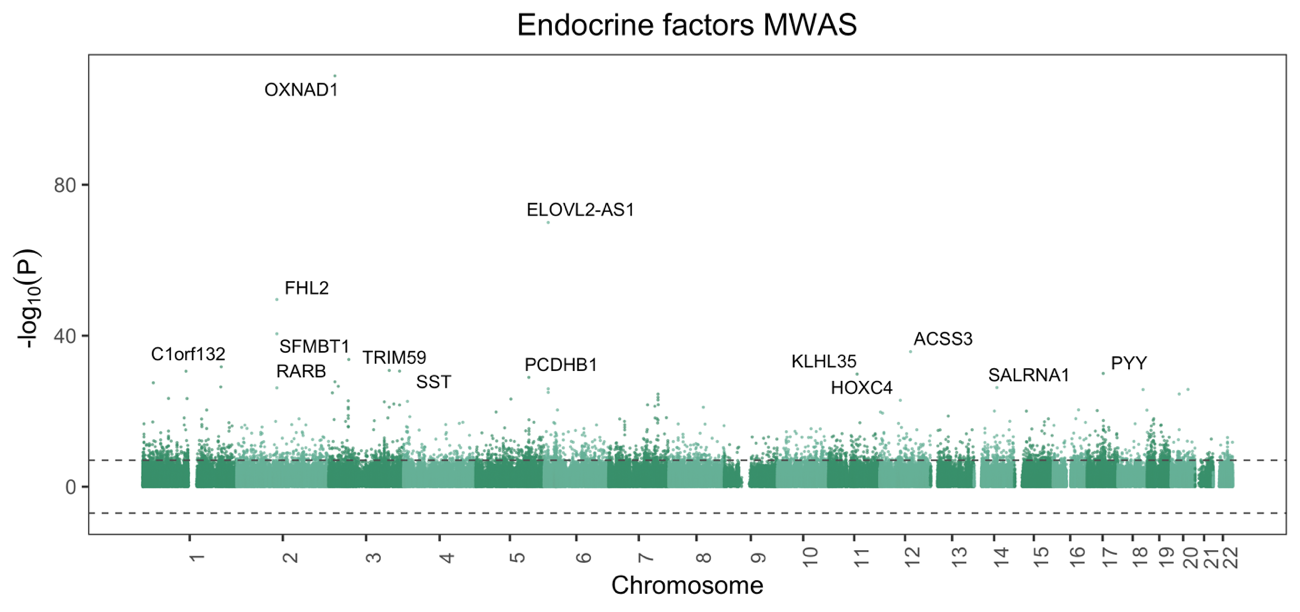


**Supplementary Figure S19:** MWAS analyses for eight phenotype categories, i.e. sun exposure, lifestyle, education, diet, anthropometry, blood cell components, metabolic traits, and endocrine factors. The x-axis denotes the genomic position, and the y-axis denotes the MWAS association $-{log}_{10}$($P$value). The dashed lines indicate the methylome-wide significance thresholds (bacon-adjusted $P$value = $1.12\times{10}^{-7}$). DMSs were annotated to the nearest genes.


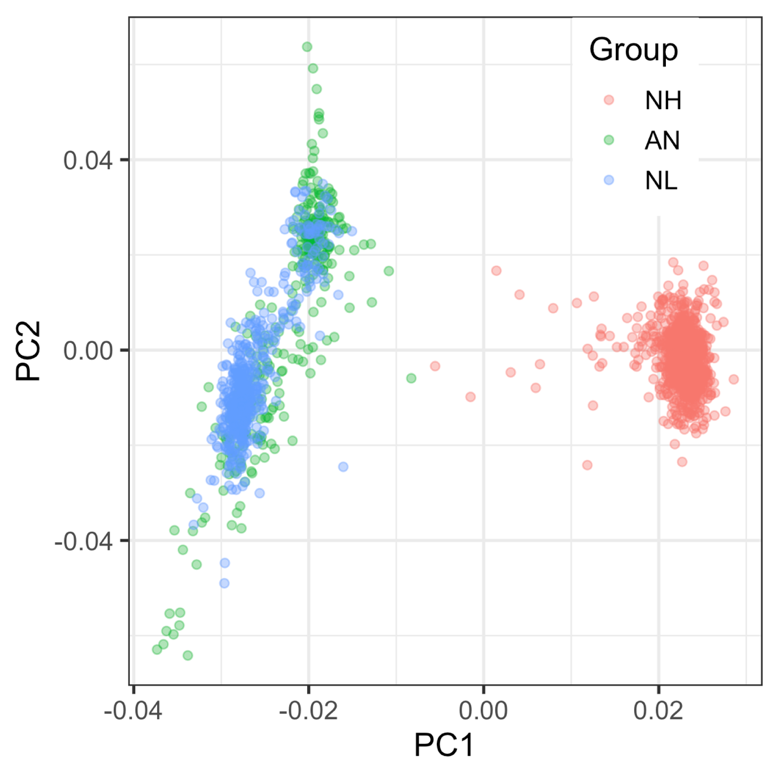


**Supplementary Figure S20:** Scatter plot of genetic principal component analysis. Shown are the result from the principal component analysis (PCA) of QCed 1,622 samples using genotype data of common SNPs. PC1 and PC2 represent the first two eigenvectors from the PCA. ANs (green circle) and NLs (blue circle) were clustered in one group, while NHs (red circle) were clustered in another group.

# **References**

1. Zhang, F. *et al.* OSCA: a tool for omic-data-based complex trait analysis. *Genome Biology* **20**, 107 (2019).

2. van Iterson, M., van Zwet, E.W., Heijmans, B.T. & the, B.C. Controlling bias and inflation in epigenome- and transcriptome-wide association studies using the empirical null distribution. *Genome Biology* **18**, 19 (2017).

3. Childebayeva, A. *et al.* Genome-Wide DNA Methylation Changes Associated With High-Altitude Acclimatization During an Everest Base Camp Trek. *Frontiers in physiology* **12**, 660906-660906 (2021).

4. Alkorta-Aranburu, G. *et al.* The genetic architecture of adaptations to high altitude in Ethiopia. *PLoS Genet* **8**, e1003110 (2012).

5. Bollepalli, S., Korhonen, T., Kaprio, J., Anders, S. & Ollikainen, M. EpiSmokEr: a robust classifier to determine smoking status from DNA methylation data. *Epigenomics* **11**, 1469-1486 (2019).

6. Bai, J., Li, L., Li, Y. & Zhang, L. Genetic and immune changes in Tibetan high-altitude populations contribute to biological adaptation to hypoxia. *Environmental Health and Preventive Medicine* **27**, 39-39 (2022).

7. Facco, M. *et al.* Modulation of immune response by the acute and chronic exposure to high altitude. *Med Sci Sports Exerc* **37**, 768-74 (2005).

8. Ermolao, A. *et al.* Relationship between stress hormones and immune response during high-altitude exposure in women. *Journal of Endocrinological Investigation* **32**, 889-894 (2009).

9. Druker, J. *et al.* Role of Hypoxia in the Control of the Cell Cycle. *Int J Mol Sci* **22**(2021).

10. Hubbi, M.E. & Semenza, G.L. Regulation of cell proliferation by hypoxia-inducible factors. *Am J Physiol Cell Physiol* **309**, C775-82 (2015).

11. Semenza, G.L. Hypoxia. Cross talk between oxygen sensing and the cell cycle machinery. *American Journal of Physiology-Cell Physiology* **301**, C550-C552 (2011).

12. Goda, N. *et al.* Hypoxia-inducible factor 1alpha is essential for cell cycle arrest during hypoxia. *Molecular and Cellular Biology* **23**, 359-69 (2003).

13. Manalo, D.J. *et al.* Transcriptional regulation of vascular endothelial cell responses to hypoxia by HIF-1. *Blood* **105**, 659-69 (2005).

14. Carmeliet, P. *et al.* Role of HIF-1alpha in hypoxia-mediated apoptosis, cell proliferation and tumour angiogenesis. *Nature* **394**, 485-90 (1998).

15. High Altitude and Cancer Mortality. *High Altitude Medicine & Biology* **19**, 116-123 (2018).

16. Chandel, N.S. *et al.* Mitochondrial reactive oxygen species trigger hypoxia-induced transcription. *Proceedings of the National Academy of Sciences* **95**, 11715-11720 (1998).

17. Clanton, T.L. Hypoxia-induced reactive oxygen species formation in skeletal muscle. *Journal of Applied Physiology* **102**, 2379-2388 (2007).

18. de Jager, T.L., Cockrell, A.E. & Du Plessis, S.S. Ultraviolet Light Induced Generation of Reactive Oxygen Species. in *Ultraviolet Light in Human Health, Diseases and Environment* (ed. Ahmad, S.I.) 15-23 (Springer International Publishing, Cham, 2017).

19. Liu, D. *et al.* Maternal care, hippocampal glucocorticoid receptors, and hypothalamic-pituitary-adrenal responses to stress. *Science* **277**, 1659-62 (1997).

20. Francis, D., Diorio, J., Liu, D. & Meaney, M.J. Nongenomic transmission across generations of maternal behavior and stress responses in the rat. *Science* **286**, 1155-8 (1999).

21. Martinez-Arguelles, D.B. & Papadopoulos, V. Epigenetic regulation of the expression of genes involved in steroid hormone biosynthesis and action. *Steroids* **75**, 467-476 (2010).

22. Barnholt, K.E. *et al.* Endocrine responses to acute and chronic high-altitude exposure (4,300 meters): modulating effects of caloric restriction. *American Journal of Physiology-Endocrinology and Metabolism* **290**, E1078-E1088 (2006).

23. Sawhney, R.C., Malhotra, A.S. & Singh, T. Glucoregulatory hormones in man at high altitude. *European Journal of Applied Physiology and Occupational Physiology* **62**, 286-291 (1991).

24. Leon-Velarde, F. *et al.* The role of menopause in the development of chronic mountain sickness. *American Journal of Physiology-Regulatory, Integrative and Comparative Physiology* **272**, R90-R94 (1997).

25. Joseph, V. *et al.* Dopaminergic metabolism in carotid bodies and high-altitude acclimatization in female rats. *American Journal of Physiology-Regulatory, Integrative and Comparative Physiology* **282**, R765-R773 (2002).

26. León-Velarde, F., Rivera-Chira, M., Tapia, R., Huicho, L. & Monge, C.C. Relationship of ovarian hormones to hypoxemia in women residents of 4,300 m. *Am J Physiol Regul Integr Comp Physiol* **280**, R488-93 (2001).

27. Berridge, M.J., Lipp, P. & Bootman, M.D. The versatility and universality of calcium signalling. *Nature Reviews Molecular Cell Biology* **1**, 11-21 (2000).

28. Shimoda, L.A. & Undem, C. Interactions between calcium and reactive oxygen species in pulmonary arterial smooth muscle responses to hypoxia. *Respiratory physiology & neurobiology* **174**, 221-9 (2010).

29. Wang, Y.X. & Zheng, Y.M. ROS-dependent signaling mechanisms for hypoxic Ca(2+) responses in pulmonary artery myocytes. *Antioxid Redox Signal* **12**, 611-23 (2010).

30. Bianba, B. *et al.* Anthropometric Measures of 9- to 10-Year-Old Native Tibetan Children Living at 3700 and 4300 m Above Sea Level and Han Chinese Living at 3700 m. *Medicine (Baltimore)* **94**, e1516 (2015).

31. Dang, S., Yan, H. & Yamamoto, S. High altitude and early childhood growth retardation: new evidence from Tibet. *European Journal of Clinical Nutrition* **62**, 342-348 (2008).

32. Weitz, C.A., Garruto, R.M., Chin, C.T. & Liu, J.C. Morphological growth and thorax dimensions among Tibetan compared to Han children, adolescents and young adults born and raised at high altitude. *Annals of human biology* **31**, 292-310 (2004).

33. Islam, S.M.T., Won, J., Khan, M., Mannie, M.D. & Singh, I. Hypoxia-inducible factor-1 drives divergent immunomodulatory functions in the pathogenesis of autoimmune diseases. *Immunology* **164**, 31-42 (2021).

34. McGettrick, A.F. & O’Neill, L.A.J. The Role of HIF in Immunity and Inflammation. *Cell Metabolism* **32**, 524-536 (2020).

35. Scholz, C.C. & Taylor, C.T. Targeting the HIF pathway in inflammation and immunity. *Current Opinion in Pharmacology* **13**, 646-653 (2013).

36. Meehan, R.T. Immune suppression at high altitude. *Annals of emergency medicine* **16**, 974-979 (1987).

37. Mishra, K. & Ganju, L. Influence of high altitude exposure on the immune system: a review. *Immunological investigations* **39**, 219-234 (2010).

38. Basnyat, B. & Starling, J.M. Infectious diseases at high altitude. *Microbiology Spectrum* **3**, 3.4. 26 (2015).

39. Mazzeo, R.S. Altitude, exercise and immune function. *Exerc Immunol Rev* **11**, 16 (2005).

40. Arima, H. *et al.* High prevalence of rheumatoid arthritis and its risk factors among Tibetan highlanders living in Tsarang, Mustang district of Nepal. *Journal of Physiological Anthropology* **41**, 12 (2022).

41. Gibson, G.E., Pulsinelli, W., Blass, J.P. & Duffy, T.E. Brain dysfunction in mild to moderate hypoxia. *The American Journal of Medicine* **70**, 1247-1254 (1981).

42. Giannopoulou, I., Pagida, M.A., Briana, D.D. & Panayotacopoulou, M.T. Perinatal hypoxia as a risk factor for psychopathology later in life: the role of dopamine and neurotrophins. *Hormones* **17**, 25-32 (2018).

43. Hu, X., Rea, H.C., Wiktorowicz, J.E. & Perez-Polo, J.R. Proteomic analysis of hypoxia/ischemia-induced alteration of cortical development and dopamine neurotransmission in neonatal rat. *J Proteome Res* **5**, 2396-404 (2006).

44. Brandon, A. *et al.* Prenatal hypoxia alters the early ontogeny of dopamine neurons. *Transl Psychiatry* **12**, 238 (2022).

45. Revah, O., Lasser-Katz, E., Fleidervish, I.A. & Gutnick, M.J. The earliest neuronal responses to hypoxia in the neocortical circuit are glutamate-dependent. *Neurobiol Dis* **95**, 158-67 (2016).

46. Vannucci, R.C., Brucklacher, R.M. & Vannucci, S.J. CSF glutamate during hypoxia-ischemia in the immature rat. *Brain Res Dev Brain Res* **118**, 147-51 (1999).

47. Boycott, H.E., Dallas, M., Boyle, J.P., Pearson, H.A. & Peers, C. Hypoxia suppresses astrocyte glutamate transport independently of amyloid formation. *Biochem Biophys Res Commun* **364**, 100-4 (2007).

48. Wood, J.D. A possible role for gamma-aminobutyric acid in the homeostatic control of brain metabolism under conditions of hypoxia. *Experimental Brain Research* **4**, 81-84 (1967).

49. Winn, H.R., Rubio, R. & Berne, R.M. Brain adenosine concentration during hypoxia in rats. *American Journal of Physiology-Heart and Circulatory Physiology* **241**, H235-H242 (1981).

50. Liu, H. *et al.* Epigenomic and transcriptomic analyses define core cell types, genes and targetable mechanisms for kidney disease. *Nature Genetics* **54**, 950-962 (2022).

51. Taylor, D.L. *et al.* Integrative analysis of gene expression, DNA methylation, physiological traits, and genetic variation in human skeletal muscle. *Proceedings of the National Academy of Sciences* **116**, 10883-10888 (2019).

52. Qi, T. *et al.* Identifying gene targets for brain-related traits using transcriptomic and methylomic data from blood. *Nature Communications* **9**, 2282 (2018).

53. Park, J. *et al.* Methylation quantitative trait loci analysis in Korean exposome study. *Molecular & Cellular Toxicology* **16**, 175-183 (2020).

54. Min, J.L. *et al.* Genomic and phenotypic insights from an atlas of genetic effects on DNA methylation. *Nature Genetics* **53**, 1311-1321 (2021).

55. McRae, A.F. *et al.* Identification of 55,000 Replicated DNA Methylation QTL. *Scientific Reports* **8**, 17605 (2018).

56. Delaneau, O. *et al.* A complete tool set for molecular QTL discovery and analysis. *Nature Communications* **8**, 15452 (2017).

57. Horvath, S. DNA methylation age of human tissues and cell types. *Genome Biology* **14**, 3156 (2013).

58. Hannum, G. *et al.* Genome-wide Methylation Profiles Reveal Quantitative Views of Human Aging Rates. *Molecular Cell* **49**, 359-367 (2013).

59. Levine, M.E. *et al.* An epigenetic biomarker of aging for lifespan and healthspan. *Aging* **10**, 573-591 (2018).

60. Simpson, D.J. & Chandra, T. Epigenetic age prediction. *Aging Cell* **20**, e13452 (2021).

61. Jiang, L. *et al.* A resource-efficient tool for mixed model association analysis of large-scale data. *Nat Genet* **51**, 1749-1755 (2019).
